# Using multiple lines of evidence to assess the risk of ecosystem collapse

**DOI:** 10.1098/rspb.2017.0660

**Published:** 2017-09-20

**Authors:** Lucie M. Bland, Tracey J. Regan, Minh Ngoc Dinh, Renata Ferrari, David A. Keith, Rebecca Lester, David Mouillot, Nicholas J. Murray, Hoang Anh Nguyen, Emily Nicholson

**Affiliations:** 1 Deakin University, Australia, School of Life and Environmental Sciences, Centre for Integrative Ecology, Burwood, 3121, Victoria, Australia; 2 School of BioSciences, The University of Melbourne, Parkville, 3010, Victoria, Australia; 3 The Arthur Rylah Institute for Environmental Research, the Department of Environment, Land, Water and Planning, 123 Brown Street, Heidelberg, 3084, Victoria, Australia; 4 Research Computing Centre, University of Queensland, St Lucia, 4072, Queensland, Australia; 5 Coastal and Marine Ecosystems Group, School of Life and Environmental Sciences, The University of Sydney, Sydney, New South Wales, Australia; 6 Centre for Ecosystem Science, School of Biological, Earth and Environmental Science, University of New South Wales, Kensington, 2052, New South Wales, Australia; 7 New South Wales Office of Environment and Heritage, Hurstville, 2220, New South Wales, Australia; 8 Long Term Ecological Research Network, Terrestrial Ecosystem Research Network, Australian National University, Canberra, 0200, Australian Capital Territory, Australia; 9 Deakin University, Australia, Centre for Regional and Rural Futures, Geelong, 3220, Victoria, Australia; 10 UMR 5119—Écologie des Systèmes marins côtiers, Université Montpellier 2, Montpellier Cedex 5, France; 11 ARC Centre of Excellence for Coral Reef Studies, James Cook University, Townsville, 4881, Queensland, Australia

**Keywords:** ecosystem collapse, coral reefs, stochastic model, indicators, IUCN Red List of Ecosystems, Meso-American Reef

## Abstract

Effective ecosystem risk assessment relies on a conceptual understanding of ecosystem dynamics and the synthesis of multiple lines of evidence. Risk assessment protocols and ecosystem models integrate limited observational data with threat scenarios, making them valuable tools for monitoring ecosystem status and diagnosing key mechanisms of decline to be addressed by management. We applied the IUCN Red List of Ecosystems criteria to quantify the risk of collapse of the Meso-American Reef, a unique ecosystem containing the second longest barrier reef in the world. We collated a wide array of empirical data (field and remotely sensed), and used a stochastic ecosystem model to backcast past ecosystem dynamics, as well as forecast future ecosystem dynamics under 11 scenarios of threat. The ecosystem is at high risk from mass bleaching in the coming decades, with compounding effects of ocean acidification, hurricanes, pollution and fishing. The overall status of the ecosystem is Critically Endangered (plausibly Vulnerable to Critically Endangered), with notable differences among Red List criteria and data types in detecting the most severe symptoms of risk. Our case study provides a template for assessing risks to coral reefs and for further application of ecosystem models in risk assessment.

## 1. Introduction

Ecosystems around the world face degradation and collapse as a result of environmental and human-induced changes. Ecosystem collapse may involve large losses of biodiversity, ecosystem functions and services, as well as societal structures [1]. Understanding the risk that ecosystem collapses will occur is a fundamental requisite for conservation planning and adaptation to environmental change.

Two tools are commonly used in biodiversity risk assessment: generic risk assessment protocols and stochastic simulation models. Risk assessment protocols—such as the International Union for Conservation of Nature (IUCN) Red List of Ecosystems [2]—assign ecosystems to ordinal categories of risk based on decision rules. Risk assessment protocols use multiple symptoms to assess risk, such as ecosystem distribution size and rates of decline in distribution size and/or ecological function [3]. Risk assessment protocols are widely applicable, with hundreds of ecosystems assessed in countries as diverse as Finland, South Africa and Australia [3], including in data-poor circumstances [2]. However, most risk assessment protocols take limited account of interactions among threats, and are unable to fully integrate spatial and functional trajectories of ecosystem decline [4].

Ecosystem simulation models are used to quantitatively estimate risk based on a mechanistic understanding of ecosystem dynamics, future threats and social–ecological relationships [5]. Some ecosystem models can integrate functional and spatial patterns of decline with information on multiple threats, thereby providing a detailed understanding of ecosystem responses to changing environments and human pressures [5]. These models may be used to forecast ecosystem dynamics under various scenarios [5] and backcast dynamics in data-poor situations [6]. They can also be used to test the reliability of indicators used in management and to diagnose key mechanisms of ecological change [7]. However, ecosystem models often demand large quantities of data and are only accessible to a narrow community of scientists, constraining their use to few ecosystems [8]. Adapting existing ecosystem models to new research questions or management objectives—such as risk assessment—also poses challenges [8].

Risk assessment protocols and ecosystem models are complementary rather than alternative approaches to risk assessment. IUCN Red List of Ecosystems (RLE) assessments rely on a mechanistic understanding of ecosystem dynamics, usually depicted by a conceptual model that summarizes key ecosystem processes to risk managers, conservation practitioners, and the wider community [2]. The conceptual model informs the selection of indicators to assess functional declines and underpins the development of ecosystem models [4]. The RLE protocol incorporates quantitative estimates of risk based on stochastic ecosystem models (criterion E), a process analogous to the use of population viability analyses for species [2]. For instance, Burns *et al*. [9] predicted a very high likelihood (≥92%) of collapse for the mountain ash forest in Australia under 39 harvesting and fire regime scenarios. Their model formed part of a comprehensive RLE assessment, in which modelled estimates of collapse complemented assessments based on spatial distribution, declines in distribution and declines in ecological function [9]. Ecosystem models remain underused in ecosystem risk assessment, with only two RLE assessments applying criterion E to date [2,9]. There is therefore a clear need to adapt existing ecosystem models for use in risk assessment, as well as provide guidance on how to assess risks to ecosystems with models.

**Figure 1.**
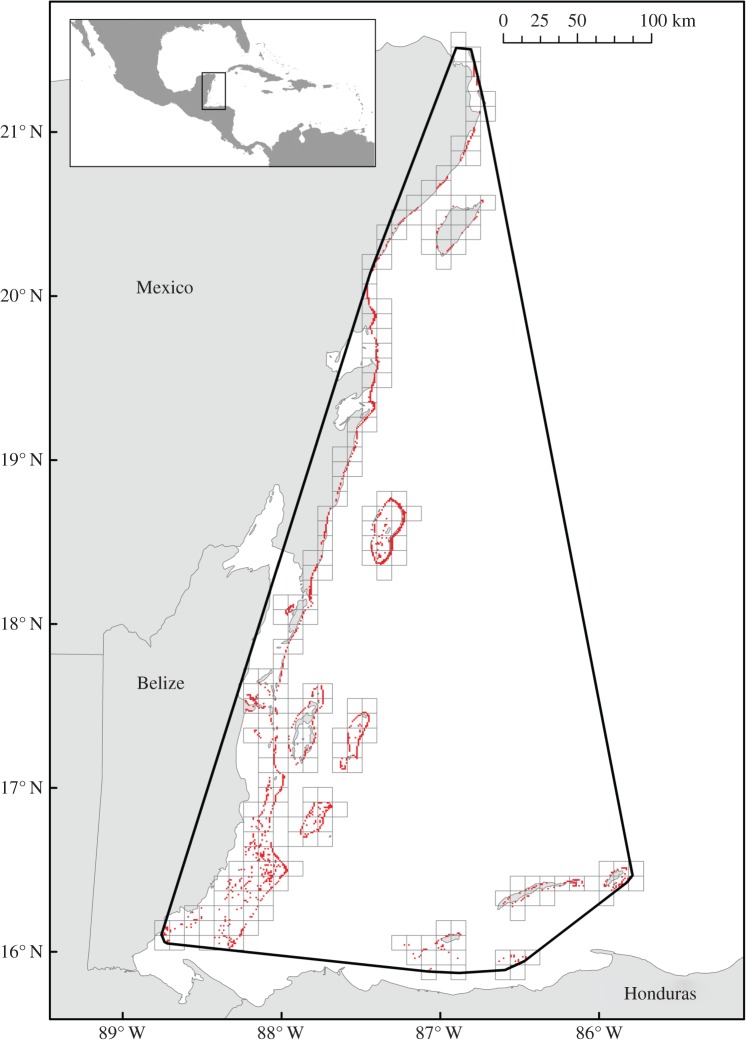
The mapped distribution of the Meso-American Reef and assessment under criterion B of the IUCN Red List of Ecosystems. Red squares indicate cells occupied by reef at a 1 km^2^ resolution. The thick black line indicates the minimum convex polygon enclosing all reef occurrences. Black grid squares indicate 10 × 10 km grid cells. (Online version in colour.)

Coral reefs are ideal ecosystems to investigate the use of ecosystem models in risk assessment, as they are biologically and economically important [10], vulnerable to a range of interacting threats [11], and extensively modelled [7]. We use a ‘whole-of-ecosystem’ model, the Coral Reef Scenario Evaluation Tool [12], to assess risks to the Meso-American Reef (MAR). The MAR contains the second longest barrier reef in the world, and extends more than 1000 km from Mexico to Belize, Guatemala and northern Honduras (figure 1). The MAR has been affected by multiple threats over the last 50 years, including hurricanes, lionfish invasion, overfishing, pollution, ocean acidification, rising sea surface temperatures, and disease outbreaks among urchins and corals [12,13]. As in many coral reefs around the world, threats are predicted to increase in the future [11], so there is an urgent need to understand interactions among threats and evaluate potential levers for management [14].

We collated and analysed a wide array of empirical data (field and remotely sensed), and used a stochastic ecosystem model to assess the MAR with the RLE criteria [2,4]. The RLE lists ecosystems in eight categories of risk largely mirrored on the IUCN Red List of Threatened Species, including three threatened categories (Vulnerable, Endangered, and Critically Endangered) defined by quantitative criteria. Two criteria assess spatial symptoms of ecosystem collapse: declines in spatial distribution (criterion A) and small distribution size (criterion B). Two criteria assess functional symptoms, namely environmental degradation (criterion C) and biotic disruption (criterion D). Declines in spatial distribution, environmental degradation and biotic disruption (criteria A, C and D) are measured over three time frames: the past 50 years (subcriterion 1), the next 50 years (subcriterion 2a) and since the pre-industrial period (subcriterion 3). Finally, criterion E evaluates quantitative estimates of the risk of collapse over the next 50–100 years.

We use the relatively data-rich example of the MAR to explore how synthesizing multiple lines of evidence with a stochastic ecosystem model can inform ecosystem risk assessment and threat diagnosis. In doing so, we provide practical guidance for assessing risks to ecosystems around the world with ecosystem models, with a focus on coral reefs.

## 2. Methods

### (a) Ecosystem model

**Figure 2.**
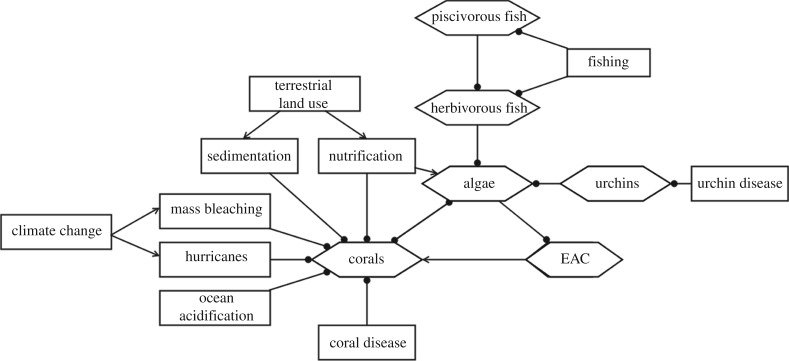
Conceptual model of ecological processes relevant to the risk assessment of the Meso-American Reef. Some ecological processes included in the ecosystem model are not depicted here. The macroalgae and turf benthic groups from the ecosystem model are depicted jointly as algae. The brooding and spawning corals from the ecosystem model are depicted jointly as corals. EAC: epilithic algal communities. Pointed arrows indicate positive effects, whereas rounded arrows indicate negative effects.

The Coral Reef Scenario Evaluation Tool is a stochastic ecosystem model that focuses on five benthic groups (brooding corals, spawning corals, macroalgae, turf and epilithic algal communities) and four consumer groups (herbivorous fish, small piscivorous fish, large piscivorous fish and urchins) [12] (figure 2). Functional groups interact through spatial patterns of recruitment, dispersal, foraging and competition. The model is updated weekly and run on 2 × 2 km grid cells. Model dynamics are well understood, including model sensitivity and uncertainty [15], and behaviour under future scenarios [14].

First, we recreated pre-human reef dynamics over a 100-year period, only including disturbance from hurricanes based on historical levels (electronic supplementary material, appendix S1). This enabled us to produce stable model trajectories over long time frames, and to investigate the effect of initial values on model behaviour. The model was parameterized with data from historical studies (1970s) and contemporary data from sites in ‘very good’ condition according to the Reef Health Index (electronic supplementary material, appendix S1) [16]. There was little difference in community composition among parameterizations, so we used sites in very good condition to instantiate the historical reconstruction.

Second, we assessed the ability of the model to recreate known ecosystem dynamics based on disturbances occurring over the period 1966–2015 (historical reconstruction; electronic supplementary material, appendix S1). We validated the ecosystem model by collating empirical data (116 survey observations over the 50-year period) on coral cover, herbivorous fish biomass, and piscivorous fish biomass (electronic supplementary material, appendix S2). We assessed model performance against empirical data with root mean squared error, average absolute error, Spearman rank correlation and modelling efficiency (electronic supplementary material, appendix S1) [6]. We used ten model runs with a 5-year burn-in period for both the initial reef scenarios and the historical reconstruction [12]. Because the model includes fast-growing *Acropora* species, which have become uncommon in the MAR [17], we repeated the initial reef scenarios and the historical reconstruction with a lower estimate of coral growth rates excluding *Acropora* species (electronic supplementary material, appendix S1). The historical reconstruction exhibited worse performance metrics, probably because growth rates were reduced for the whole simulation period, when in reality *Acropora* species only declined in abundance in the late 1980s. Because *Acropora* species are still present in the MAR and the higher growth rate parametrization showed better performance metrics, we projected future ecosystem dynamics with the higher growth rate parametrization, noting that this parametrization may over-estimate coral cover.

Third, we used the ecosystem model to project ecosystem dynamics from 2016 to 2115 and assess interactions among threats. We developed 11 scenarios based on low and high levels of five threats: fishing, pollution (sedimentation and nutrification), mass bleaching, ocean acidification and hurricanes (table 1). We did not include coral diseases in our scenarios due to a lack of adequate future projections. Increases in at least one of mass bleaching, hurricanes and/or ocean acidification are likely in the next 50 years [21,24,25], so scenarios 5–11 were considered most likely. We used the most likely scenarios to derive risk categories and plausible bounds under criteria A2a, D2a and E [2]. We instantiated the model with empirical data on benthic cover and consumer biomass collected in 2013 (electronic supplementary material, appendix S2). For each scenario, we conducted 500 Monte Carlo runs of 100 years each with 5 years burn-in, parallelized with Nimrod [26].

**Table 1.** Definition of threat levels and scenarios used to project ecosystem dynamics over the next 100 years (2016–2115) in the Meso-American Reef.

	threat level			scenario number										
	low (−)	high (+)	rationale	1	2	3	4	5	6	7	8	9	10	11
fishing	0.08–2.54 g/m^2^/year	2.54–5.0 g/m^2^/year	minimum to median range of current fishing levels, versus median to maximum of current levels [12]	−	−	+	+	−	−	+	+	−	−	−
pollution	−25%	+50%	linear decrease or increase in sedimentation and nutrification over 100 years, compared to current pollution levels; based on terrestrial land use scenarios [18]	−	+	−	+	−	+	−	+	−	−	−
mass bleaching	1 in 20 years	1 in 5 years	predictions of mass coral bleaching dependent on the capacity of corals to adapt (electronic supplementary material, appendix S1) [19]; coral mortality was sampled from a uniform distribution (0.002–0.6; [20])	−	−	−	−	+	+	+	+	+	−	−
ocean acidification	0.04–0.2/year	0.032–0.16/year	current coral growth rate, versus 21% decrease coral growth rate predicted by a decrease in aragonite saturation over 100 years [21,22]	−	−	−	−	+	+	+	+	−	+	−
hurricanes	6% of hurricanes in categories 4 and 5	20% of hurricanes in categories 4 and 5	current hurricane frequency and severity, versus predicted linear increase in prevalence of hurricanes categories 4 and 5, with overall hurricane frequency remaining constant [23]; mortality rates were sampled from uniform distributions (coral: 0.002–0.7; macroalgae: 0–0.9) and number of reef cells affected were scaled by hurricane category (electronic supplementary material, table S6)	−	−	−	−	+	+	+	+	−	−	+

### (b) Ecosystem collapse

To estimate risk, it is necessary to define the endpoint of ecosystem decline (i.e. the point at which an ecosystem is considered collapsed) [4]. Within the RLE, ‘an ecosystem is Collapsed when it is virtually certain that its defining biotic or abiotic features are lost from all occurrences, and the characteristic native biota are no longer sustained’ [4]. Coral cover is a commonly used indicator of coral reef state [17] and is the most readily available indicator in the MAR [13]. We considered the MAR to be collapsed when live coral cover declined to less than 1% throughout the mapped ecosystem distribution, and defined collapse thresholds for environmental indicators based on required levels to reach a coral cover less than 1% (electronic supplementary material, appendix S1). Fish are key components of the reef ecosystem [27], so we defined collapse thresholds for herbivorous fish as 5 g m^−2^, and for (small and large) piscivorous fish biomass as 2 g m^−2^, based on the Reef Health Index (electronic supplementary material, appendix S1) [16]. The outcome of ecosystem risk assessment can be sensitive to the selection of collapse thresholds [2,9]. We present results for sensitivity analyses in electronic supplementary material, appendix S1, based on minimum collapse thresholds representing functional extinction (0% coral cover; 0 g m^−2^ herbivorous fish biomass; and 0 g m^−2^ piscivorous fish biomass), and high collapse thresholds based on the ‘critical’ category of the Reef Health Index (5% coral cover; 9.6 g m^−2^ herbivorous fish biomass; and 4.2 g m^−2^ piscivorous fish biomass) [16].

### (c) Spatial criteria: decline in distribution (criterion A) and small distribution size (criterion B)

We applied the RLE criteria according to IUCN guidelines [4], briefly summarized in electronic supplementary material, appendix S1. We outline our methods below and provide a comprehensive account in electronic supplementary material, appendix S1. Criterion A identifies ecosystems that are undergoing declines in area, most commonly due to threats resulting in ecosystem loss and fragmentation [4]. Measuring past changes in the spatial distribution of the MAR is challenging, due to the paucity of processed remote sensing data for the ecosystem. To assess future changes in distribution (subcriterion A2a), we predicted the future ecosystem distribution with the ecosystem model under 11 scenarios (table 1). We excluded grid cells meeting the definition of collapse (less than 1% coral cover) and assumed that future live coral cover could not extend beyond currently mapped grid cells.

Criterion B assesses ecosystems against fixed thresholds of distribution size to identify ecosystems at risk of spatially explicit threats [4,28]. Criterion B requires information on (i) extent of occurrence (EOO), (ii) area of occupancy (AOO) and (iii) the number of threat-based locations. To quantify EOO we calculated the area of a minimum convex polygon around all coral occurrences, based on mapped reef locations at 1 km^2^ grain size [12] derived from the Millennium Coral Reef Mapping Project (from 30 m Landsat imagery [29]; figure 1). We calculated AOO using 10 × 10 km grid cells, including all grid cells that contained occurrences of the ecosystem [4] (figure 1). A threat-based location is defined as a geographically or ecologically distinct area in which a single threat can rapidly affect occurrences of the ecosystem [4]. Numbers of locations were estimated for each significant threat likely to cause collapse of the MAR over a short time period (approx. 20 years; electronic supplementary material, appendix S1).

### (d) Functional criteria: environmental degradation (criterion C) and biotic disruption (criterion D)

The application of criteria C and D requires the relative severity of decline in key ecosystem indicators to be estimated. Relative severity describes the percentage change observed in an indicator scaled between two values: one value describing the initial state of the system (0% change) and one describing a collapsed state (100% change). Information on relative severity is combined with information on the proportion of the ecosystem affected to determine the risk category [4]. We devised a five-step checklist for candidate indicators: (i) assess relevance to ecosystem processes, (ii) assess data availability and quality, (iii) identify a suitable threshold representing ecosystem collapse, (iv) estimate initial, current or future values, and (v) characterize the shape of decline (electronic supplementary material, appendix S1).

Criterion C identifies ecosystems that are undergoing environmental degradation [4]. We identified four environmental processes influencing live coral cover: sea surface temperature, ocean acidification, hurricane frequency and intensity, and pollution (sedimentation and nutrification) (figure 2). We used blended monthly sea surface temperature data available since 1871 and projected to 2099 [19] to derive degree heating months, an indicator of mass bleaching [25]. Corals recover from mass bleaching events if intervals between events are sufficiently long (more than 5 years [30]), so we used the annual probability of bleaching (*p* = 0.2) calculated over running 10-year intervals as threshold for ecosystem collapse [30]. We used sea surface aragonite saturation (*Ω*_arag_) as an indicator of ocean acidification, projected in the Caribbean back to pre-industrial times and forward to the year 2100 [21]. Surface water *Ω*_arag_ values of less than 3 have been described as ‘extremely marginal’ for reef growth [31], so we used *Ω*_arag_ = 3 as the threshold for ecosystem collapse.

We obtained International Best Track Archive for Climate Stewardship records for hurricanes categories 1–5 on the Saffir–Simpson scale between 1853 and 2015 [32]. There is no evidence of recovery to a pre-disturbance state for at least 8 years post-hurricane in the Caribbean [33], so we defined the collapse threshold as a hurricane frequency of one in 8 years for hurricane categories 1–5, and one in 12 years for categories 4–5 only (electronic supplementary material, appendix S1). To assess the effects of sedimentation and nutrification, we searched for field data on sedimentation rate, nutrient concentration, salinity and water transparency, and reviewed recent modelling studies and geochemical studies. However, none of the data sources were appropriate to assess the effects of sedimentation and nutrification under criterion C (electronic supplementary material, appendix S1).

Criterion D identifies ecosystems that are undergoing loss or disruption of key biotic processes maintaining the characteristic native biota [4]. We considered several indicators of biotic disruption (figure 2; electronic supplementary material, appendix S1); live coral cover was the only indicator with a suitable time series of empirical observations for assessing changes over 1966–2015 (D1). We used linear weighted regression to predict initial and current live coral cover for the years 1970 and 2013, so as to not extrapolate beyond the empirical time series, and selected models based on changes in AIC. In addition to the empirical time series for coral cover, we used backcast estimates of herbivorous fish biomass and (large and small) piscivorous fish biomass from the ecosystem model to assess changes over 1966–2015. We assessed historical biotic disruption (D3) with the same data as for D1, assuming that there was no change in biotic disruption between the pre-industrial period and 1966. Future declines were assessed for coral cover, herbivorous fish biomass and (large and small) piscivorous fish biomass with model projections from 2016 to 2065 under 11 scenarios (D2a; table 1).

### (e) Criterion E: quantitative risk analysis

Criterion E allows for an integrated assessment of multiple threats and symptoms of collapse with the use of a stochastic ecosystem model [4]. We computed the probability of ecosystem collapse over the next 50 and 100 years for each scenario by counting the number of model runs meeting the collapse threshold for each of the three biotic indicators (coral cover, herbivorous fish biomass and piscivorous fish biomass).

## 3. Results

### (a) Ecosystem model

The historical reconstruction indicated good fit with empirical coral cover data (electronic supplementary material, table S9 and figure S16). The model successfully reproduced patterns of decline in coral cover due to severe hurricanes in 1988, 2005 and 2007, and the large decline in cover in 1998 due to both hurricanes and disease.

### (b) Spatial criteria: decline in distribution (criterion A) and small distribution size (criterion B)

Due to the absence of remotely-sensed information on past changes in distribution for the ecosystem, we assessed subcriteria A1 and A3 as Data Deficient (table 2). Based on the ecosystem model and the seven most likely scenarios (scenarios 5–11), we estimated future declines in distribution of 4.2 to 26.1% of the current distribution, leading to an assessment under A2a as Least Concern. The extent of occurrence of the MAR is 106 629.5 km^2^ (B1: Least Concern) and the area of occupancy is 231 10 × 10 km grid cells (B2: Least Concern). According to our analysis of future environmental degradation (C2a), pollution, fishing, hurricanes, bleaching and acidification are unlikely to cause the ecosystem to collapse or become Critically Endangered within 20 years (B3: Least Concern).

**Table 2.** Application of the IUCN Red List of Ecosystems criteria for the Meso-American Reef. DD, Data Deficient; LC, Least Concern; NT, Near Threatened; VU, Vulnerable; EN, Endangered; CR, Critically Endangered. Categories in brackets indicate plausible bounds of assessment for each subcriterion.

criterion	declining distribution (A)	restricted distribution (B)	environmental degradation (C)	biotic disruption (D)	quantitative risk analysis (E)
subcriterion 1	DD	LC	EN	EN	EN (LC–EN)^a^
subcriterion 2a	LC (LC–NT)^a^	LC	CR (VU–CR)	CR (VU–CR)^a^	
subcriterion 3	DD	LC	VU	VU	

^a^Indicates that the subcriterion was assessed with the ecosystem model.

### (c) Functional criteria: environmental degradation (criterion C) and biotic disruption (criterion D)

We estimated a relative severity of mass bleaching of 50% in the past 50 years (C1), and 50% since pre-industrial times (C3) (table 3; electronic supplementary material, appendix S1). The relative severity of future mass bleaching over the entire ecosystem was 44–100%, depending on the capacity of corals to adapt to increasing sea surface temperatures. We assessed the ecosystem as Critically Endangered as coral adaptation is uncertain (C2a) [34]. Over the three time frames of our analysis, aragonite saturation declined with relative severities of 22% in the past 50 years (C1) and 30% since pre-industrial times (C3), and was projected to decline by 50% by 2065 (C2a). We estimated a 12% decrease in the relative severity of hurricane frequency in the past 50 years (C1) and a 33% increase in the relative severity of hurricanes categories 4 and 5 in the next 50 years (C2a). There were no significant trends in the North Atlantic hurricane frequency since the late 1800s [24] and hurricane frequency remained below the collapse threshold for that period (C3).

**Table 3.** Application of the IUCN Red List of Ecosystems criteria for environmental degradation (C) and biotic disruption (D) for the Meso-American Reef. For each criterion, the indicator with the highest most likely category is selected for use in the assessment table (table 2). DD, Data Deficient; LC, Least Concern; NT, Near Threatened; VU, Vulnerable; EN: Endangered; CR, Critically Endangered.

	environmental degradation (C)				biotic disruption (D)		
	mass bleaching	ocean acidification	hurricanes	pollution	coral cover	herbivorous fish biomass	piscivorous fish biomass
subcriterion 1 (1966–2015)	EN	LC	LC	DD	EN	LC^a^	EN^a^
subcriterion 2a (2016–2065)	CR (VU–CR)	EN	VU	DD	CR (NT–CR)^a^	EN (EN–CR)^a^	CR (VU–CR)^a^
subcriterion 3 (pre-industrial–2015)	VU	LC	LC	DD	VU	LC^a^	VU^a^

^a^Indicates that the subcriterion was assessed with the ecosystem model.

**Figure 3.**
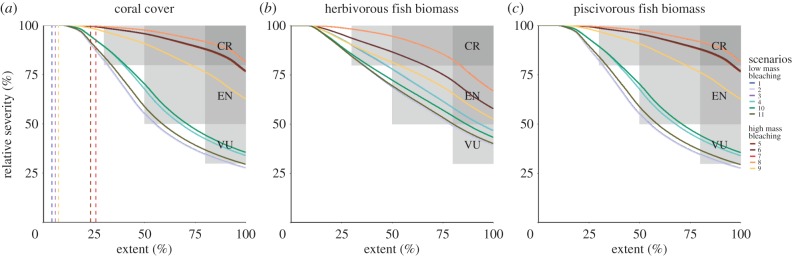
Estimated relative severity of decline in ecological function in the Meso-American Reef over the next 50 years (2016–65), projected with the ecosystem model under 11 scenarios of threat. The full lines indicate the relative severity of decline (percentage change towards collapse) averaged over different extents of the ecosystem (subcriterion D2a), for cells analysed in decreasing order of relative severity. Collapse thresholds are, for each indicator: (*a*) coral cover (1%), (*b*) herbivorous fish biomass (5 g m^−2^) and (*c*) piscivorous fish biomass (2 g m^−2^). The hatched vertical lines in panel (*a*) indicate the decline in spatial extent of the ecosystem under the different scenarios i.e. the per cent of cells in the ecosystem with 100% relative severity of coral cover decline (subcriterion A2a). The shaded boxes indicate the thresholds for the IUCN Red List of Ecosystems categories (dark grey: Critically Endangered; grey: Endangered; light grey: Vulnerable). (Online version in colour.)

Over the last 50 years, the relative severity of decline in coral cover in the MAR was 63.4% based on empirical data and 64.9% based on the model backcast (D1; table 3). We backcast a 2.8% decline in herbivorous fish biomass over the last 50 years (D1). We backcast a 62.4% decline in piscivorous fish biomass over the last 50 years (D1). We assessed biotic declines since the pre-industrial period with the same data as for the last 50 years (D3). Based on the seven likely scenarios of threat (scenarios 5–11), we projected future declines across ≥80% of the extent of the ecosystem of 28.9–93.1% for coral cover, 50.2–82.7% for herbivorous fish biomass and 36.8–81.5% for piscivorous fish biomass (D2a; table 3 and figure 3).

### (d) Criterion E: quantitative risk analysis

Our implementation of scenarios of threats indicated a wide range of collapse probabilities across time frames and indicators (table 4; electronic supplementary material, Figure S19). Four of the seven likely scenarios led to an assessment as Endangered based on coral cover in the next 50 years, leading to an overall assessment as Endangered (Least Concern–Endangered) under criterion E (table 4).

**Table 4.** Probabilities of ecosystem collapse based on scenarios applied to the Meso-American Reef over the next 100 years (2016–2115). See table 1 for scenario descriptions. LC, Least Concern; VU, Vulnerable; EN, Endangered.

	coral cover		herbivorous fish biomass		piscivorous fish biomass	
	50 years	100 years	50 years	100 years	50 years	100 years
scenario 1	0	0	0	0	0	0
scenario 2	0	0	0	0	0	0
scenario 3	0	0	0	0	0	0
scenario 4	0	0	0	0	0	0
scenario 5	0.204	0.658	0	0	0.174	0.586
scenario 6	0.204	0.654	0	0	0.182	0.588
scenario 7	0.234	0.714	0	0	0.272	0.712
scenario 8	0.224	0.742	0	0	0.27	0.722
scenario 9	0.084	0.37	0	0	0.064	0.316
scenario 10	0	0	0	0	0	0
scenario 11	0	0	0	0	0	0
criterion E	EN (LC–EN)		LC		VU (LC–EN)	

## 4. Discussion

The weight of evidence from our analysis supports Critically Endangered status (plausibly Vulnerable to Critically Endangered) for the Meso-American Reef (MAR), primarily based on modelled trends in coral cover and piscivorous fish biomass. The status of the MAR is determined by both past and future declines in ecological function, rather than by its spatial distribution size or future declines in distribution. The expression of distributional symptoms of risk in some types of ecosystems and functional symptoms in others (as well as differences in their measurability) highlights the importance of risk protocols capable of assessing both [3]. The IUCN Red List of Ecosystems (RLE) protocol achieves this through an ensemble of complementary criteria that are sensitive to different symptoms and have different data requirements [2]. Assessment outcomes based on most or all of the five criteria are therefore expected to be more robust than those based on only one or two criteria, particularly if only spatial criteria (A or B) or only functional criteria (C, D or E) are evaluated [4]. Yet, to date, 50% of global RLE assessments lack assessments of functional criteria [35], suggesting that risks of functional declines could be under-estimated.

Our analysis reveals differences in assessment between rule-based criteria and the quantitative analysis. Despite being based on the same simulation outputs, we obtained lower risk categories with the quantitative risk analysis (criterion E) than with the corresponding rule-based criterion (criterion D), implying that rule-based criteria are more precautionary. While the two other existing applications of ecosystem viability analysis found risk levels comparable with other criteria (electronic supplementary material, appendix S2 in [2] [9]), in species assessments, threat categories assigned based on population viability analyses are typically lower than those assigned based on rule-based criteria [36]. Mismatches may be due to large effects of parameter uncertainty (compounded in modelled estimates of the probability of collapse compared to other measures of risk), or lower likelihood of complete collapse compared to extensive functional degradation for a large, interconnected reef. We found higher sensitivity to collapse thresholds for projections of future spatial distribution and probability of collapse than for functional degradation (electronic supplementary material, figure S18), implying that some RLE criteria are more prone to uncertainty than others.

The RLE requires assessors to define ecosystem-specific indicators of functional declines, rather than prescribed or generic indicators (e.g. species richness [4]). Fruitful selection of indicators demands a rigorous diagnostic process to identify cause–effect chains that influence ecosystem dynamics. Diagrammatic conceptual models (figure 2) are a simple device to support this diagnostic process, which is not only pivotal in structuring a risk assessment, but also valuable in designing management strategies to mitigate threats and monitor progress towards management goals [4]. We devised a checklist to select indicators, but this process was lengthy due to the limited number of existing coral reef assessments and the number of indicators produced by the ecosystem model. In practice, indicators were selected where the information base was sufficient to identify collapse thresholds and to support inference about changes over the three assessment time frames. In the MAR, collapse thresholds were more readily identifiable for biotic indicators, whereas data were more readily available for environmental indicators, reflecting trade-offs in relevance and measurability between biotic and environmental indicators [37]. In particular, biotic indicators represented ecosystem trajectories towards collapse more directly, whereas environmental indicators represented threats and were therefore less direct indicators of risk.

Ecosystem models can aid in bridging data gaps, corroborating assessments of functional declines, and selecting sensitive indicators. Our historical reconstruction over the period 1966–2015 showed large declines in piscivorous fish biomass mirroring declines in coral cover. Independently derived estimates of the same indicators can help increase confidence in the robustness of RLE assessment outcomes, with the relative severity of past declines in coral cover (approx. 64%) corroborated by both modelled and empirical data. Our future projections of functional declines revealed differential responses among trophic groups: coral cover showed a binary response to mass bleaching levels, while functional declines for herbivorous and piscivorous fish biomasses were less variable among threats (table 3 and figure 3). Herbivorous fish biomass was an insensitive indicator of the probability of ecosystem collapse (table 4), suggesting dampening or compensatory effects of threats in this middle trophic level. Coral cover and piscivorous fish biomass revealed complementary information on the impacts of multiple threats, and we recommend these two indicators for future RLE assessments of coral reefs.

Independent assessments of multiple indicators through rule-based criteria do not take into account interacting threats [12], making the ecosystem model invaluable for identifying interactions. Although the frequency of hurricanes in the MAR decreased over the last 50 years, the historical reconstruction revealed that compounding effects of mass bleaching and disease resulted in severely reduced coral cover during hurricane years (electronic supplementary material, figure S17). Mass bleaching was the primary driver of collapse in the MAR, with high levels of mass bleaching leading to assessments of Critically Endangered based on future declines in coral cover regardless of the levels of other threats (figure 3). We found the highest probabilities of collapse when ocean acidification and hurricane severity were also high, implying that mitigation of climate change and ocean acidification is key to securing the MAR in coming decades. The ecosystem model suggested that, in the absence of concurrent stressors, the effects of pollution on reef biota may be limited, but improved understanding of the impacts of sedimentation and nutrification under refined policy scenarios are needed to adequately estimate risks from pollution. Similarly, future incidence of coral disease was not included due to uncertainty, although scenarios of mass bleaching based on sea surface temperature account to some extent for coral susceptibility to disease [38].

We used state-of-the-art model validation techniques, by assessing steady-state model behaviour under various initial conditions, as well as quantifying model performance against empirical data with multiple performance metrics. Yet models are necessarily simplifications of a system with a number of assumptions that may affect the outcome of risk assessments. For example, our regional ‘whole-of-ecosystem’ model omitted fine-scale patterns in fish diversity and fishing, instead aggregating fish into functional groups and using fish biomass as an indicator [8], thus possibly underestimating risks posed by fishing. Parrotfish protection is predicted to have modest effects on maintaining coral cover in the Belize Barrier Reef by 2030 [39], supporting our main findings. Our model was calibrated on high estimates of coral growth rates including *Acropora* species, so our model may have over-estimated coral cover. Given the assessment of the ecosystem as Critically Endangered based on future declines in coral cover, a lower growth rate parametrization would not affect assessment outcomes. Our model did not incorporate three-dimensional reef structure, which can drive changes in fish communities and reef resilience to climate change [27]. Reef flattening is an on-going process in the Caribbean, so declines in reef structural complexity and fish diversity may have been under-estimated by our model. Finally, we assumed that the distribution of the MAR could not extend beyond mapped grid cells, despite evidence that corals could extend to more polar latitudes under climate change. Given that the ecosystem is listed as Least Concern based on future changes in spatial distribution, possible range extensions would not affect this result.

Our study of the MAR provides a clear example of how the RLE protocol estimates overall risk levels by assessing multiple threats and symptoms of decline. Risk assessment protocols and ecosystem models are thus able to integrate limited observational data with threat scenarios, making them valuable tools for monitoring ecosystem status and diagnosing key threats to be addressed by management. Our case study provides a template for assessing risks to coral reefs and for the further application of ecosystem models in risk assessment. Increases in availability of ecosystem models in terrestrial, marine and freshwater systems worldwide [4,8] render models not only useful, but increasingly accessible for supporting ecosystem risk assessment and ecosystem management.

## Supplementary Material

Supplementary Methods & Results

## Supplementary Material

Empirical data for biotic indicators

## Data accessibility

Details of the analysis can be found in electronic supplementary material, appendix S1. Empirical data on biotic indicators are available in electronic supplementary material, appendix S2. R scripts for analysis are available on GitHub (https://github.com/LucieBland/coral-reefs).

## References

[1] Cardinale BJ et al. 2012 Biodiversity loss and its impact on humanity. Nature 486, 59–67. (10.1038/nature11148) 22678280

[2] Keith DA et al. 2013 Scientific foundations for an IUCN red list of ecosystems. PLoS ONE 8, e62111 (10.1371/journal.pone.0062111) 23667454 PMC3648534

[3] Nicholson E, Keith DA, Wilcove DS 2009 Assessing the threat status of ecological communities. Conserv. Biol. 23, 259–274. (10.1111/j.1523-1739.2008.01158.x) 19245533

[4] Bland LM, Keith DA, Miller RM, Murray NJ, Rodríguez JP. 2016 Guidelines for the application of IUCN Red List of Ecosystems categories and criteria, version 1.0. Gland, Switzerland: IUCN.

[5] Sutherland WJ 2006 Predicting the ecological consequences of environmental change: a review of the methods. J. Appl. Ecol. 43, 599–616. (10.1111/j.1365-2664.2006.01182.x)

[6] Olsen E, Fay G, Gaichas S, Gamble R, Lucey S, Link JS 2016 Ecosystem model skill assessment. Yes We Can! PLoS ONE 11, e0146467 (10.1371/journal.pone.0146467) 26731540 PMC4701724

[7] Weijerman M, Fulton EA, Janssen ABG, Kuiper JJ, Leemans R, Robson BJ, van de Leemput IA, Mooij WM 2016 How models can support ecosystem-based management of coral reefs. Prog. Oceanogr. 138, 559–570. (10.1016/j.pocean.2014.12.017)

[8] Fulton EA 2010 Approaches to end-to-end ecosystem models. J. Mar. Syst. 81, 171–183. (10.1016/j.jmarsys.2009.12.012)

[9] Burns EL, Lindenmayer DB, Stein J, Blanchard W, McBurney L, Blair D, Banks SC 2015 Ecosystem assessment of mountain ash forest in the Central Highlands of Victoria, south-eastern Australia. Austral Ecol. 40, 386–399. (10.1111/aec.12200)

[10] Chen P-Y, Chen C-C, Chu L, McCarl B 2015 Evaluating the economic damage of climate change on global coral reefs. Glob. Environ. Change 30, 12–20. (10.1016/j.gloenvcha.2014.10.011)

[11] Kwiatkowski L, Cox P, Halloran PR, Mumby PJ, Wiltshire AJ 2015 Coral bleaching under unconventional scenarios of climate warming and ocean acidification. Nat. Clim. Change 5, 777–781. (10.1038/nclimate2655)

[12] Melbourne-Thomas J, Johnson CR, Fung T, Seymour RM, Chérubin LM, Arias-González JE, Fulton EA 2011 Regional-scale scenario modeling for coral reefs: a decision support tool to inform management of a complex system. Ecol. Appl. 21, 1380–1398. (10.1890/09-1564.1) 21774437

[13] McField M, Kramer P 2007 Healthy reefs for healthy people: a guide to indicators of reef health and social well-being in the Mesoamerican Reef Region. With contributions by M Gorrez and M McPherson. Miami, FL: Healthy Reefs for Healthy People Initiative.

[14] Melbourne-Thomas J, Johnson CR, Fulton EA 2011 Regional-scale scenario analysis for the Meso-American Reef system: modelling coral reef futures under multiple stressors. Ecol. Modell. 222, 1756–1770. (10.1016/j.ecolmodel.2011.03.008).

[15] Melbourne-Thomas J, Johnson CR, Fulton E 2011 Characterizing sensitivity and uncertainty in a multiscale model of a complex coral reef system. Ecol. Modell. 222, 3320–3334. (10.1016/j.ecolmodel.2011.07.014)

[16] Healthy Reefs for Healthy People Initiative. 2015 *MesoAmerican reef: a report card of ecosystem health*. Miami, FL: Healthy Reefs for Healthy People Initiative.

[17] Jackson J, Donovan M, Cramer K, Lam V 2014 *Status and trends of Caribbean coral reefs: 1970–2012*. Belize City, Belize: Global Coral Reef Monitoring Network.

[18] Burke L, Sugg Z 2006 *Hydrologic modeling of watersheds discharging adjacent to the Mesoamerican reef*. Washington, DC: World Resources Institute.

[19] Sheppard C, Rioja-Nieto R 2005 Sea surface temperature 1871–2099 in 38 cells in the Caribbean region. Mar. Environ. Res. 60, 389–396. (10.1016/j.marenvres.2004.12.006) 15769506

[20] Eakin CM et al. 2010 Caribbean corals in crisis: record thermal stress, bleaching, and mortality in 2005. PLoS ONE 5, e13969 (10.1371/journal.pone.0013969) 21125021 PMC2981599

[21] Friedrich T et al. 2012 Detecting regional anthropogenic trends in ocean acidification against natural variability. Nat. Clim. Change 2, 167–171. (10.1038/nclimate1372)

[22] Chan N, Connolly SR 2013 Sensitivity of coral calcification to ocean acidification: a meta-analysis. Glob. Change Biol. 19, 282–290. (10.1111/gcb.12011) 23504739

[23] Knutson TR et al. 2013 Dynamical downscaling projections of twenty-first-century Atlantic hurricane activity: CMIP3 and CMIP5 model-based scenarios. J. Clim. 26, 6591–6617. (10.1175/JCLI-D-12-00539.1).

[24] Knutson TR et al. 2010 Tropical cyclones and climate change. Nat. Geosci. 3, 157–163. (10.1038/ngeo779)

[25] Donner SD 2009 Coping with commitment: projected thermal stress on coral reefs under different future scenarios. PLoS ONE 4, e5712 (10.1371/journal.pone.0005712) 19492060 PMC2686172

[26] Abramson D, Bethwaite B, Enticott C, Garic S, Peachey T 2011 Parameter exploration in science and engineering using many-task computing. IEEE Trans. Parallel Distrib. Syst. 22, 960–973. (10.1109/TPDS.2010.177)

[27] Rogers A, Blanchard Julia L., Mumby Peter J 2014 Vulnerability of coral reef fisheries to a loss of structural complexity. Curr. Biol. 24, 1000–1005. (10.1016/j.cub.2014.03.026) 24746794

[28] Murray NJ, Keith DA, Bland LM, Nicholson E, Regan TJ, Rodríguez JP, Bedward M 2017 The use of range size to assess risks to biodiversity from stochastic threats. Divers. Distrib. 23, 474–483. (10.1111/ddi.12533)

[29] IMARS. 2004 Millenium Coral Reef Mapping Project. St Petersburg, FL: University of South Florida I.f.M.R.S.

[30] Sheppard CR 2003 Predicted recurrences of mass coral mortality in the Indian Ocean. Nature 425, 294–297. (10.1038/nature01987) 13679917

[31] Guinotte J, Buddemeier R, Kleypas J 2003 Future coral reef habitat marginality: temporal and spatial effects of climate change in the Pacific basin. Coral Reefs 22, 551–558. (10.1007/s00338-003-0331-4)

[32] Knapp KR, Applequist S, Diamond HJ, Kossin JP, Kruk M, Schreck C 2010 NCDC International Best Track Archive for Climate Stewardship (IBTrACS) project, version 3. See https://data.noaa.gov/dataset/ncdc-international-best-track-archive-for-climate-stewardship-ibtracs-project-version-3.

[33] Gardner TA, Cote IM, Gill JA, Grant A, Watkinson AR 2005 Hurricanes and caribbean coral reefs: impacts, recovery patterns, and role in long-term decline. Ecology 86, 174–184. (10.1890/04-0141)

[34] Ainsworth TD, Heron SF, Ortiz JC, Mumby PJ, Grech A, Ogawa D, Eakin CM, Leggat W 2016 Climate change disables coral bleaching protection on the great barrier reef. Science 352, 338–342. (10.1126/science.aac7125). 27081069

[35] IUCN Red List of Ecosystems. 2016 *IUCN Red List of Ecosystems Assessments*. Gland, Switzerland: IUCN.

[36] Gärdenfors U 2000 Population viability analysis in the classification of threatened species: problems and potentials. Ecol. Bull. 48, 181–190.

[37] Shin Y-J et al. 2010 Using indicators for evaluating, comparing, and communicating the ecological status of exploited marine ecosystems. 2. Setting the scene. ICES J. Mar. Sci. 67, 692–716. (10.1093/icesjms/fsp294)

[38] Maynard J et al. 2015 Projections of climate conditions that increase coral disease susceptibility and pathogen abundance and virulence. Nat. Clim. Change 5, 688–694. (10.1038/nclimate2625)

[39] Mumby PJ, Wolff NH, Bozec YM, Chollett I, Halloran P 2014 Operationalizing the resilience of coral reefs in an era of climate change. Conserv. Lett. 7, 176–187. (10.1111/conl.12047)

