# Supplementary material for: Using multiple lines of evidence to assess the risk of ecosystem collapse
Source: Proc Biol Sci. 2017 Sep 20;284(1863):20170660. doi: 10.1098/rspb.2017.0660 (PMC5627190; doi:10.1098/rspb.2017.0660)
Supplement: Supplementary Methods & Results [file rspb20170660supp1.docx]

**Appendix S1. Using multiple lines of evidence to assess the risk of ecosystem collapse**

[**Introduction to the IUCN Red List of Ecosystems** 3](#_Toc485214662)

[**Ecosystem description** 5](#_Toc485214663)

[Classification 5](#_Toc485214664)

[Distribution 5](#_Toc485214665)

[Characteristic native biota 6](#_Toc485214666)

[Abiotic environment 6](#_Toc485214667)

[Interactions and Processes 6](#_Toc485214668)

[Threats 7](#_Toc485214669)

[Ecosystem collapse 7](#_Toc485214670)

[**Criterion B** 9](#_Toc485214671)

[**Checklist for identifying biotic and environmental indicators** 10](#_Toc485214672)

[**Criterion C** 13](#_Toc485214673)

[Mass bleaching 13](#_Toc485214674)

[Ocean acidification 16](#_Toc485214675)

[Hurricanes 17](#_Toc485214676)

[Pollution 19](#_Toc485214677)

[**Criterion D** 21](#_Toc485214678)

[Coral cover 21](#_Toc485214679)

[Algal cover 24](#_Toc485214680)

[Herbivorous fish biomass 26](#_Toc485214681)

[Piscivorous fish biomass 28](#_Toc485214682)

[Urchin biomass and density 30](#_Toc485214683)

[Elkhorn coral and staghorn coral presence 31](#_Toc485214684)

[**Ecosystem model** 32](#_Toc485214685)

[Model components and local-scale dynamics 32](#_Toc485214686)

[Initial reef scenario 34](#_Toc485214687)

[*Setting forcings* 34](#_Toc485214688)

[*Using historical data to set initial values* 35](#_Toc485214689)

[*Using data from sites in good condition to set initial values* 36](#_Toc485214690)

[*Sensitivity to coral growth rates* 36](#_Toc485214691)

[*Results* 38](#_Toc485214692)

[Historical reconstruction 41](#_Toc485214693)

[*Setting forcings* 41](#_Toc485214694)

[*Assessing model skill* 43](#_Toc485214695)

[*Applying subcriterion D1* 47](#_Toc485214696)

[Future estimates of risk 49](#_Toc485214697)

[*Setting forcings and initial values* 49](#_Toc485214698)

[*Assessing subcriterion A2a* 51](#_Toc485214699)

[*Assessing subcriterion D2a* 52](#_Toc485214700)

[*Assessing criterion E* 54](#_Toc485214701)

[**References** 57](#_Toc485214702)

# **Introduction to the IUCN Red List of Ecosystems**

The IUCN Red List of Ecosystems is the new global standard for assessing risks to ecosystems. Here we provide a brief overview of the assessment process. Further information can be found in the IUCN Red List of Ecosystems Guidelines (Bland, Keith et al. 2016) and the Introduction to the IUCN Red List of Ecosystems (IUCN 2016). The IUCN Red List of Ecosystems protocol assesses ecosystems in eight categories of risk largely based on the IUCN Red List of Threatened Species™: Collapsed (CO), Critically Endangered (CR), Endangered (EN), Vulnerable (VU), Near Threatened (NT), Least Concern (LC), Data Deficient (DD), and Not Evaluated (NE, Figure S1). The Categories Data Deficient and NE do not reflect a level of risk, but indicate whether a lack of adequate data precludes assessment (Data Deficient), or assessment has not been attempted (Not Evaluated). The categories Critically Endangered, Endangered or Vulnerable indicate threatened ecosystems and are defined by quantitative criteria.


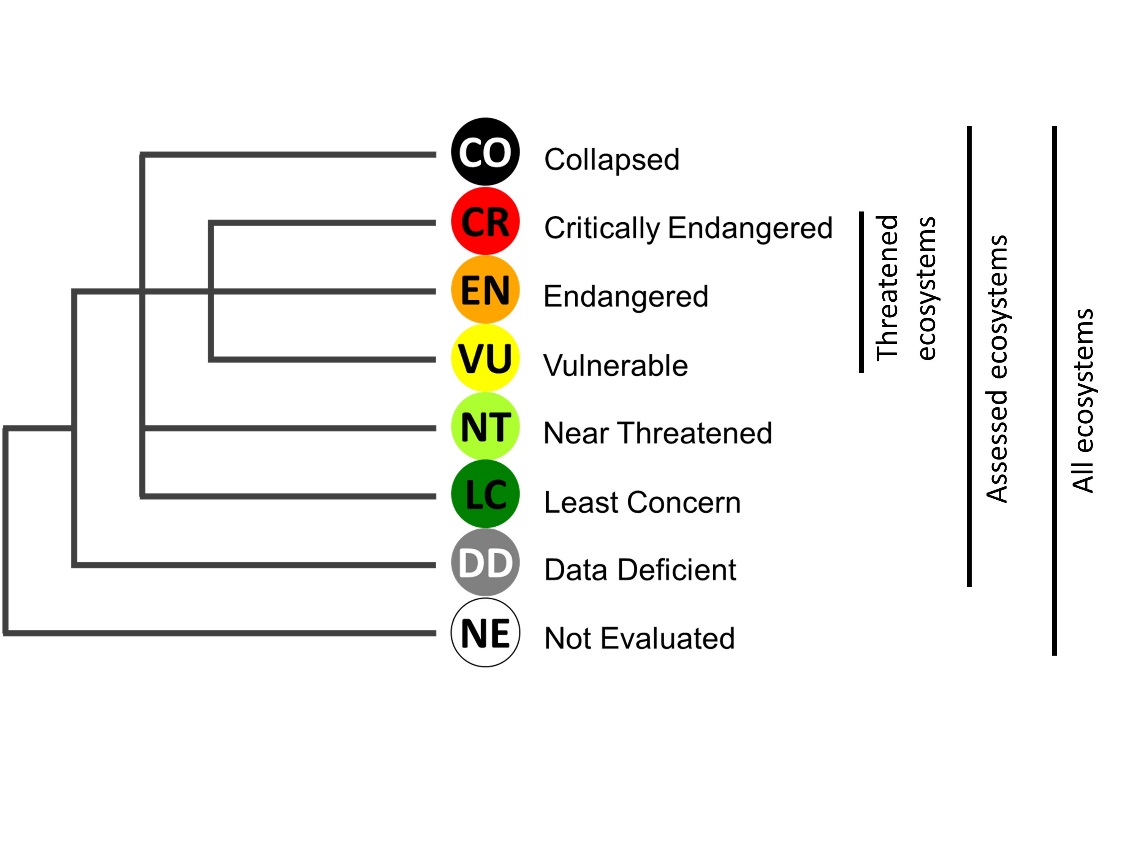


**Figure S1.** Categories of the IUCN Red List of Ecosystems, adapted from (Bland, Keith et al. 2016).

The IUCN Red List of Ecosystems uses five rule-based criteria to assign ecosystem to categories of risk. Criteria are designed to capture various symptoms of ecosystem degradation and trajectories towards collapse. Two criteria assess spatial symptoms of ecosystem collapse: declines in spatial distribution (criterion A) and small distribution size (criterion B). Two criteria assess functional symptoms, including environmental degradation (criterion C) and biotic disruption (criterion D). Declines in spatial distribution, environmental degradation, and biotic disruption are measured over three timeframes: the past 50 years (subcriterion 1); the next 50 years (subcriterion 2a); and since the pre-industrial period (subcriterion 3). Finally, criterion E evaluates quantitative estimates of the risk of collapse over the next 50–100 years.

Categories of risk are delimited by thresholds that vary among criteria and time frames (Figure S2) e.g. future declines in ecosystem distribution or function of at least 30 %, 50 %, or 80 %, respectively correspond to the Vulnerable, Endangered, and Critically Endangered categories. Declines since the pre-industrial period are assessed over a longer period of time, so thresholds in ecosystem distribution or function are more severe (i.e. 50 % for the Vulnerable category, 70 % for the Endangered category, and 90 % for the Critically Endangered category). Criterion B uses different thresholds related to the geographic distribution size of the ecosystem, and the number of locations at which the ecosystem is present. Criterion B uses thresholds in the predicted probability of collapse of the ecosystem over the next 50 to 100 years to assess ecosystems as threatened (i.e. Vulnerable. ≥ 10% within 100 years; Endangered: ≥ 20% within 50 years; Critically Endangered: 50% within 50 years). If adequate data to assess a criterion are lacking, this criterion is assessed as Data Deficient. The highest category of risk obtained by any of the criteria determines the overall level of risk of the ecosystem.

IUCN Red List of Ecosystems assessments must be repeatable and transparent, and can be based on a variety of data sources that must be adequately reported. To assess risks to the Meso-American Reef, we followed the standard IUCN assessment process (Figure S2).


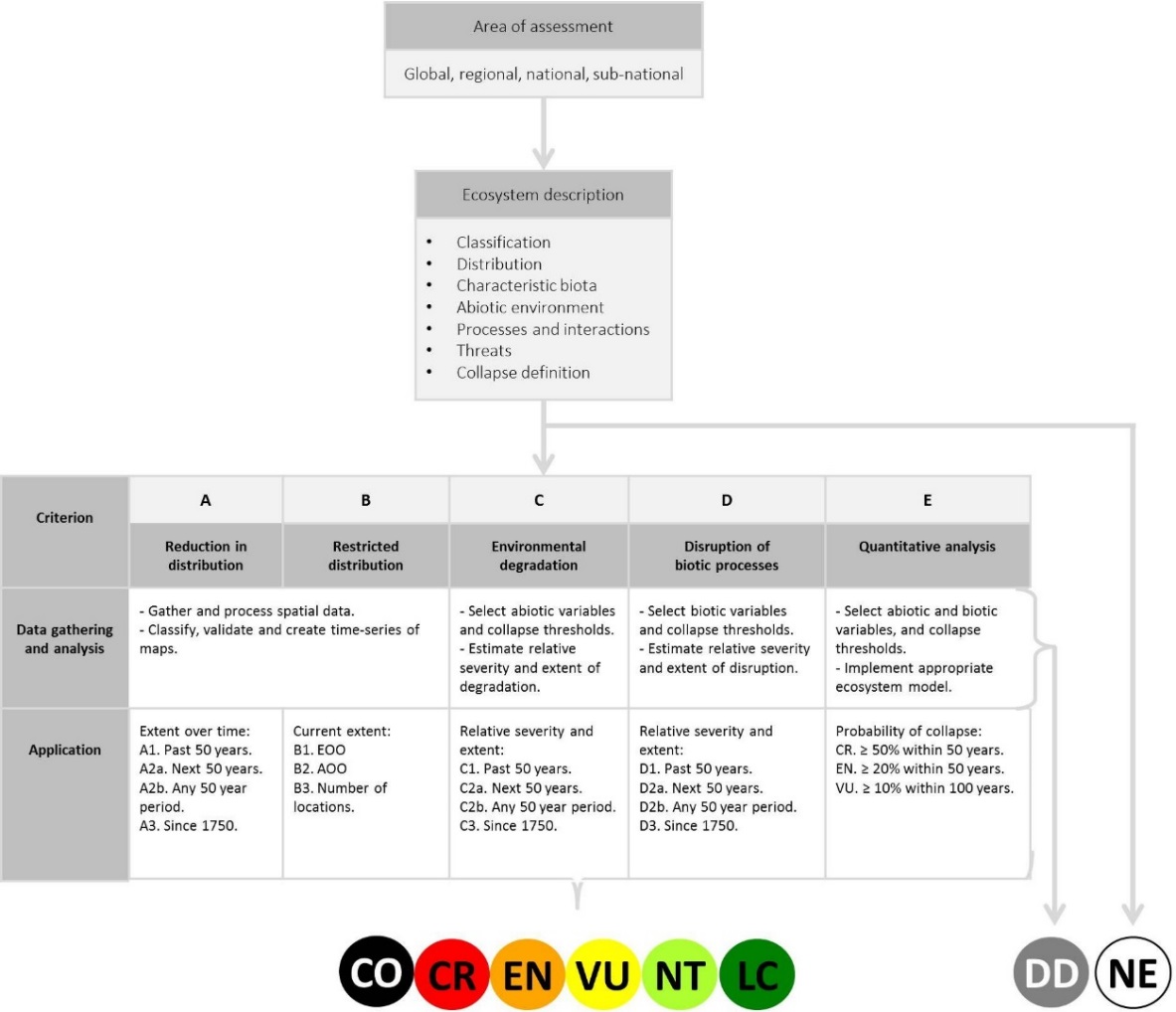


**Figure S2.** Process for assessing the risk of collapse of an ecosystem, adapted from (Bland, Keith et al. 2016).

# **Ecosystem description**

## Classification

The Meso-American Reef (MAR) is classified as habitat type 9.8 Coral Reef under the IUCN Habitats Classification, and is part of the Western Caribbean Marine Ecoregion of the World (Spalding, Fox et al. 2007).

## Distribution

The MAR is located between 88.86 and 85.70 °W longitude, and 15.83 and 21.56 °N latitude. The MAR extends for 1,000 km along the coastline of four countries: Mexico, Belize, Guatemala, and Honduras. The MAR contains the second longest barrier reef in the world (Belize) and four offshore atolls, along other diverse reef structures (e.g. lagoonal patch reefs, fringing reefs) (Garcia-Salgado, Nava-Martinez et al. 2008). Reef locations in the MAR were derived from the Millennium Coral Reef Mapping Project’s geomorphological classification (from 30 m Landsat imagery; IMARS 2004) and only include classifications with a high probability of being living coral (forereef, reef flat, barrier reef pinnacle, and shallow terrace) (Figure S3). The distribution size of the MAR is 1,359 km^2^ (in the approximate water depth range 5-20 m). We defined five subregions within the MAR, following Melbourne-Thomas et al. (2011): Northern Quintana Roo; Southern Quintana Roo; Chinchorro Bank; Belize and Guatemala; and Honduras (Figure S3).


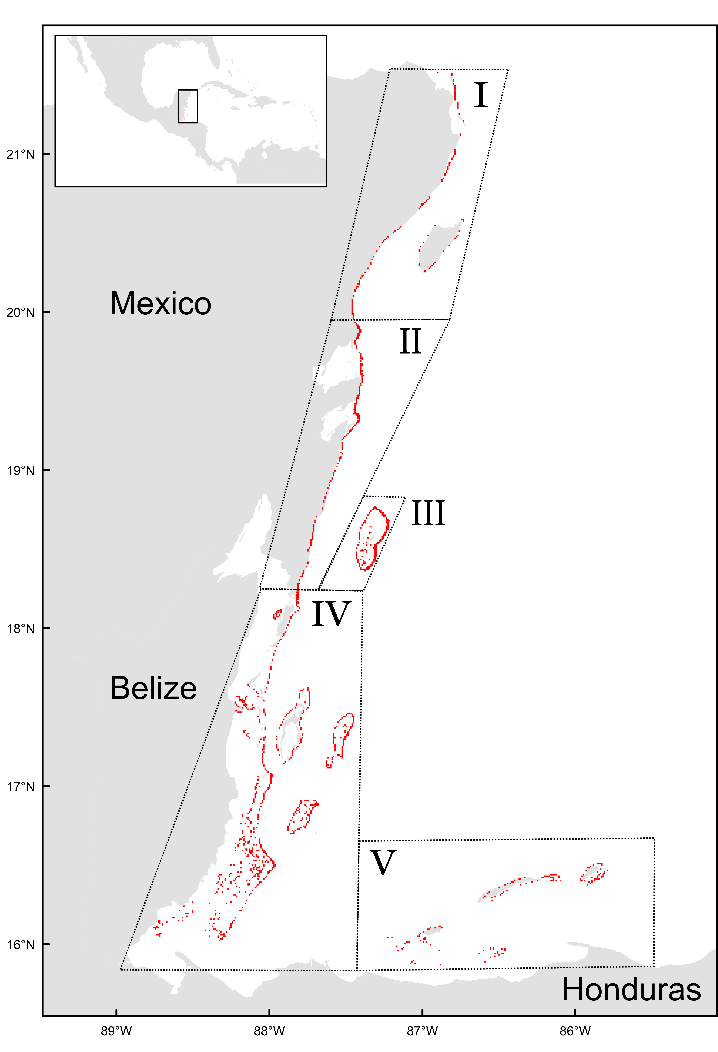


**Figure S3.** Distribution of the Meso-American Reef. Red squares indicate reef occurrences, with red squares indicating cells occupied by reef at a 1 km^2^ resolution. I: Northern Quintana Roo; II: Southern Quintana Roo; III: Chinchorro Bank; IV: Belize and Guatemala; V: Honduras.

## Characteristic native biota

The MAR contains 67 species of reef-building corals (McField and Kramer 2007), many of which are endemic to the larger Caribbean region. Widespread hermatypic genera include *Acropora*, *Montastrea*, *Porites, Agaricia, Diploria, Colpophylia, Meandrina, Mycetophyllia, Dendrogyra,* as well as non-Scleractinian fire corals *Millepora* spp. Over the last 20 years, once-dominant reef-building corals have been replaced by structurally simpler species, such as *Porites asteroides* (McField and Kramer 2007). A study conducted in 12 sites in Belize in 1997 reported more than 30 species per 225-m^2^ site, and a Shannon-Wiener diversity index of 1.8 (McField 2000). The MAR has an abundance of octocorals and sponges that grow from the hard coral base. Other prominent invertebrate groups include crustacean, mollusca and holothuria (including the sea urchin *Diadema antillarum*).

Diversity of reef fish in the MAR is similar to other reefs in the Caribbean (McField and Kramer 2007). At least 245 species of reef fish are found along the Yucatan Peninsula, 317 in Belize, 218 in Guatemala, and 294 in Honduras (McField and Kramer 2007). Abundant families include parrotfish (Scaridae), grunt (Haemulidae), snapper (Lutjanidae), grouper (Serranidae), angelfish (Pomacanthidae), butterflyfish (Chaedontidae), damselfish (Pomacentridae), jawfish (*Opistognanthus* spp.), wrasse (Labridae), and surgeonfish (Acanthuridae). The numbers of recorded families (48-54) are similar throughout the MAR (McField and Kramer 2007). Marine turtles (*Chelonia mydas*, *Dermochelys coriacea*, *Eretmochelys imbricata*) are still found in the MAR, but have probably lost their role as keystone species due to overexploitation (McClenachan, Jackson et al. 2006).

## Abiotic environment

The MAR environment is characterized by warm, shallow waters (rarely > 60 m in depth) in clear, relatively nutrient-poor and open coastal waters. Sea temperature varies between 25 and 29 °C, salinity ranges from 34 to 37 ppt, and pH ranges from 7.8 to 8.6 (McField and Kramer 2007).

## Interactions and Processes

The MAR average of coral recruits is lower than the Caribbean average (3 *vs.* 4.5 recruits per m^2^) (McField and Kramer 2007). In the MAR, reef tracts for which recruitment is low are more vulnerable to natural disturbance and anthropogenic threats (Melbourne-Thomas, Johnson et al. 2011). Reefs in Mexico and northern Belize receive fish larvae from Cuba, although there is high inter-annual variability in dispersal due to weather and oceanographic patterns (Cowen, Paris et al. 2006). Habitat fragmentation and destruction of mangrove nurseries can limit recruitment and reduce genetic diversity (McField and Kramer 2007). As in other Caribbean reefs, important processes include competition for space among benthic groups and herbivory (Mumby 2006, Ferrari, Gonzalez-Rivero et al. 2012, Adam, Burkepile et al. 2015)(Figure 2).

## Threats

The main threats to the MAR are coral disease, bleaching, overfishing, pollution (sedimentation and nutrification), direct damage, and global climate change (McField and Kramer 2007). Historically, the MAR has experienced a number of threats in close succession. A disease outbreak in 1983 wiped out 98 % of the long-spined sea urchin population (*Diadema antillarum*) (Lessios 1988), leading to algal-dominated states on reefs where overfishing had depleted herbivorous fish populations (Garcia-Salgado, Nava-Martinez et al. 2008). Corals are also vulnerable to diseases such as white band, black band, and white pox diseases. Major hurricanes hit the MAR in 1998 (Mitch), 2000 (Keith), and 2001 (Iris). Mass bleaching events occurred in 1995, 1998, and 2005 due to elevated sea surface temperature. In 1998 alone, the combined effects of bleaching, hurricanes, and background stress led to the loss of 50 % coral cover (Garcia-Salgado, Nava-Martinez et al. 2008). Bleaching events in 1995 and 2005 did not cause significant mortality (McField, Bood et al. 2008).

Increased sea surface temperature and ocean acidification are two of the most pressing global threats affecting coral reefs (Hoegh-Guldberg, Mumby et al. 2007). The tropical oceans have warmed by 0.40 °C between the periods 1871-1900 and 1979-2008, about 70 % of the global average value. Declines in sea water pH and aragonite saturation hinder coral calcification (Doney, Fabry et al. 2009) and increase bioerosion and carbonate dissolution (Andersson and Gledhill 2013).

Local and regional threats include declines in water quality due to increased sediment, nutrient, and chemical pollution from changes in coastal land use (Hughes, Baird et al. 2003). Sedimentation and nutrification are a threat to the MAR, particularly for reefs in Honduras and Guatemala (Burke and Sugg 2006). Hurricanes can directly decimate reef structures, as well as reduce salinity through heavy rainfall (Lough 2011). Given time (~10–20 years) and no other sources of environmental stress, coral reefs recover naturally from hurricanes (Lough 2011). However, high frequency and intensity hurricanes can negatively affect reefs and synergistically interact with other threats. The MAR has also suffered from intense fishing activity since the 1960s (Garcia-Salgado, Nava-Martinez et al. 2008).

## Ecosystem collapse

To estimate risk, it is necessary to define the endpoint of ecosystem decline i.e. the point at which an ecosystem is considered collapsed (Bland, Keith et al. 2016). Within the IUCN Red List of Ecosystems, “an ecosystem is Collapsed when it is virtually certain that its defining biotic or abiotic features are lost from all occurrences, and the characteristic native biota are no longer sustained” (Bland, Keith et al. 2016).

We considered the MAR to be collapsed when live coral cover declined to < 1 % throughout the mapped ecosystem distribution. As corals are the main structural elements of the reef, coral cover is the main variable suitable for detecting ecosystem collapse (De’ath, Fabricius et al. 2012). We assume that collapse occurs when live coral cover declines to 0-1 % throughout the distribution. We used a more conservative threshold compared to the 5 % threshold used in the Caribbean RLE assessment (Appendix S2; Keith, Rodríguez et al. 2013), as the Healthy Reef Initiative defines 5 % coral cover as ‘critical’ (Healthy Reefs Initiative 2015), a concept more similar to the Critically Endangered than the Collapsed category. As average coral cover across the Caribbean is low (13.6%)(Jackson, Donovan et al. 2014), we did not want to overestimate the number of locally collapsed coral patches in the MAR. We defined collapse thresholds for environmental indicators based on required levels to reach a coral cover < 1%.

We defined separate collapse thresholds for herbivorous fish and piscivorous fish biomass, two informative indicators of reef state (see criterion D)(McField and Kramer 2007). We defined collapse thresholds for herbivorous and piscivorous fish biomass as 5 g/m^2^ and 2 g/m^2^ respectively (Appendix S1). We devised these collapse thresholds by maintaining a consistent risk classification compared to the Reef Health Index categories (Healthy Reefs Initiative 2015) across all three biotic indicators (coral cover, herbivorous fish biomass, piscivorous fish biomass)(see criterion D). We selected collapse thresholds to ensure that thresholds of the Critically Endangered category would correspond to the “critical” or “poor” categories of the Reef Health Index. Overall, the IUCN Red List of Ecosystems thresholds were more precautionary than the Reef Health Index thresholds, because the Reef Health Index does not included a Collapsed or analogous category.

# **Criterion B**

A location is defined as a geographically- or ecologically-distinct area in which a single threat can rapidly affect occurrences of the ecosystem type (Bland, Keith et al. 2016). Numbers of locations must be defined for each significant threat in the ecosystem likely to cause collapse over a short time period (~ 20 years). Relevant threats for the MAR include pollution, fishing, hurricanes, bleaching, and ocean acidification. None of these threats are likely to cause the ecosystem to become Critically Endangered within 20 years (see criteria C and E). Hence the status of the ecosystem is Least Concern under subcriterion B3. However, we apply the concept of location to the MAR for future reference and guidance on applying the IUCN RLE Categories and Criteria to coral reefs.

The number of independent locations subject to pollution is at least three, as sedimentation plumes generated in Honduras and Guatemala are known to affect Belizean but not Mexican reefs (Paris and Chérubin 2008). Northern Quintana Roo, Southern Quintana Roo, and the rest of the ecosystem (Belize, Honduras, and Guatemala) therefore constitute three locations. The number of locations with fishing is four, assuming that different national jurisdictions implement different quotas and regulations on fishing. Most hurricanes affect two MAR subregions and only one hurricane has affected three subregions since 1852 (Knapp, Applequist et al. 2010). Because a single hurricane is unlikely to cause total mortality in all reefs within one subregion, we assume that there are five distinct threat-defined locations in the MAR. These interpretations of the concept of location are broad; in reality the response of reefs to threats will differ based on local factors so the number of locations may be higher.

# **Checklist for identifying biotic and environmental indicators**

Criteria C and D entail an assessment of functional declines over three time periods: the past 50 years (C1 and D1); the next 50 years (C2a and D2a) or any time period including the present and future (C2b and D2b); and since the industrial exploitation period (C3 and D3). There is a vast quantity of literature identifying ecosystem indicators (Niemeijer and de Groot 2008, Boldt, Martone et al. 2014). To select indicators relevant for risk assessment, we devised a simple checklist consisting of five steps (Table S1). This allowed us to assess the relevance of different ecosystem processes; compare indicators of the same process; justify transformations and calculations; and assess uncertainty in estimates of functional declines.

1. **Relevance to ecosystem function**. There must be plausible evidence of a causal relationship between the process of environmental degradation or biotic disruption, and the loss of characteristic native biota (Bland, Keith et al. 2016). We identified which abiotic and biotic factors were most related to ecosystem function with the conceptual model (Figure 2). We identified direct and indirect links; the sign and direction of those links; and alternative indicators for the same process. Variables with direct cause-effect relationships and the greatest sensitivity to loss of characteristic native biota are the most suitable for assessment under criteria C and D (Bland, Keith et al. 2016).
2. **Data availability and quality**. Assessing functional declines requires suitable scalar variables for estimating the severity of decline, as well as suitable spatial variables for estimating the extent of decline. To assess criteria C and D, data should ideally be available over a 50 year timeframe at different locations within the ecosystem. Although assessments can be completed with few data points, interpolation and extrapolation within the 50 year timeframe should be adequately justified. We assessed:

*a. Reliability of data sources.* This included an assessment as to how the data were collated and whether there were known biases and uncertainties in the data source.

*b. Time frame and temporal resolution of the time series.*

*c. Spatial extent and spatial resolution of the time series.*

1. **Selection of the initial value**. Relative severity of decline is calculated based on an initial value (C1 and D1: 50 years ago; C2a and D2a: present or recent past value; C3 and D3: industrial development period). Estimates of relative severity are likely to be sensitive to uncertainty in the initial value. This uncertainty is linked to the quality of the data (step 2) and methods of interpolation and extrapolation. We attempted to collect data for these 50 year time frames, but used interpolation and extrapolation of time series as well as backcasting with the ecosystem model to fit those time frames (e.g. subcriterion D1).
2. **Selection of a threshold for collapse**. The choice of a variable should enable direct inferences about threshold values of the variables that define ecosystem collapse (Bland, Keith et al. 2016). If the variable is irrelevant to ecosystem function (step 1) it may be very difficult to select a threshold for ecosystem collapse. In some ecosystems, selecting a collapse threshold for criterion D is the basis for risk assessment as it denotes the loss of characteristic native biota. Assessors should strive to define congruent thresholds for ecosystem collapse for criteria C and D. For example, a threshold for collapse for sedimentation (criterion C) should correspond to the complete loss of characteristic native biota (e.g. no live coral cover; criterion D). This may be difficult to establish in the absence of empirical evidence or when the value of the abiotic variable after the ecosystem has collapsed cannot be inferred. We offer detailed explanations for the selection of collapse thresholds, in particular for biotic indicators where we compare IUCN Red List of Ecosystems collapse thresholds with the Healthy Reef Index (Healthy Reefs Initiative 2015).
3. **Shape of decline**. Variables can be transformed to obtain a relevant indicator of progress towards ecosystem collapse. For example, raw sea surface temperature data can be transformed to degree heating weeks, degree heating months, or temperature anomalies (Van Oppen and Lough 2008). Transformation of the variable relies on an assumption about the shape of decline leading to the loss of characteristic native biota. Each incremental increase in an abiotic variable measured in criterion C may lead to a small or large loss of characteristic native biota, depending on the specific value of that variable. This shape of decline can be understood as a classic exposure-response relationship (Suter 2006), where the loss of characteristic native biota may accelerate or decelerate at different points of the exposure-response curve. In the absence of detailed data on shapes of decline, a linear relationship is often assumed (Bland, Keith et al. 2016). We assumed linear relationships between (transformed) abiotic variables and progress towards collapse in criterion C. This assumption may be inaccurate in complex systems like coral reefs, which often show non-linear changes and hysteretic behaviour (Karr, Fujita et al. 2014). As a result, assessments under criterion C may over- or under-estimate risk if non-linear behaviours are ignored. Further studies could use the ecosystem model to assess the effects of assumptions on shapes of declines on the modelling results. In line with the IUCN Red List of Ecosystems Guidelines (Bland, Keith et al. 2016), assumptions concerning shapes of decline must be clearly reported.

**Table S1.** Checklist for selecting indicators to assess criteria C, D, and E.

|  | Step | Explanation | Example |
| --- | --- | --- | --- |
| 1 | Relevance to ecosystem function | - Identify relevant processes with the conceptual model  - Refine understanding of alternative indicators  - Assess the strength of cause-effect relationship and sensitivity of indicator to the loss of characteristic native biota | - Increase in sea surface temperature is a key threat to coral reefs due to mass bleaching.  - Warming can be quantified with different variables, such as temperature anomalies, degree heating weeks, and degree heating months (DHM).  - Sea warming is directly linked to the loss of characteristic native biota (live coral cover) through coral mortality, decrease in reproductive output and growth (Van Oppen and Lough 2008). |
| 2 | Data availability and quality | - Describe data sources  - Describe time frame and temporal resolution of the time series  - Describe spatial extent and spatial resolution of the time series | - Data on sea surface temperature are available in a published dataset (Sheppard and Rioja-Nieto 2005).  - Data are available for the period 1852-2099 every month.  - The dataset covers the whole MAR region with three grid cells of 2.5 x 3.75 degree resolution. |
| 3 | Selection of collapse threshold | - Select the threshold denoting ecosystem collapse for that variable  - If relevant, describe alternative thresholds | - We used the probability of bleaching (*p* = 0.2) calculated over running 10-year intervals as the threshold for ecosystem collapse (Sheppard 2003). |
| 4 | Selection of initial and present/future values | - Describe the selection of the initial and present/future values given data quality (step 2)  - If relevant, describe alternative values | - We used the monthly SST data to calculate DHMs (expressed as °C·month) for the period 1871-2099.  - We averaged DHMs over the period 1917-1965 to obtain a long-term average.  - Initial values were similar for all three locations. |
| 5 | Shape of decline | - Describe the shape of the relationship between the loss of characteristic biota and the indicator | - We assume a linear relationship between increase in probability of severe bleaching and progression towards ecosystem collapse. |

# **Criterion C**

## Mass bleaching

**1.** **Relevance to ecosystem function.** Coral bleaching is a stress response where corals expel their dinoflagellate symbionts, which can lead to high mortality and reduction in coral cover. Rise in Sea Surface Temperature (SST) has been linked to coral bleaching globally and in the Caribbean, especially during the mass bleaching events of 1998 and 2005 (McField, Bood et al. 2008, Jackson, Donovan et al. 2014). SST accurately predicts bleaching events and the NOAA Coral Reef Watch programme uses satellite-derived SSTs to predict coral bleaching in real-time (Liu, Strong et al. 2006).

**2. Data availability and quality.** Risk of bleaching can be measured with a range of variables such as raw SST, SST anomalies compared to a baseline (McWilliams, Côté et al. 2005), and Degree Heating Weeks (Liu, Strong et al. 2006). Degree Heating Months (DHM) constitute an approximation of Degree Heating Weeks (DHW), the variable measured by NOAA’s Coral Reef Watch to predict coral bleaching in real time (Liu, Strong et al. 2006). The accumulation of DHW (1 week of SST greater than the maximum in the monthly climatology) over a rolling 12-week period is an effective indicator of the likelihood of bleaching (Donner, Skirving et al. 2005). Coral bleaching begins to occur when DHW > 4, and severe bleaching occurs when DHW > 8. Annual maximum DHMs (equal to one month of SST that is 1°C greater than the maximum in the monthly mean climatology) correlate well with annual maximum DHWs in the Caribbean (Donner, Knutson et al. 2007). DHM is a more appropriate variable than DHW for risk assessment as climate predictions with Global Climate Models are averaged monthly rather than weekly.

Blended SST data are available since 1871 on HadISST (Rayner, Parker et al. 2003) and are projected to 2099 with the HadCM3 model (Gordon, Cooper et al. 2000) at a resolution of one month (Sheppard and Rioja-Nieto 2005). The data cover the entire MAR region, although some coastal areas of Belize, Guatemala, and Honduras are excluded (Sheppard and Rioja-Nieto 2005). Spatial resolution for past and future SST is fairly coarse (2.5 x 3.75 ° grid cells) due to constraints in backcasting and forecasting climate data. Data are available for three MAR grid cells: Cancun, Cozumel, and Yucatan. The relative area of each location (Cancun, Cozumel, Yucatan) was 12.5%, 37.5%, and 50% of the MAR respectively. We weighted the relative severities for each location by their area to obtain relative severity for the whole MAR (Table S3).

More complex analyses of bleaching risk based on different climate scenarios are available (Donner, Knutson et al. 2007, Donner 2009), but are beyond the scope of this study. Data at 5-km resolution are available from 1985 to the present (Casey, Brandon et al. 2010) and could be used to assess subcriterion C1, although the length of this time series is less suitable (30 years).

**3. Selection of collapse threshold.** Corals can recover from mass bleaching events if intervals between events are sufficiently long (> 5 years; Sheppard 2003). We used the probability of bleaching (*p* = 0.2) calculated over running 10-year intervals as the threshold for ecosystem collapse (Sheppard 2003).

**4. Selection of initial and present/future values.** We used monthly SST data (Sheppard and Rioja-Nieto 2005) to calculate DHMs (expressed as °C·month) for the period 1871-2099, as outlined in Donner et al. (2005, 2007). We calculated the maximum of the mean monthly temperatures over the years 1985-2005. The warmest month in the climatology was October for Cozumel and Yucatan, and August for Cancun. For the whole period, we found those months which exceeded the maximum monthly climatology by more than 1°C and calculated the accumulation of DHM over a 4-month rolling window. A maximum DHM accumulation over the year of more than 2 °C·months is correlated with severe bleaching (Donner 2009). We derived the years in which severe bleaching occurred, and computed the probability of severe bleaching over a 10-year running interval to match our threshold for ecosystem collapse.

The response of corals to temperature stress is variable within and among reefs (Hughes, Baird et al. 2003), indicating that some corals may be capable of adapting to thermal stress by shifting to symbioses with more resistant species of *Symbiodinium.* We followed Donner et al. (2007) and investigated the impact of thermal adaptation by corals by increasing the temperature at which thermal stress accumulates by 1 °C (Table S2, Figure S4). We recorded the maximum probability of mass bleaching under the two scenarios (no coral adaptation and coral adaptation) for the periods 1966-2015 (C1), 2016-2065 (C2a), and 1876-2015 (C3), and compared those with minimum probabilities to calculate relative severities. This implies that the maximum severity could occur at any time during the time period.

**5. Shape of decline.** We assume a linear relationship between increase in probability of mass bleaching and progression towards ecosystem collapse.





**Figure S4.** Probability of annual Degree Heating Months (DHM) exceeding 2°C·month for three Meso-American Reef areas mapped in (Sheppard and Rioja-Nieto 2005), assuming: a) no coral adaptation; and b) coral adaptation to thermal stress by 1°C. The annual probability is calculated over a 10-year running interval. The threshold of collapse (*p* = 0.2) corresponds to severe bleaching events occurring more than once every five years.

**Table S2.** Relative severity of bleaching for three grid cells within the Meso-American Reef (MAR). Relative severity is calculated with the probability of Degree Heating Month >2 °C·month. Relative severity is expressed on a scale from 0 (no change) to 1 (collapse). Grid cells were defined based on the sea surface temperature dataset (Sheppard and Rioja-Nieto 2005).The overall value for the Meso-American reef is a weighted average of the relative severities observed at the three grid cells. The coral adaptation scenario assumes an increase of thermal tolerance of corals by 1 °C.

| Subcriterion | Scenario | Cancun | Cozumel | Yucatan | MAR | Category |
| --- | --- | --- | --- | --- | --- | --- |
| C1 | No coral adaptation | 0 | 0 | 1 | 0.5 | EN |
| C2a | No coral adaptation | 1 | 1 | 1 | 1 | CR |
| C2a | Coral adaptation | 0 | 0.5 | 0.5 | 0.44 | VU |
| C3 | No coral adaptation | 0 | 0 | 1 | 0.5 | VU |

## Ocean acidification

**1. Relevance to ecosystem function.** Due to their three-dimensional structure, corals provide habitat and shelter for many reef species. Declines in sea water pH and aragonite saturation reduce coral growth rates, disrupt metabolic processes, increase bioerosion and lead to framework dissolution (Doney, Fabry et al. 2009, Andersson and Gledhill 2013). Several environmental variables influence net carbonate budgets, such as light, water turbidity, wave exposure, temperature and bioerosion (Carricart-Ganivet, Cabanillas-Teran et al. 2012, Chan and Connolly 2013, Kennedy, Perry et al. 2013), but we use sea surface aragonite saturation as the main indicator of reef net carbonate budget. More complex carbonate budget modelling methods are available to account for the influence of other factors on reef net carbonate budgets (Kennedy, Perry et al. 2013).

**2. Data availability and quality.** Aragonite saturation has been projected back to pre-industrial times and forward to the year 2100 at an annual time-step, resulting in a detectable downward trend since 1850 (Friedrich, Timmermann et al. 2012). To estimate declines in aragonite saturation over the last 50 years, we used modelled data presented in Friedrich et al. (2012) as observed data are only available since 1988 (Gledhill, Wanninkhof et al. 2008). The spatial resolution of the data is low as the dataset covers the whole Caribbean. It could be possible to downscale spatially-explicit projections of aragonite saturation to the region of interest (Cao and Caldeira 2008, Foden, Butchart et al. 2013), but this is beyond the scope of our study.

**3. Selection of collapse threshold.** The aragonite saturation states (Ω_arag_) of 3.5 (Cao and Caldeira 2008) and 3.25 (Hoegh-Guldberg, Mumby et al. 2007) have been proposed as thresholds below which almost no reefs currently occur. Experimental studies have suggested that coral calcification reaches zero when Ω_arag_ = 1 (Langdon and Atkinson 2005), but other studies have found that corals may continue to calcify below the aragonite saturation threshold or may not calcify at much higher thresholds (Pandolfi, Connolly et al. 2011, Chan and Connolly 2013). We use an intermediate threshold of Ω_arag_ = 3, an environment defined as “extremely marginal” (Guinotte, Buddemeier et al. 2003) and used in previous vulnerability assessments (Foden, Butchart et al. 2013).

**4. Selection of initial and current/future values.** We extracted values for 1966, 2015, 2065, and the pre-industrial average from the dataset (Friedrich, Timmermann et al. 2012) and calculated relative severities (Table S3).

**5. Shape of decline.** Experimental studies have suggested that coral calcification declines linearly with declining aragonite saturation (Langdon and Atkinson 2005), a finding confirmed by a recent meta-analysis (Chan and Connolly 2013).

**Table S3.** Application of criterion C with modelled aragonite saturation (Ω_arag_) in the Caribbean. Relative severity is calculated for the entire extent of the ecosystem. For each subcriterion, a collapse threshold of 3.0 was applied.

| Subcriterion | Initial value | Current/future value | Relative severity | Category |
| --- | --- | --- | --- | --- |
| C1 | 4.8 | 4.4 | 0.22 | LC |
| C2a | 4.4 | 3.7 | 0.5 | EN |
| C3 | 5 | 4.4 | 0.3 | LC |

## Hurricanes

**1. Relevance to ecosystem function.** Hurricanes (here defined as tropical cyclones with speed >33 m/s; Nyberg, Malmgren et al. 2007) can directly decimate reef structures through the large waves they generate, as well as temporarily reduce salinity through heavy rainfall and cause coastal destruction associated with storm surges (Lough 2011). Given time (~10-20 years) and no other sources of environmental stress, coral reefs can recover from such local physical disturbances (Lough 2011). However, high frequency and intensity of hurricanes could negatively affect reefs and synergistically interact with other threats.

**2. Data availability and quality.** Records depict high variability in hurricane frequency in the North Atlantic over the distant (last 1,500 years; Mann, Woodruff et al. 2009) and recent past (last 270 years; Nyberg, Malmgren et al. 2007). Ship track information is available from the 1840s onwards, aerial reconnaissance has been operational since 1944, and satellite surveillance has operated since 1966 (Knutson, McBride et al. 2010). These methods can assess, with varying degrees of accuracy, the track (path), speed (intensity), and number of days of the hurricane. However, observational biases are present in ship track and aerial reconnaissance datasets: number of hurricanes are underestimated by ship tracks (Vecchi and Knutson 2008), and aerial reconnaissance is less effective in detecting cyclones lasting less than two days (Landsea, Vecchi et al. 2010). It is however possible to apply subcriterion C1 with unbiased satellite data from 1966 onwards. Applying subcriterion C2a is more problematic, due to the difficulties in predicting future climates and variables relevant to hurricane formation, as well as creating simulation models of hurricanes (Knutson, McBride et al. 2010). Despite these difficulties, hurricane frequency and intensity in the North Atlantic has been predicted with a variety of climate models, climate downscaling methods, and cyclone simulation methods (Knutson, McBride et al. 2010, Emanuel 2013, Knutson, Sirutis et al. 2013).

**3. Selection of collapse threshold.** There is no evidence of recovery to a pre-hurricane state for at least eight years after impact of hurricanes categories 1 to 5 in the Caribbean (Gardner, Cote et al. 2005), so we defined the collapse threshold as a hurricane frequency of one in eight years. There is no information on the time to recovery from hurricanes categories 4 and 5, but reefs experience a higher immediate decrease in coral cover following higher category hurricanes (Gardner, Cote et al. 2005), so we assume that recovery from hurricanes categories 4 and 5 will be on average slower than for hurricanes categories 1 to 5. We define the collapse threshold for hurricanes categories 4 and 5 as one in twelve years. Uncertainty in the threshold for recovery from strong hurricanes affects estimates of relative severity, so determining recovery times from strong hurricanes in the Caribbean should be of high research priority.

**4. Selection of initial and present/future values.** The MAR is composed of five subregions, and all but one hurricane since 1853 have affected either one or two of those subregions (Knapp, Applequist et al. 2010). Because a single hurricane is unlikely to cause total mortality in all reefs within one subregion, we assume that there are five distinct threat-defined locations in the MAR. We divided the hurricane frequency information by the number of subregions to account for independence among subregions. We calculated average annual probabilities of occurrence of hurricanes for the periods 1944-1965 and 1966-2012. We used these two timeframes to only use aerial reconnaissance data (post-1944) and satellite data (post-1966) rather than ship track data (pre-1944), and to conform to the 50 year time frame of subcriterion C1. Modelling studies consistently project decreases in the global frequency of tropical cyclones by 6-34 % in the 21st century. Future increase in hurricane frequency is therefore unlikely to be an important threat to coral reefs, so we do not assess overall frequency of hurricanes categories 1 to 5 under subcriterion C2a. However, modelling studies predict an increased frequency of strong hurricanes (categories 4 and 5), with large variations among studies (Knutson, McBride et al. 2010). A recent study using the most recent Coupled Model Intercomparison Project (CMIP5) indicates a marginally significant increase (+45 % and +39 %) for the early and late 21^st^ century, respectively (Knutson, Sirutis et al. 2013). Our analysis reveals a 59 % relative severity in the increase of frequency of hurricanes categories 1 to 5 between the periods 1853-1903 and 1966-2012. However, the dataset suffers from an underestimation of the number of hurricanes pre-aerial reconnaissance (1944). We therefore assess subcriterion C3 based on an analysis accounting for biases in hurricane records which indicates no significant trends in the North Atlantic hurricane frequency since the mid-1800s (Knutson, McBride et al. 2010), hence a relative severity of 0 % (Table S4). We find the overall frequency is lower than the threshold for collapse (one in 16 years) and the number of hurricanes in the dataset is unlikely to have been underestimated by a half. We are therefore confident the ecosystem did not meet the threshold for collapse in either the 1853-1903 or 1966-2012 periods. The ecosystem is therefore listed as Least Concern under subcriterion C3.

**5. Shape of decline.** We assume that progression towards ecosystem collapse scales linearly with hurricane frequency and hurricane intensity.

**Table S4.** Application of criterion C with hurricane data in the Meso-American Reef.

| Subcriterion | Collapse threshold | Hurricane categories | Relative severity | Category |
| --- | --- | --- | --- | --- |
| C1 | 0.125 | 1-5 | -0.12 | LC |
| C1 | 0.083 | 4-5 | 0.06 | LC |
| C2a | 0.083 | 4-5 | 0.33 | VU |
| C3 | 0.125 | 1-5 | 0 | LC |

## Pollution

**1. Relevance to ecosystem function.** Excess sediments and nutrients are recognized to as having negative effects on reef communities. The effects of sediments include burial of corals, bleaching, and colonization of the surface by filamentous bacteria (Risk and Edinger 2011). The three main effects of nutrients are release from nutrient limitation (e.g. nitrogen, phosphorus, and organic matter), light limitation from increased turbidity, and flow-on effects from altered sediment properties (Fabricius 2011). Because sediment and nutrient input often covary, corals are affected by two conflicting factors: increased sediment input slows growth whilst individual corals may grow faster under elevated nutrients. However, corals face more intense competition from algae and bioerosion can outstrip accretion rates (Risk and Edinger 2011).

**2. Data availability and quality.** No regional water quality database is available for the MAR. No long-term (> 2 years) routine monitoring data for nitrogen and phosphorus could be located (McField and Kramer 2007). Direct measurement of nutrients in the water is also problematic due to relatively low nutrient loads in coral reefs, and the rapid uptake of nutrients by phytoplankton. These issues do not only affect the MAR – lack of consistent methods for pollution research and the confounded effects of increased sediment input and increased nutrients have hampered the relevance of on-the-ground measurements for coral conservation (Risk and Edinger 2011).

Water transparency could be used as a proxy for sediment and nutrient concentrations. However sedimentation rate, salinity, and water transparency data do not have the temporal and spatial replication suitable for assessment of criterion C (McField and Kramer 2007). The Reefs at Risk Revisited report (2011) provides maps of the effect of coastal development (a proxy for nutrification) and watershed-based pollution (a proxy for sedimentation). However, the levels of the categories presented (low, medium, high) are not detailed enough to enable analysis under criterion C.

Pre-industrial, current and future sedimentation data are available for the hydrosheds bordering the MAR (Burke and Sugg 2006). Since industrial development, sediment loads delivered to the MAR have increased 22 times and nitrogen load three times (Burke and Sugg 2006). Depending on the future scenario considered, sediment loads are projected to change by -5 % to +13 % and nitrogen loads by -4 % to +8 % (Burke and Sugg 2006). Burke & Sugg (2006) showed that over 80 % of sediment and 50 % of nutrients originated in Honduras, whilst Guatemala was the source of 17 % of sediment and 25 % of nutrients, and Belize and Mexico contributed little to runoffs. Similarly, a century-scale geochemical record for the region (Carilli, Prouty et al. 2009) showed that most run-off originates from the south of the MAR, and that runoff has increased steadily since 1840.

Detailed analyses of river-reef connectivity based on modelling and remotely-sensed ocean colour (SeaWiFS) are available for the MAR (Chérubin, Kuchinke et al. 2008, Paris and Chérubin 2008). These studies reveal that most buoyant matter reaches the reefs from June to September following the peak time of river discharge. Reefs in the southwestern MAR are affected a second time with runoff of mixed origin due to as cyclonic gyre (Paris and Chérubin 2008). However, these data only offer a snapshot (1997-present) of the discharge affecting the region, and do not extend into the past or future. Cloud contamination remains a challenge for building temporally-resolved datasets with SeaWiFS images.

**3. Selection of collapse threshold.** Data from Burke & Sugg (2006) or Carilli et al. (2009) could be used to assess changed levels of runoff since pre-industrial times (subcriterion C3), but no threshold for collapse can be set for these time series. It is unclear what level of buoyant matter (modelled or observed with SeaWiFS) would lead to coral mortality and ecosystem collapse. It has been suggested that Caribbean reefs cannot survive under continued levels of suspended particulate matter greater than 10 mg/L (Risk and Edinger 2011), and that sedimentation rates should not be in excess of 10 mg/cm^2^/day (Rogers 1990). However in the absence of long-term on-the-ground measurements and the inability to relate the above values to coarse-scale simulation studies, we cannot apply criterion C.

# **Criterion D**

The Reef Health Index provides indicator levels that describe the condition of the MAR as very good, good, fair, poor, and critical (Healthy Reefs Initiative 2015). Reef Health Indices have been determined for coral cover, fleshy macroalgal cover, key herbivorous fish (parrotfish and surgeonfish), and key commercial fish (snappers and groupers). We provide a comparison of indicators for coral cover, macroalgal cover, and herbivorous fish for the Reef Health Index and the IUCN Red List of Ecosystems (RLE) categories.

## Coral cover

**1. Relevance to ecosystem function.** Change in live coral cover widely regarded as a critical measure of habitat loss and degradation, given the key role that corals play in creating the three-dimensional structure of the reef. Coral cover was used as an indicator for criterion D in the Caribbean coral reef assessment (Appendix S2 in Keith, Rodríguez et al. 2013). A “healthy” MAR can be characterized as having high live coral cover; moderate levels of crustose coralline, calcareous, and short turf algae; and low cover of fleshy macroalgae (McField and Kramer 2007).

**2. Data availability and quality.** Coral cover is one of the most commonly measured parameters in monitoring programmes. We obtained 27 coral cover records for the period 1986-2004 from Melbourne-Thomas et al. (2011), which we complemented with 28 records for the periods 1970-1985 and 2005-2013 (Appendix S2). For each record, we noted mean coral cover and, if available, the number of sites from which this record was derived, and the low and high range of coral cover among those sites (Figure S6). We recorded the subregion of origin of each record. Twenty-five records originated from the Belize and Guatemala subregion, compared to 30 for all other subregions combined.

**3. Selection of collapse threshold.** We assume that collapse occurs when live coral cover declines to 0-1 % throughout the distribution (Figure S5). We used a more conservative threshold than the 5 % threshold used in the Caribbean coral reef assessment (Appendix S2 in Keith, Rodríguez et al. 2013), as the Healthy Reef Initiative defines 5 % coral cover as “Critical” (Healthy Reefs Initiative 2015), a concept more similar to the Critically Endangered than the Collapsed category in this assessment. As average coral cover across the Caribbean is currently low (13.6 %) we did not want to over-estimate the number of collapsed coral reef occurrences in the MAR.

**4. Selection of initial and current/future values.** We used linear weighted regression to predict initial and current/future values to assess subcriterion D1 within the required time frame. We investigated polynomials up to third order and two groupings of subregions: all five subregions, and Belize and Guatemala versus all other subregions. We selected models with changes in AIC (ΔAIC; Burnham and Anderson 2002). Nearly half of records and all pre-1979 records originated from the Belize and Guatemala subregion, which is known to have naturally high coral cover (Jackson, Donovan et al. 2014). We were reluctant to use estimated historical trends from the Belize and Guatemala subregion to estimate 1970 values for all other subregions. Instead, we fitted two different models for these subregions, repeating the procedure outlined above. The best-fitting model for Belize and Guatemala was a second-order polynomial of year (AIC = 209.0; ΔAIC >2). Using this linear model, we estimated an initial coral cover (1970) of 74.6 % and a current coral cover (2015) of 20.8 %. We assumed the initial value in 1966 was equal to the value in 1970, as extrapolation of the model to 1966 produced unrealistic estimates of coral cover (88.8 %), and it is unlikely that substantial losses in coral cover occurred between 1966 and 1970. The relative severity of the decline over that subregion is 73.1 % assuming a collapse value of 1 %, and 72.1 % assuming a collapse value of 0 %. The best-fitting model for all other subregions was a second-order polynomial of year (AIC = 213.3; ΔAIC >2). Using this linear model, we estimated an initial coral cover (1970) of 34.1 %, and a current coral cover (2015) of 17.1 %. Similarly, we assumed the 1970 estimate was representative of live coral cover in 1966. The live coral cover in 1966 extrapolated by the model was 39.9 %. The relative severity of the decline over that subregion is 51.4 % assuming a collapse value of 1 %, and 49.9 % assuming a collapse value of 0 %. Based on the relative areas occupied by each subregion, the weighted average of relative severity over the entire ecosystem is 63.4 % (1 % threshold) or 62.2 % (0 % threshold). The ecosystem is therefore listed as Endangered under subcriterion D1.

**5. Shape of decline.** We assume that progression towards ecosystem collapse is linearly related to decrease in coral cover.


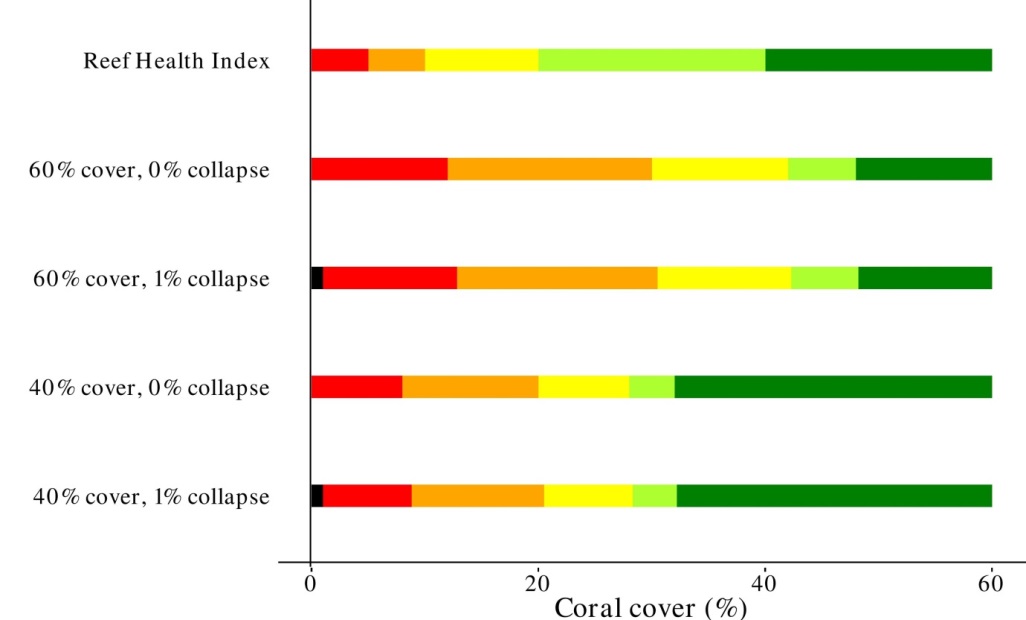


**Figure S5.** Levels of coral cover (%) corresponding to the categories of the Reef Health Index and Red List of Ecosystems categories. Reef Health Index: dark green: very good; light green: good; yellow: fair; orange: poor; and red: critical. Red List of Ecosystems: dark green: Least Concern; light green: Near Threatened; yellow: Vulnerable; orange: Endangered; red: Critically Endangered; black: Collapsed. A reef with 21 % coral cover would be classified as good with the Reef Health Index, but Vulnerable with the RLE categories based on 40 % initial coral cover and Endangered based on 60 % initial cover. There is more variation in the thresholds for RLE categories based on the selection of initial coral cover rather than collapse threshold.


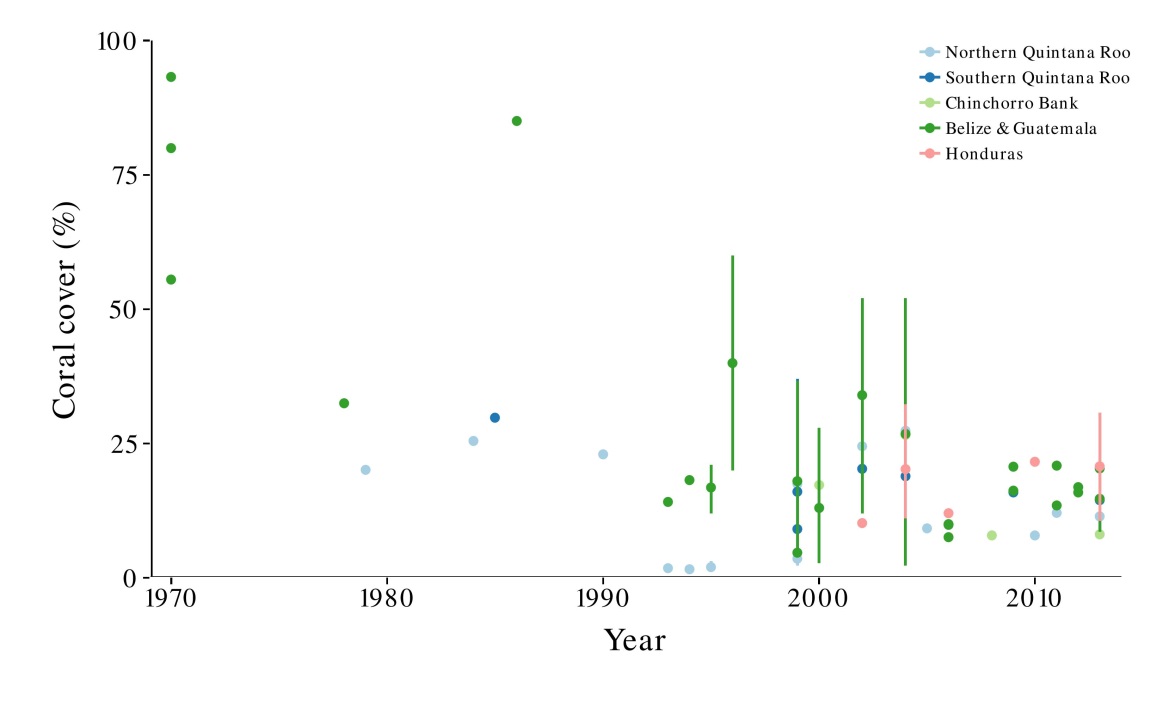


**Figure S6.** Coral cover in the Meso-American Reef between the years 1970-2013 in five subregions (n = 55). The bars indicate the low and high range for mean coral cover obtained from multiple sites.

## Algal cover

**1. Relevance to ecosystem function.** A shift from coral-dominated reefs to reefs dominated by turf and fleshy algae can lead to the loss of the reef framework (Kuffner, Walters et al. 2006, Box and Mumby 2007, Rasher and Hay 2010). Coral larvae avoid substrates where algae are present, and larval recruits suffer increased mortality and lower growth rates due to physical interference with algae (Jackson, Donovan et al. 2014). Algal cover in the MAR averages 25 % with higher amounts on fore reefs (27 %) than reefs crests (19 %) (McField and Kramer 2007). The MAR average is lower than the Caribbean value (34 %) (McField and Kramer 2007).

**2. Data availability and quality.** Algae data are difficult to collate due to methodological differences among studies recording different algal types (e.g. macroalgae and turf) (Jackson, Donovan et al. 2014). We collated data for algae, which we define as: i) macroalgae (green, red, and brown algae with larger thallus size and greater structural complexity than macroturf); and ii) macroturf (Melbourne-Thomas, Johnson et al. 2011). These two categories correspond to the “macroalgae” definition of McField et al. (2007). We obtained 19 records for the years 1993-2004 (Melbourne-Thomas, Johnson et al. 2011). We obtained 16 records for the years 1970-2012 from Jackson et al. (2014). Unfortunately these two datasets are not directly comparable, as Jackson et al. (Jackson, Donovan et al. 2014) do not define algal types present in their “macroalgae” category, but note that much of the monitoring data was discarded due to “monitoring and taxonomic inconsistencies”. We used the Jackson et al. (2014) dataset to estimate trends over the 43-year time period (Figure S8), and we used the Melbourne-Thomas et al. (2011) dataset to validate the ecosystem model. Coral cover to macroalgae ratio has been recommended as an indicator in the MAR (McField and Kramer 2007), but we did not collate data on this indicator due to the paucity of matching records on both cover types.

**3. Selection of a collapse threshold.** The Healthy Reefs Initiative considers an algal cover >25 % as “critical” (Healthy Reefs Initiative 2015). To determine collapse thresholds for macroalgae cover, we investigated differences among Reef Health Index and RLE thresholds (Figure S7). However, given the high prevalence of algae in the MAR and wider Caribbean, a 25 % algal cover may represent too stringent a threshold for ecosystem collapse (Figure S7). Given that substrate can be occupied by algae, coral, and epilithic algal communities, it is not clear what cover of algae would lead to the complete loss of the coral reef ecosystem (coral cover <1 %). The algal cover indicator is therefore less proximal to the process of ecosystem collapse than the coral cover indicator. We cannot derive a threshold for ecosystem collapse for this indicator.

**4. Selection of initial and current/future values.** We attempted to estimate a historical trend in macroalgal cover. We selected models with changes in AIC (ΔAIC; Burnham and Anderson 2002). Each value was weighted by the number of sites underlying that value, as raw data for each site were not available. We investigated polynomials up to third order. The best-fitting model was a second-order polynomial (AIC = 334.0; ΔAIC >2). The model indicated a positive trend in macroalgal cover in the MAR (linear coefficient = 31.7, standard error = 20.6; quadratic coefficient = -76.1; standard error = 21.7).

**5. Shape of decline.** We could not determine the shape of the relationship between macroalgal cover and progression towards ecosystem collapse.

**
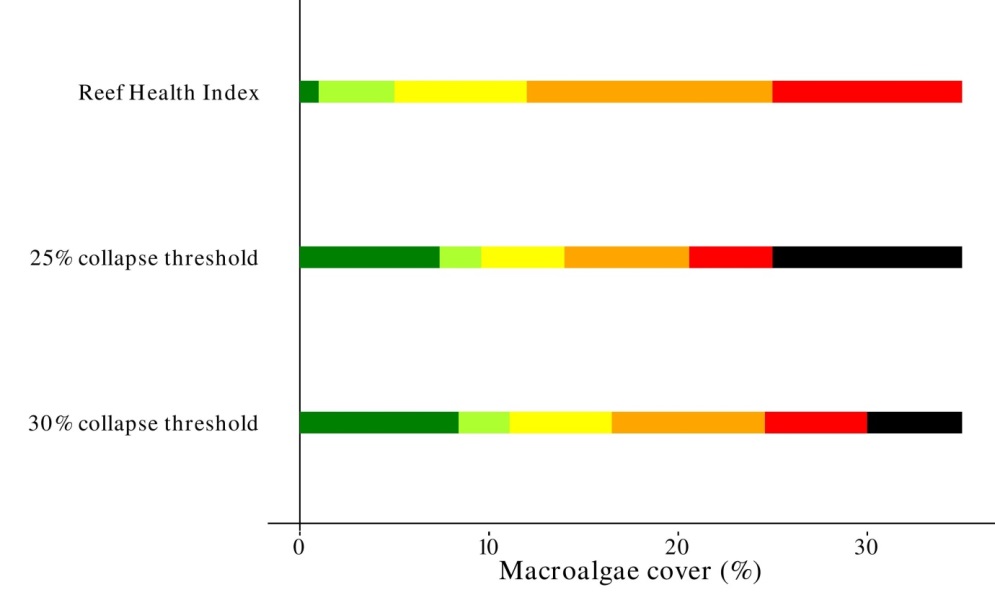
**

**Figure S7.** Levels of macroalgae cover (%) corresponding to the categories of the Reef Health Index and Red List of Ecosystems categories. Reef Health Index: dark green: very good; light green: good; yellow: fair; orange: poor; and red: critical. Red List of Ecosystems: dark green: Least Concern; light green: Near Threatened; yellow: Vulnerable; orange: Endangered; red: Critically Endangered; black: Collapsed.


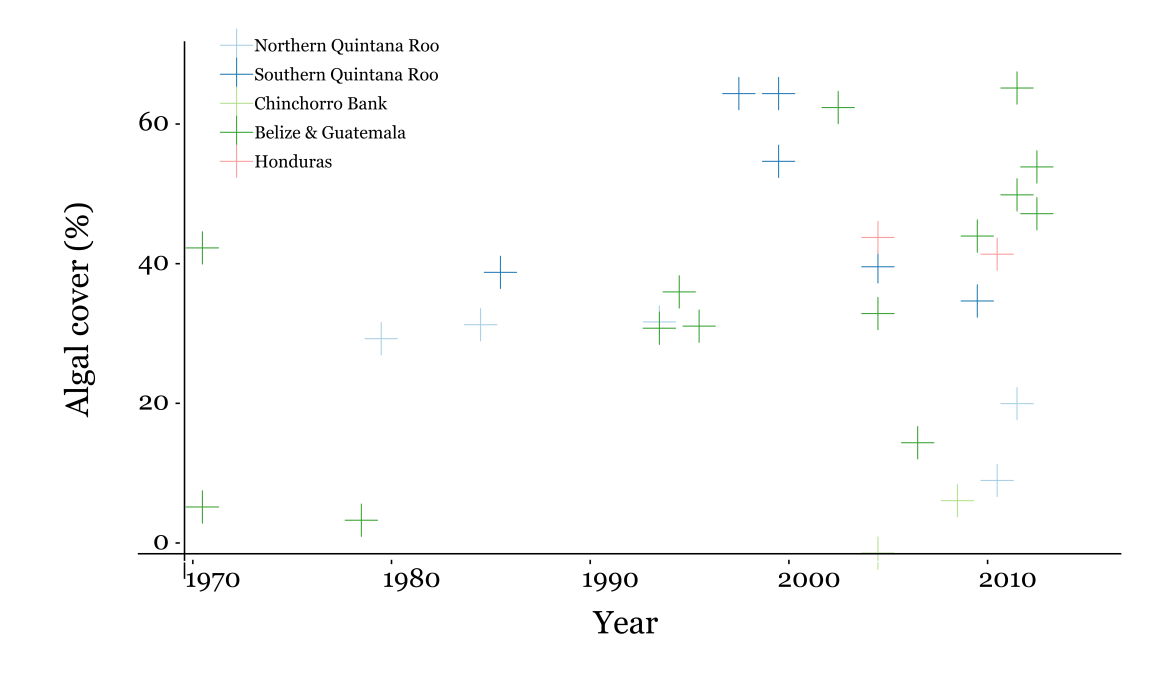


**Figure S8.** Algal cover in the Meso-American Reef between the years 1970-2013 in five subregions (n = 35).

## Herbivorous fish biomass

**1. Relevance to ecosystem function.** Herbivorous fish play a key role in coral reef ecosystems by grazing epilithic algal communities, macroturf and macroalgae. These include fish from the families Acanthuridae (surgeonfish) and Scaridae (parrotfish). Overfishing in reef communities has led to algal dominance through large areas of the Caribbean, especially Jamaica and the US Virgin Islands (Jackson, Donovan et al. 2014). Overfishing is strongly correlated with ecological collapse of reefs ecosystems, characterized by decrease in coral cover and recruitment, and increase in macroalgal abundance and coral disease (Jackson, Donovan et al. 2014). Historic declines in herbivorous fish in the Caribbean lead to dependence on urchins for control of algal communities, and may have been a precursor of changes in coral and algal communities when disease caused the collapse of urchin populations (Lessios 1988).

**2. Data availability and quality.** We collated 30 records of herbivore biomass for the period 1998-2013 from Melbourne-Thomas et al. (2011) and Healthy Reefs Initiative (2015) (Figure S10, Appendix S2). Although the time frame of this time series is not appropriate for assessing criterion D1, we attempted to estimate a historical trend in herbivore biomass. We selected models with changes in AIC (ΔAIC; Burnham and Anderson 2002). Each value was weighted by the number of sites underlying that value, as raw data for each site were not available. We investigated polynomials up to third order. The best-fitting models were the null model (AIC = 245.0) and the first-order polynomial (AIC = 245.8). The effect of year on herbivore biomass was not significant. Thus, there was no evidence for a change in herbivore biomass in the MAR over the time period under consideration.

**3. Selection of a collapse threshold.** Reef Health Index (RHI) considers herbivorous fish biomass <9.60 g/m^2^ as critical (Healthy Reefs Initiative 2015). We set two thresholds for collapse: herbivorous fish biomass of 0 g/m^2^ (extinction) and herbivorous fish biomass of 5 g/m^2^. We believe a threshold of 5 g/m^2^ is more appropriate because: i) for this measure, the collapse threshold should be higher than zero as the functional loss of herbivorous fish is likely to have very serious consequences for the maintenance of herbivory and coral-macroalgae dynamics in the MAR, especially at low urchin densities; ii) according to our comparison of RHI and RLE for corals, a RHI status of “critical” is likely to reflect a Red List status between Collapsed and Critically Endangered (Figure S9). In the absence of further data on a realistic threshold, the mid-point between total extinction (0) and the “critical” RHI (9.6) seems plausible.

**4. Selection of initial and current/future values.** Initial values for the 1960s and 1970s could not be computed due to the paucity of historical data.

**5. Shape of decline.** We assumed the shape of the relationship between herbivorous fish abundance and progression towards ecosystem collapse to be linear.


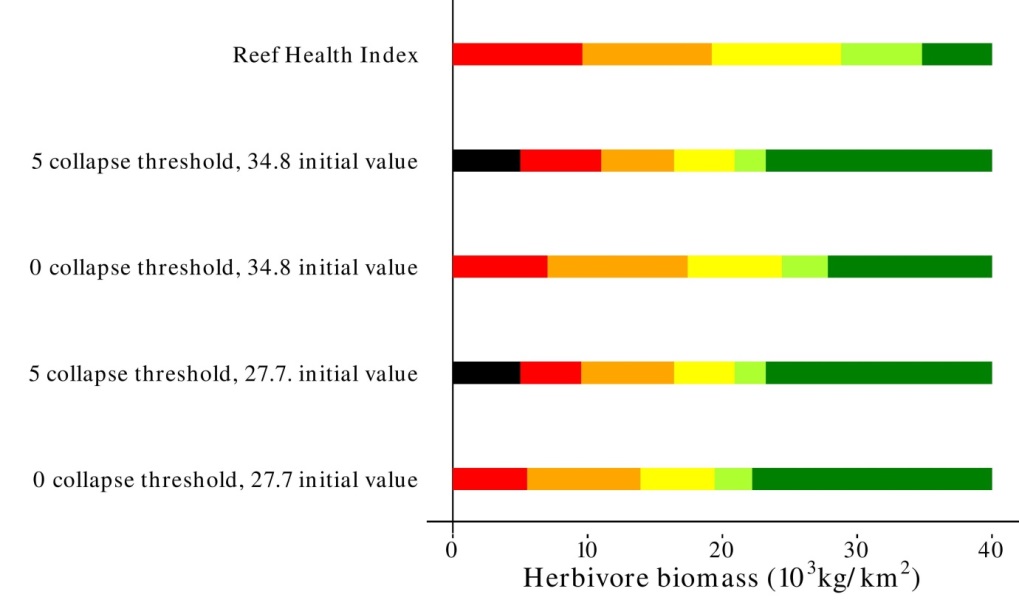


**Figure S9.** Levels of herbivore biomass (g/m^2^) corresponding to the categories of the Reef Health Index and Red List of Ecosystems categories. Reef Health Index: dark green: very good; light green: good; yellow: fair; orange: poor; and red: critical. Red List of Ecosystems: dark green: Least Concern; light green: Near Threatened; yellow: Vulnerable; orange: Endangered; red: Critically Endangered; black: Collapsed. For herbivorous fish biomass, there was more variation based on the collapse threshold than the initial cover selected.


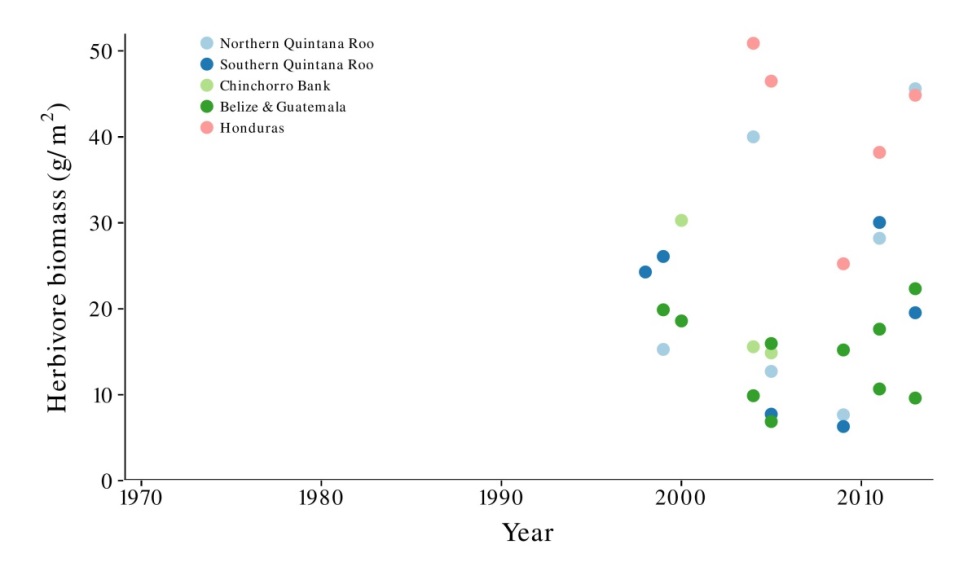


**Figure S10.** Herbivorous fish biomass in the Meso-American Reef between the years 1970-2013 in five subregions (n = 30).

## Piscivorous fish biomass

**1. Relevance to ecosystem function.** Piscivorous fish prey on herbivorous and small piscivorous fish, and can indicate the shape of the trophic food web on reefs (Weijerman, Fulton et al. 2015). Whilst piscivorous fish may be less relevant to the maintenance of the coral reef ecosystem than herbivorous fish, piscivorous fish (especially large ones) are significantly fished and are relevant to the provision of ecosystem services (McField and Kramer 2007).

**2. Data availability and quality.** We obtained 31 records of piscivorous fish biomass (g/m^2^) for the years 1998-2013 from Melbourne-Thomas et al. (2011) and Healthy Reefs Initiative (2015) (Figure S12, Appendix S2). The time frame of this time series is not appropriate for assessing criterion D1, but we attempted to estimate a historical trend in piscivore biomass. We selected models with ΔAIC (Burnham and Anderson 2002). Each value was weighted by the number of sites underlying that value, as raw data for each site were not available. We investigated polynomials up to third order. The best-fitting model was the first-order polynomial (AIC = 205.6; ΔAIC >2), indicating a significant decrease in piscivorous fish biomass over the period (coefficient: -9.88; standard error: 4.65).

**3. Selection of a collapse threshold.** A lower bound for ecosystem collapse could be of 0 g/m^2^, and we assumed an upper bound of 2 g/m^2^ based on comparisons with the Reef Health Index (Figure S11).

**4. Selection of initial and current/future values.** Initial values for the 1960s and 1970s could not be computed due to the paucity of historical data. Piscivorous fish biomass estimated with the linear model in 1998 was 11.4 g/m^2^, and 5.9 in 2013 g/m^2^. This indicates a relative severity of decline of 48.8 % over that time period, assuming a collapse threshold of 0 g/m^2^. Assuming a collapse threshold of 1 g/m^2^, the relative severity of decline was 53.4 % over that time period. The most likely status of the ecosystem is Endangered assuming that fish biomass in the 1960s were similar or higher than 1998 biomass. However, due to uncertainty in the data, we do not use this indicator to assess subcriterion D1.

**5. Shape of decline.** We did not determine the shape of the relationship between herbivorous fish abundance and progression towards ecosystem collapse, although it is likely to be linear.


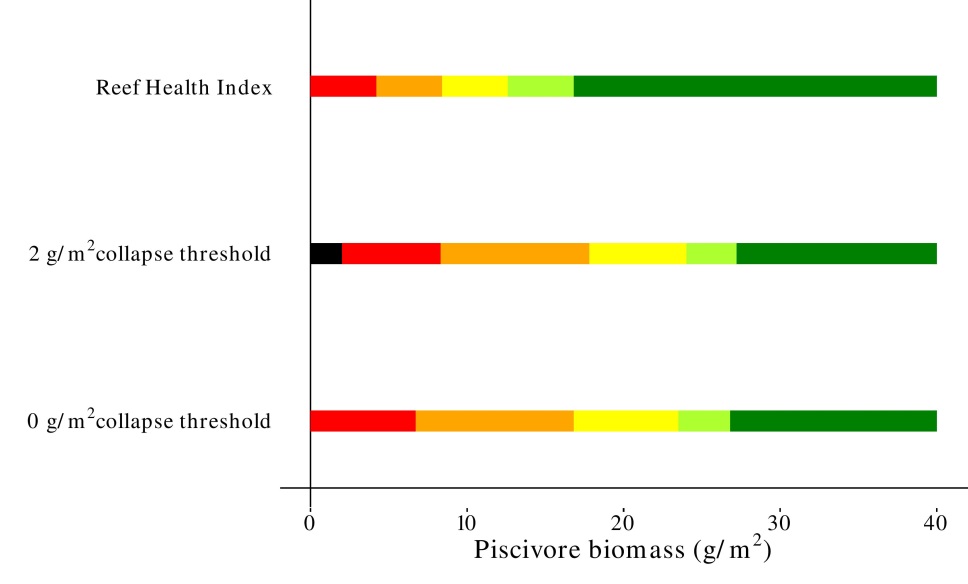


**Figure S11.** Levels of piscivorous fish biomass (g/m^2^) corresponding to the categories of the Reef Health Index and Red List of Ecosystems categories. Reef Health Index: dark green: very good; light green: good; yellow: fair; orange: poor; and red: critical. Red List of Ecosystems: dark green: Least Concern; light green: Near Threatened; yellow: Vulnerable; orange: Endangered; red: Critically Endangered; black: Collapsed.


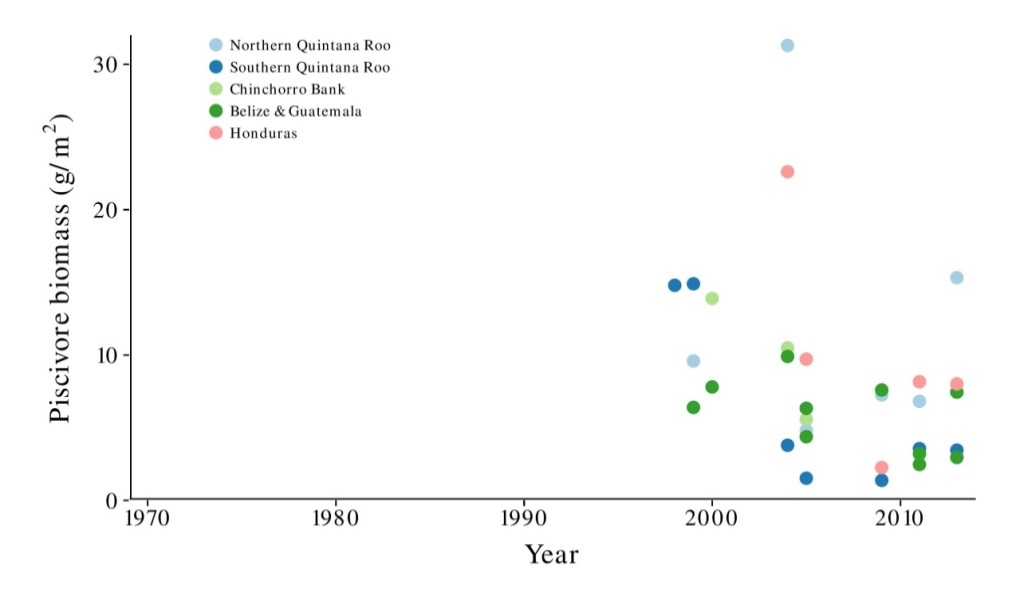


**Figure S12.** Piscivorous fish biomass in the Meso-American Reef between the years 1970-2013 in five subregions (n = 31).

## Urchin biomass and density

**1. Relevance to ecosystem function.** Sea urchins are important herbivores in Caribbean coral reefs, as they help to maintain the balance between coral and algal growth. *Diadema antillarum* (long-spined black urchin) was once the most abundant herbivore on Caribbean reefs. A lethal disease killed 98 % of the population in 1983, contributing to a shift from coral-dominated reefs to macroalgae-dominated reefs (Lessios 1988). Although urchins are important components of the MAR ecosystem, extinction of urchins may not cause total loss of ecological character if their ecological function is sustained by herbivorous fish. Urchin density is therefore not an appropriate indicator for criterion D, although it may provide a fuller picture of ecosystem degradation.

**2. Data availability and quality.** We collated data on urchin biomass (g/m^2^) (Melbourne-Thomas, Johnson et al. 2011) and data on urchin density (#/m^2^). It was not possible to transform urchin density into urchin biomass due to high variation in teste size and individual urchin biomass across the ecosystem (Brown-Saracino, Peckol et al. 2007). We therefore used urchin biomass as validation data for the ecosystem model (n = 4) and urchin density as indicator for criterion D (n = 17, Figure S13).

**3. Selection of a collapse threshold.** The Healthy Reefs Initiative considers a density of *Diadema antillarum* of less than one per m^2^ to indicate a “red flag” (McField and Kramer 2007).

**4. Selection of initial and current/future values.** Urchin density was 25.8 per m^2^ before the *Diadema antillarum* die-off in 1980 (Bauer 1980). Average densities for urchins in the MAR for the years 2011-2013 were 0.17 per m^2^. The decline in the population is therefore 99.4 %. This figure is close to the 99.9 % decline estimated in Belize by Lessios (1988).

**5. Shape of decline.** We did not determine the shape of the relationship between urchin biomass or density and progression towards ecosystem collapse, although it is likely to be linear.


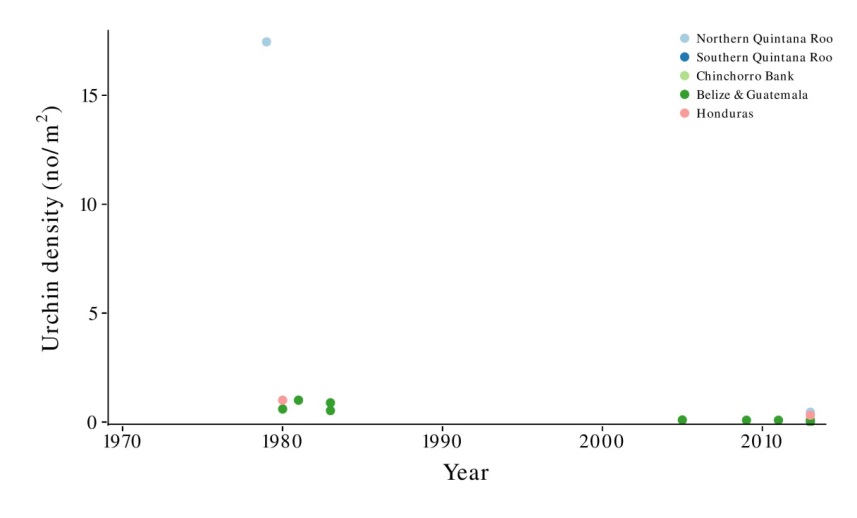


**Figure S13.** Urchin density in the Meso-American Reef between the years 1970-2013 in five subregions (n = 17). Urchin densities are very low following the occurrence of urchin disease in 1983-1984. Only one data point was collected before mass urchin die-off, hence it was not possible to derive a trajectory for urchin density.

## Elkhorn coral and staghorn coral presence

**1. Relevance to ecosystem function.** The elkhorn coral (*Acropora palmata*) and the staghorn coral (*Acropora cervicornis*) were dominant in Caribbean coral reefs over the last million years, but declined rapidly in the 1970s and 1980s (Pandolfi and Jackson 2001). They are now considered ecologically-extinct in many locations (Jackson, Donovan et al. 2014). Extirpation of *Acropora palmata* and *Acropora cervicornis* from the MAR would result in significant loss of characteristic biota and ecological character.

**2. Data availability and quality.** Few data are available to assess the status of the MAR against pre-industrial conditions (D3). Assuming that coral cover was similar in 1750 and in the 1960s, one would conclude that the status of the ecosystem is Endangered. However, large amounts of evidence point to pre-industrial impacts on Caribbean coral reefs due to land clearing and overfishing (Cramer, Jackson et al. 2012), hence obtaining historical data is paramount to assessing subcriterion D3. We obtained presence data across the Caribbean for *Acropora palmata* and *Acropora cervicornis* for the period 1851-2012 from Jackson et al. (2014), who based their analyses on paleocological and historical records from the region.

**3. Selection of a collapse threshold.** We assumed that ecosystem collapse would occur when the presence of either of these two species declines to zero in the entire Caribbean.

**4. Selection of initial and current/future values.** *Acropora palmata* was present in 87 % of sites across the Caribbean during the period 1851-1959, but was only present in 20 % of sites in the period 2005-2011 (Jackson, Donovan et al. 2014). *Acropora cervicornis* was present in 62 % of sites across the Caribbean during the period 1851-1959, but was only present in 8 % of sites in the period 2005-2011 (Jackson, Donovan et al. 2014). The relative severity of the historical decline across the whole ecosystem is 77 % for *Acropora palmata*, and 87 % for *Acropora cervicornis*. The ecosystem is Endangered under subcriterion D3 although there is uncertainty in the presence of both species within the MAR. This listing is concordant with the status obtained for all Caribbean coral reefs (Appendix S2 in Keith, Rodríguez et al. 2013) based on a semi-quantitative index of reef degradation integrated over seven functional groups (Pandolfi, Bradbury et al. 2003).

**5. Shape of decline.** We assumed a linear relationship between the presence of the two species and ecosystem collapse across the Caribbean.

# **Ecosystem model**

We provide a brief explanation of model components and dynamics; more details are available in Melbourne-Thomas et al. (2011, 2011, 2011). We used the ecosystem model in three steps:

1. Model reef condition under natural disturbance regimes, based on: i) historical coral cover data; and ii) contemporary data on coral cover from sites in good condition.

2. Historical reconstruction of the MAR based on a known scheduling of threats over the past 50 years. This enabled us to: i) validate model outputs with empirical data; and ii) assess subcriterion D1 for indicators lacking empirical data.

3. Projections of: i) future risk of collapse (criterion E); ii) future distribution (subcriterion A2a); and iii) future levels of biotic indicators (subcriterion D2a). Projections were based on local and global threat scenarios.

## Model components and local-scale dynamics

The components of CORSET are: a gridded base map identifying the location of coral reef cells; independent instantiations of a local-scale ecological model in each grid cell; a transport layer describing larval connectivity between all reef cells; and the modelled effects of disturbances (e.g. hurricanes, bleaching, and disease), fishing, and pollution. Local-scale dynamics are described by a mean-field model (Fung, Seymour et al. 2011) representing interactions between coral reef functional groups (Table S5). These groups are either benthic (brooding corals, spawning corals, macroturf, grazed epilithic algal communities) or consumer (herbivorous fish, small piscivorous fish, large piscivorous fish, sea urchins). The local-scale model operates in each 2 x 2 km cell of the gridded base map. Parameters are defined as ranges from the minimum and maximum values derived from ecological studies (Appendix A in Melbourne-Thomas, Johnson et al. 2011). Parameter values are selected at random from these ranges for each cell (assuming a uniform distribution, given the lack of further information), and are allowed to vary randomly among simulation years.

The mean-field model is a generic model of coral reef ecosystems, and assumes that each coral colony is a hemisphere and space is not limited. It is not species-specific. We note that Fung et al. (2011) derived the lateral growth rate based on the estimated minimum radius of a coral recruit in the Western Atlantic, linear skeletal extension distances, and identically distributed random variables representing variation in the radius of coral recruits. Skeletal extension distance was derived as the median from the values found in seven studies, and is unlikely to refer to species of the genus *Acropora,* which have unusually high growth rates but now only constitute a minority of corals present in the MAR (Huston 1985).

Three larval connectivity matrices are included in the model, one for each of: i) spawning corals; ii) fish; and iii) sea urchins. Connectivity matrices are derived from particle-tracking simulations, taking into account ocean currents as well as larval behaviour (mortality, vertical migration, and attraction towards settlement habitats) (Paris, Cherubin et al. 2007). Larval connectivity is linked to the local-scale model by means of larval production and post-settlement processes. Melbourne-Thomas et al. (2011) provide further details on the implementation of larval production and post-settlement processes in CORSET.

**Table S5.** Definitions of functional groups included in the model for the Meso-American Reef, adapted from Melbourne-Thomas et al. (2011).

| Functional group | Definition |
| --- | --- |
| Brooding corals | Hermatypic coral species with internal brooding of planula larvae followed by release of planulae (e.g. *Agaricia* spp., *Porites astroides*). |
| Spawning corals | Hermatypic coral species with broadcast spawning and external fertilization of gametes (e.g. *Montastrea annularis*). |
| Macroturf | Fast-growing, filamentous algae that form patches greater than ~2-4 mm canopy height (e.g. *Ceramium* spp., *Enteromoprha* spp.). |
| Macroalgae | Green, red, and brown algae with larger thallus size and greater structural complexity than macroturf (e.g. *Dictyota* spp., *Lobophora variegata*). |
| Grazed epilithic algal communities | Benthic cover characterized by non-geniculate coralline algae and dead coral skeletons. The most important feature of this functional group is that it is covered by a fine algal turf less than ~2-4 mm in height, maintained by grazing. |
| Herbivorous fish | Reef-associated fish that feed on epilithic algal communities, macroturf, and macroalgae (e.g. families Acanthuridae and Scari  dae). |
| Small piscivorous fish | Fish that prey on herbivorous fish, and are smaller than 60 cm in length. Some fish in this functional group remain small throughout their lifetime (e.g. some fish from families Lutjanidae and Serranidae), whilst a certain proportion grow into large piscivorous fish. |
| Large piscivorous fish | Fish that prey on herbivorous fish, and are larger than 60 cm in length (e.g. fish from families Carangidae and Lutjanidae). |
| Sea urchins | Grazing sea urchins (e.g. *Diadema antillarum*). |

CORSET implements external forcings in the form of disturbances (hurricanes, disease, bleaching), fishing, and pollution (sedimentation and nutrification) (Table S6). Hurricanes are tropical cyclones with minimum sustained wind speeds >33 m/s (Nyberg, Malmgren et al. 2007). Sedimentation is defined as the import and deposition of suspended sediments onto coral reefs, mostly via rivers and terrestrial runoff. Sedimentation is represented as the ecological effects of sediment deposition on coral growth, mortality and reproductive success. Nutrification is defined as an increase in the flux of nutrients into coastal waters (as opposed to eutrophication, which is an ecological process). Nutrients that typically enter coral reef systems are inorganic and organic forms of nitrogen and phosphorus, including nitrate, ammonium, soluble reactive phosphate and dissolved organic complexes that can be re-mineralized into organic forms. Because the rates of sedimentation and nutrification are similar in future pollution scenarios for the MAR (Burke and Sugg 2006), we grouped sedimentation and nutrification under the heading “pollution” for future risk scenarios.

**Table S6.** The effects of forcing on the ecosystem model, adapted from Melbourne-Thomas et al. (2011).

| Forcing | Effects |
| --- | --- |
| Hurricanes | Hurricanes are modelled as a decrease in coral cover by a factor of 0.002-0.7, and a decrease in macroalgal cover by a factor of 0-0.9 in affected reef cells. Category 1 and 2 hurricanes affect 25 % of reef cells in a sub-region; category 3 hurricanes affect 50 % of cells; and category 4 and 5 hurricane affect 75 % of cells in a subregion. |
| Coral mortality | Coral bleaching, disease and/or crown-of-thorns (*Acanthaster planci*) outbreaks are modelled as coral mortality events that directly decrease coral cover in affected cells. All reef cells in a subregions are affected by each coral mortality event (20 % to 60 % mortality). |
| Ocean acidification | The effects of ocean acidification are implemented as a decrease in coral growth rate, from the current rate of 0.04-0.2 yr^-1^ on epilithic algal communities. |
| Fishing | Fishing pressure is modelled as a decrease in fish biomass that is apportioned among herbivorous, small piscivorous and large piscivorous fish. |
| Sedimentation | Sedimentation decreases coral survival by a factor of 0-0.2 below natural rates per year, decreases survival of coral recruits by a factor of 0.6 and prevents recruitment of coral onto macroturf in affected reef cells. |
| Nutrification | Nutrification increases macroalgal growth by a factor 2-7, and decreases coral larval production by a factor of 0-0.25 in affected cells. |

## Initial reef scenario

We simulated the MAR under initial (i.e. pre-human impact) conditions to confirm that the model reached a steady-state in the absence of anthropogenic forcings. This constitutes one form of validation of the model parameters. In addition, this simulation can provide insights into subregional differences in the MAR that may affect its responses to disturbances and threats. As simulations for the initial reef scenario depend on the original amount of coral cover, we set initial covers using: i) historical data; and ii) contemporary data from sites in good condition.

### *Setting forcings*

Under the initial reef scenario, we ran the model in the absence of fishing, sedimentation and nutrification. No coral bleaching events linked to high sea surface temperature in the Caribbean have been recorded pre-1960s. Since then, one bleaching event was recorded in the Florida Keys in transplanted corals in 1961 (Shinn 1966), and one bleaching event was caused by low salinity following a hurricane in 1963 (Goreau 1964). We therefore did not simulate bleaching events in the initial reef scenario.

Under the initial reef scenario, we ran the model in the presence of background levels of hurricane frequency and intensity. We did not use the original background frequency proposed by Melbourne-Thomas et al. (2011) as the period 1970-1990 showed an anomalously low frequency of hurricanes (Nyberg, Malmgren et al. 2007). Instead, we obtained data from IBTRACTS and documented all hurricanes occurring in the study region with speeds >33 m/s (hurricanes category 1 and above). We documented 39 hurricanes in the period 1853-1965 leading to an estimate of return times of hurricanes of 2.89 years. As the model only includes integer values of long-term hurricane frequency, we set the return time of hurricanes to 3 years. The relative frequencies of hurricanes over the period 1853-1965 in each category were: category 1 (40%); category 2 (50%); category 3 (4%); category 4 (4%); and category 5 (2%). We set the relative frequencies of hurricanes in the model at these frequencies. The MAR is composed of five subregions, and all but one hurricane since 1853 have affected either one or two regions in the MAR. We set the maximum number of regions affected by each hurricane to two. The zones of influence of tropical hurricanes are respectively: 35 km diameter for categories 1 and 2 (962 km^2^); 60 km for category 3 (2827 km^2^); and 100 km for categories 4 and 5 (7854 km^2^) (Gardner, Cote et al. 2005). Given the relative sizes of each subregion (I-V), we assumed that hurricanes categories 1 and 2 would affect 25 % of cells in a subregion; category 3 would affect 50% of cells; and categories 4 and 5 would affect 75 % of cells. Affected cells were selected randomly. Hurricanes are modelled as a decrease in coral cover by a factor of 0.002-0.7 (Bythell, Gladfelter et al. 1993, Cheal, Coleman et al. 2002, Gardner, Cote et al. 2005) and a decrease in macroalgal cover by a factor of 0-0.9 in affected cells.

We used 10 Monte Carlo runs following scenario analysis undertaken by Melbourne-Thomas et al. (2011). We burnt-in the model for 5 years and assessed stability of trajectories over 100 years.

### *Using historical data to set initial values*

Given the large historical decline in reef condition in the MAR, contemporary data from sites in good condition may underestimate initial coral cover. We collated historical data to infer the initial state of the ecosystem (criterion D and Appendix S2). For the year 1970, we estimated a mean coral cover of 74.6 % in the Belize and Guatemala subregion, and a mean coral cover of 34.1 % in all other subregions. The weighted mean of overall coral cover in the MAR is therefore 56.5 %.

Historical data for Belize and Guatemala may not be representative if collected from sites with unusually high coral cover. An initial live coral cover of 80 % was reported in 1970 at Glover’s Reef, a remote atoll located 30 km from the coast of Belize. Coral cover was around 60 % when research started at Carrie Bow Cay in 1962, a site that is taken as representative of wider conditions on the Belize Barrier Reef (Jackson, Donovan et al. 2014). Given the high uncertainty in historical cover, we assumed a 60 % coral cover in Belize and Guatemala (rather than the higher mean estimate given above), with relative proportions of brooding and spawning corals as defined in Melbourne-Thomas et al. (2011). The relative proportions of the remaining groups (macroturf, macroalgae, and epilithic algal communities) remained identical to Melbourne-Thomas et al. (2011). The relative percentage of brooding corals, spawning corals, macroturf, macroalgae and epilithic algal communities were 45 %, 15 %, 30 %, 4 %, and 6 % in the Belize and Guatemala subregion. The relative percentage of brooding corals, spawning corals, macroturf, macroalgae and epilithic algal communities were 30 %, 10 %, 45 %, 5 %, and 10 % in the other subregions. In the absence of historical data for consumer groups, we set consumer biomass as in the “healthy reef” scenario defined by Melbourne-Thomas et al. (2011). The biomass of herbivorous fish, small piscivorous fish, large piscivorous fish, and sea urchins were 30, 10, 45, and 5 g/m^2^ respectively in all subregions.

### *Using data from sites in good condition to set initial values*

The Healthy Reef Initiative considers a live coral cover exceeding 40 % as “very good” (Healthy Reefs Initiative 2015), providing a minimum estimate of the amount of coral cover under initial conditions. We set initial benthic cover as in the “healthy reef” scenario defined by Melbourne-Thomas et al. (2011). The relative percentage of brooding corals, spawning corals, macroturf, macroalgae and epilithic algal communities were 30 %, 10 %, 45 %, 5 %, and 10 % in all subregions. We set initial consumer biomasses in all subregions as in the “healthy reef” scenario defined by Melbourne-Thomas et al. (2011), and outlined in the section above. Therefore the only differences between the initial values set with historical data versus data from sites in good condition were in the benthic covers of the Belize and Guatemala subregion.

### *Sensitivity to coral growth rates*

Coral growth rate is a high leverage parameter in the CORSET model, affecting both model outputs for initial reef scenarios and recovery scenarios (Melbourne-Thomas, Johnson et al. 2011). The current parametrization of coral growth rates includes *Acropora* species, although these are now rare in the Caribbean (McField and Kramer 2007). Because the growth rates of *Acropora* species far exceed those of other corals (Huston 1985), the current parametrisation of the CORSET model may overestimate coral growth rates and reef recovery from threats. For these reasons, we tested model sensitivity to growth rates by excluding *Acropora* species from our growth rates estimates.

The lateral growth rate of corals (for both brooders and spawners) implemented in CORSET was derived by Fung et al. (2011) and estimated at 0.04-0.2 proportion cover per year. The lateral growth rate (*r_c_*) (Equation 1) is based on the estimated minimum radius of a coral recruit in the Western Atlantic (M_1_) (Edmunds 2007), linear skeletal extension distance (*d*), and identically distributed random variables ($\bar{X}$ and $\sigma_{X}^{2}$) representing variation in the radius of coral recruits (derived from mixed species colonies in the Caribbean; Garcia-Salgado, Nava-Martinez et al. 2008).

yr^-1^, (Equation 1)

Linear skeletal extension distances were extracted from two studies (Huston 1985, Chornesky and Peters 1987), including 16 values for three *Acropora* species (*Acropora palmata, Acropora cervicornis, Acropora prolifera*) (Table S7). The median of all values was used to derive lateral growth rate (*d* = 0.9725 cm/y), which may therefore be influenced by *Acropora* species in Fung et al.’s (2011) parametrization. We used Fung et al.’s (2011) equation, minimum radius parameter and random variables to calculate a new median excluding linear extension distances for *Acropora* species (*d* = 0.705 cm/y), and estimated a new growth rate of 0.02595339- 0.1476035, which rounded to two decimal places gives 0.03-0.15 proportion cover per year. To estimate the sensitivity of the model to coral growth rate, we ran the initial reef scenarios based on historical and pristine estimates of coral cover with the new growth rate parameter,

**Table S7.** Skeletal extension rates (*d*) values for different corals in the Caribbean. Values for *Acropora* species (displayed in bold) were excluded from the median estimate of *d* to derive a lower estimate of coral growth rates. Data provided by Tak Fung.

| *d* **/ cm** | **Species** | **Location** | **Source** |
| --- | --- | --- | --- |
| **2.5-4.0** | *Acropora palmata* | Florida, western Atlantic | Huston (1985) |
| **8.8** |  | Curaçao, Caribbean |  |
| **4.7-8.3; 6.5,9.9** |  | VI, Caribbean |  |
| **4.0-4.5; 26.0** | *Acropora cervicornis* | Florida, western Atlantic; Jamaica, Caribbean |  |
| **7.0-13.0; 7.1** |  | Florida, western Atlantic; Curaçao, Caribbean |  |
| **10.9-15.9; 8.0-14.0; -; 9.2-14.8** |  | Jamaica, Caribbean |  |
| **3.7** | *Acropora prolifera* | Florida, western Atlantic |  |
| **5.9; 8.2** |  | VI, Caribbean |  |
| -; 2.4 | *Madracis asperula* | Jamaica, Caribbean |  |
| 0.48-1.72; -; 2.4 | *Agaricia agaricites* | Florida, western Atlantic; Curaçao, Caribbean |  |
| 0.15-0.23 | *Siderastrea radians* | Florida, western Atlantic |  |
| 0.15-0.50; 0.57-0.93; 0.27-0.42 | *Siderastrea siderea* | Florida, western Atlantic; Jamaica, Caribbean |  |
| 0.83-2.00; 3.6 | *Porites porites* | Florida, western Atlantic; Jamaica, Caribbean |  |
| 0.6-2.1; -; 0.7-1.5 |  | Florida, western Atlantic |  |
| 0.35-1.40 | *Porites astreoides* | Florida, western Atlantic |  |
| 0.34-0.35; 0.30 |  | VI, Caribbean |  |
| 0.22-0.63; 0.22-0.45; 0.25-0.31; 0.19-0.26 |  | Jamaica, Caribbean |  |
| 0.9-2.28 | *Porites furcata* | Florida, western Atlantic |  |
| 0.35-1.00 | *Diploria strigosa* | Florida, western Atlantic |  |
| 0.40-0.88 | *Diploria clivosa* | Florida, western Atlantic |  |
| 0.92-1.05; 0.57-0.93; 0.30-0.51 | *Colpophyllia natans* | Jamaica, Caribbean |  |
| 0.6; 2.4 | *Montastraea annularis* | Florida, western Atlantic; Jamaica, Caribbean |  |
| 0.92-1.00; 0.97-1.04; 0.21-0.65; 0.16 |  | VI, Caribbean |  |
| -; 0.48-0.67; -; 0.15-0.21 |  | Jamaica, Caribbean |  |
| 0.66-0.83; 0.7-0.89 |  | VI, Caribbean |  |
| -; 0.63-0.78 |  | Curaçao, Caribbean |  |
| 0.28-1.22; 0.59-1.13; 0.1-0.27; 0.12-0.21 |  | Jamaican, Caribbean |  |
| -; 0.20-0.68; 0.36-1.09; 0.23-0.57 | *Montastraea cavernosa* | Jamaica, Caribbean |  |
| 0.2-1.8 | *Porites astreoides* | W. fore-reef, Discovery Bay, Jamaica, Caribbean | Chornesky and Peters (1987) |

### *Results*

Coral cover at the end of the 100-year simulation was stable based on both historical data and data from sites in good condition (Figure S14). In the initial reef scenario based on historical data, the final mean coral cover (40.6 %) was lower than the mean coral cover initially set (51.1 %). We tested whether this decrease in coral cover was due to the effect of hurricanes by removing them, but coral cover remained similar in its absence (results not shown). The final mean coral cover was not significantly different when the model was initiated on good condition site data (41.1 %; Wilcoxon rank-sum test: W = 47, p = 0.853), but the model converged to this value faster than when calibrated with historical data (Figure S14). The model therefore converges to a relatively high coral cover in the presence of natural levels of forcings despite different initial coral covers. Coral cover also converged to values ~ 40 % in sensitivity analyses initiated with 1 %, 30 %, 60 %, and 90 % coral cover (Melbourne-Thomas, Johnson et al. 2011). The choice of initial values therefore had little impact on model trajectories.

Sensitivity analyses with CORSET reveal that herbivorous fish biomass reaches steady state values of ~ 28 g/m^2^ whether the model is initiated with 5, 20, 40, or 60 g/m^2^ herbivorous fish biomass (Melbourne-Thomas, Johnson et al. 2011), a result corroborated by our analysis. The estimated herbivorous fish biomass therefore seems constrained by the model structure and parameters. Predicted herbivorous fish biomass was lower than values for “very good” condition in the Reef Health Index (>34.8 g/m^2^; Healthy Reefs Initiative 2015), but grazing pressure from herbivorous fish and urchins was sufficient to constrain algal overgrowth of coral. Indicators of reef health are not available for macroturf, EAC, piscivorous fish biomass, and sea urchin biomass.

Initiating the model based on historical data or good condition site data made little difference to community composition in the MAR. We therefore used contemporary data from sites in good condition to instantiate our historical reconstruction as these: i) represent a more parsimonious hypothesis concerning the initial state of the system (we assume all regions have similar cover); ii) lead to faster model convergence; and iii) have been used by for sensitivity analysis and scenario analysis in the MAR (Melbourne-Thomas, Johnson et al. 2011, Melbourne-Thomas, Johnson et al. 2011).

Using a lower estimate of coral growth rate (excluding *Acropora* species) led to lower estimates for coral cover, small piscivorous fish, large piscivorous fish, and higher estimates for macroturf (Figure S15). Herbivores were not affected, implying dampening or compensatory effects at this intermediate trophic level. Coral cover only stabilized in the last 25 years of the simulation in the models parametrized with low coral growth rates, and stabilized at much lower values than in the models parametrized with high growth rates. The mean coral cover at the end of the simulation based on historical data was 21.4 % (20.4 % based on sites in good condition), almost half of the coral cover estimated in matching scenarios with high coral growth rates (Figure S14). There were significant differences in initial reef scenarios based on historical data but different coral growth rates (Wilcoxon rank-sum test: W = 100, p < 0.001), and based on data from sites in good condition but different coral growth rates (Wilcoxon rank-sum test: W = 100, p < 0.001).

These results indicate that when parametrized with low growth rates, the model is incapable of producing stable coral covers as high as observed in the historical records, or assumed to be pristine under the Reef Health Index. While it is likely that historical data overestimate true past coral cover (as observations are often recorded from marine parks), and that current conservation benchmarks may provide unrealistic “stretch” targets, it is unlikely that historical coral cover was ~ 20 % in the MAR. It therefore seems that in conjunction with other model and forcing parameters, the lower estimate of coral growth rate cannot reproduce initial reef conditions (i.e. relatively high coral cover). Median skeletal extension distance and corresponding growth rates including *Acropora* species may be more representative of historical dynamics in the MAR, possibly because the historical MAR included fast-growing Acroporids at high abundances. A possible interpretation would be that in the absence of *Acropora* species, coral cover in the MAR would decline rapidly in the presence of natural levels of disturbance, due to low growth rates and recovery of other corals.

One must also be cautious in interpreting coral growth rates as modelled in a mean-field model designed to depict generic, circular coral colonies of undetermined species (Fung, Seymour et al. 2011). Both estimated median skeletal extension distances (*d* = 0.9725 and *d* = 0.705 cm/y) correspond to skeletal extension distances of many species, including non-Acroporids. There may be sampling biases in reporting skeletal extension distances (Table S7) and further uncertainty is introduced by combining those with the radius of an ‘average’ coral recruit in the Caribbean, and estimates of variation in coral growth rate extracted from the literature (Garcia-Salgado, Nava-Martinez et al. 2008). It is therefore difficult to define a ‘true’ growth rate for an average coral colony in the MAR, so parameter estimates of the CORSET model must be interpreted as generic estimates based on the best available literature. Indeed, the estimates of skeletal extension rates were not weighted according to the relative abundance of species in the ecosystem, so only the inclusion/exclusion of some species (rather than changes in abundance) can be assessed. The CORSET model is based on dozens of parameters that have been extensively tested during model-building (Melbourne-Thomas 2010), so modifying the coral growth rate may lead to sub-optimal outcomes if other parameters have been over-estimated (e.g. algae growth rates). Because parameters are sampled from uniform distributions based on the literature, sampling issues may affect all parameters.


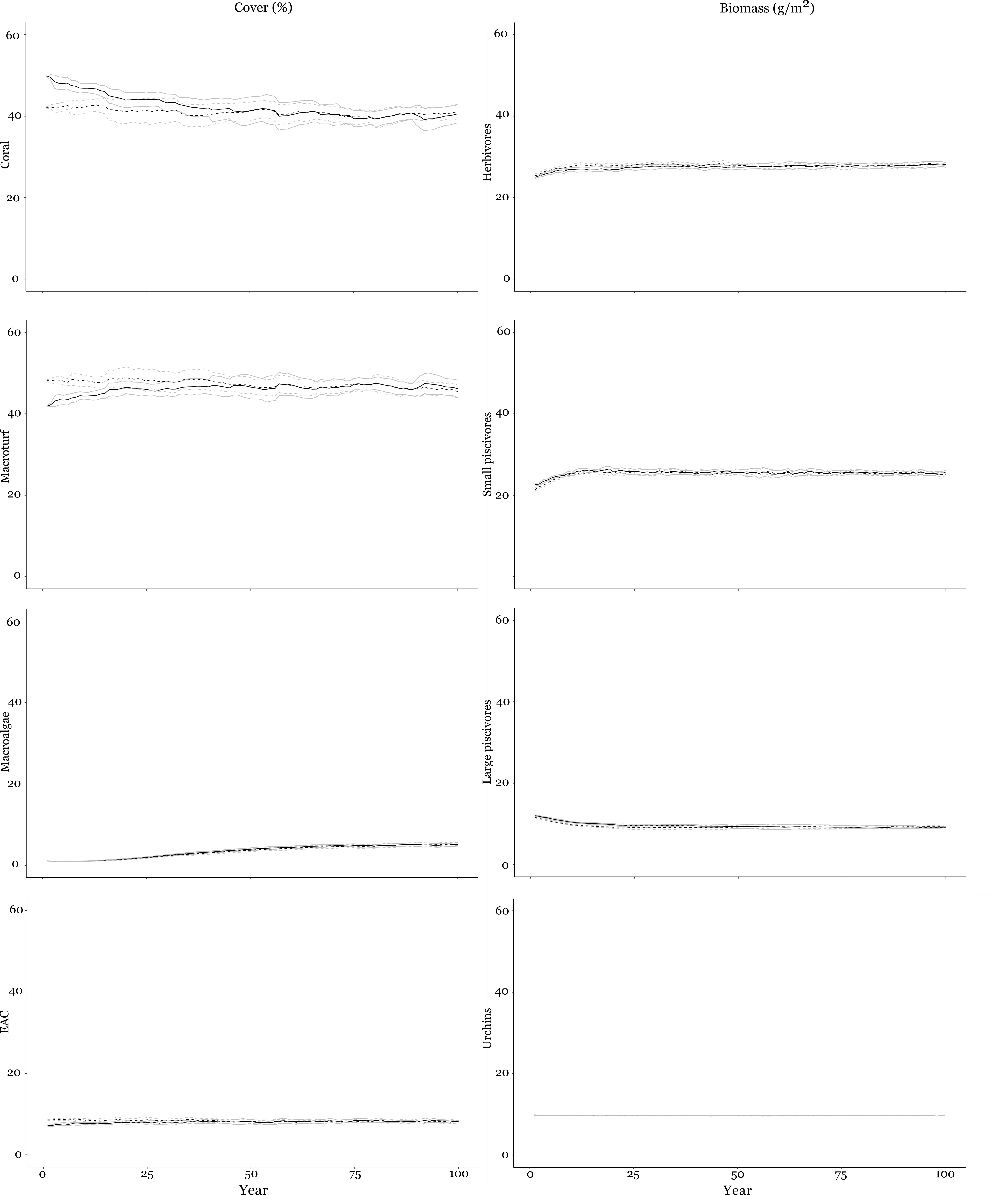


**Figure S14.** Model trajectories for benthic and consumer groups over 100 years under the initial reef scenario, based on historical data (full lines) and contemporary data from sites in good condition (dashed lines) and high estimates of coral growth rates (including *Acropora* species). Grey lines indicate 95% confidence intervals based on 10 model runs. Initial levels of forcing correspond to estimated natural hurricane frequency and intensity. EAC: epilithic algal communities.


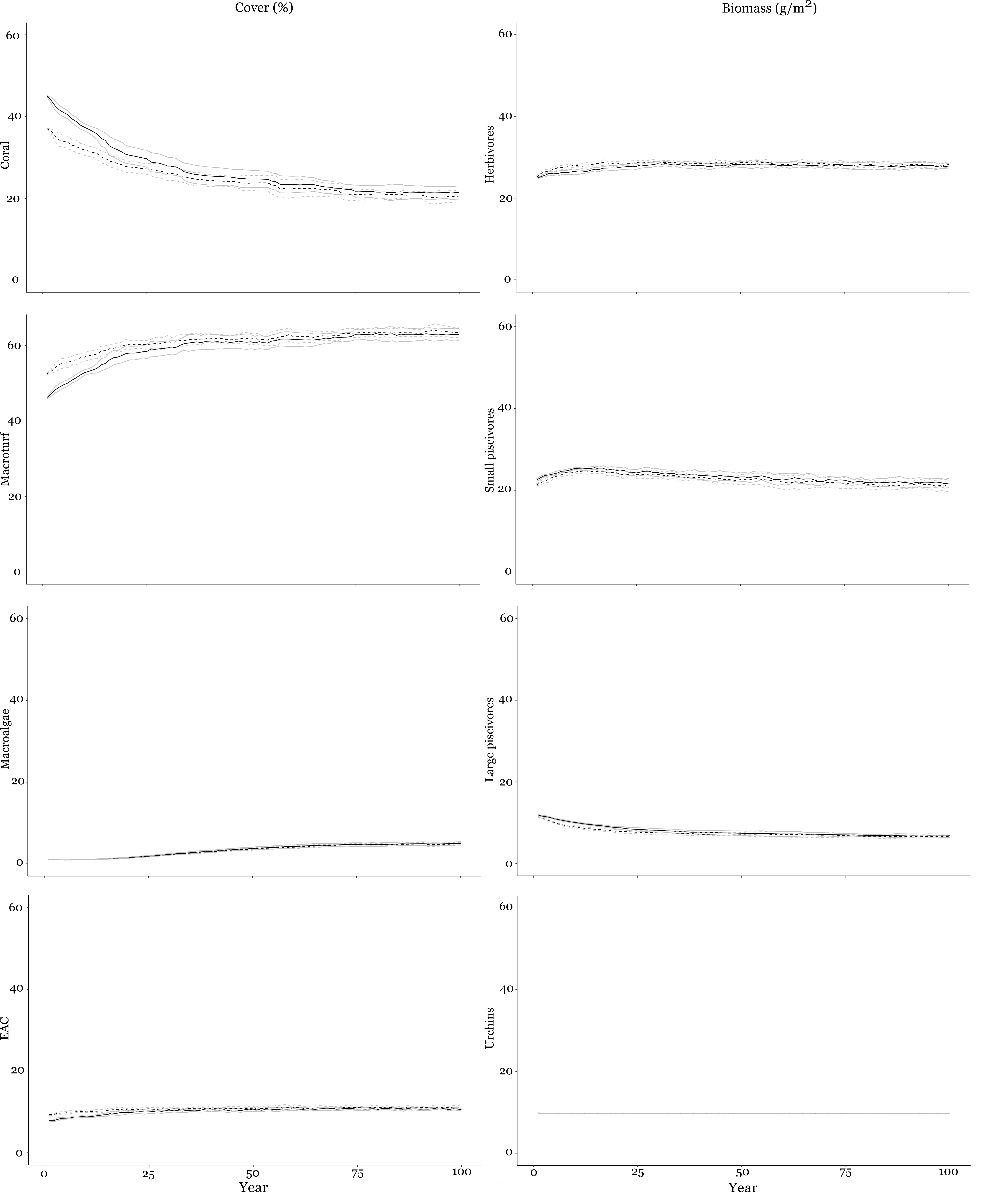


**Figure S15.** Model trajectories for benthic and consumer groups over 100 years under the initial reef scenario, based on historical data (full lines) and contemporary data from sites in good condition (dashed lines) and low estimates of coral growth rates (excluding *Acropora* species). Grey lines indicate 95% confidence intervals based on 10 model runs. Initial levels of forcing correspond to estimated natural hurricane frequency and intensity. EAC: epilithic algal communities.

## Historical reconstruction

### *Setting forcings*

We used data from Melbourne-Thomas et al. (2011) for the reconstruction years 1980-2007. We complemented the dataset with information on forcings from 1966-1979 and 2008-2015 (Table S8). There were no hurricanes after 2012 (<http://www.nhc.noaa.gov/>). We modelled the 1983-1984 sea urchin mortality event as a 99 % reduction in biomass (criterion D and Lessios 1988). We modelled the decline in coral cover between 1986 and 1990 attributed to white-band disease (Aronson and Precht 2001) as 20 % reduction in coral cover in Belize & Guatemala. The 1995 and 2005 coral bleaching events were not included in the historical scenario since they caused minimal mortality in the MAR (McField, Bood et al. 2008). We modelled the 1998 bleaching event as 20-60 % mortality across subregions, sampled for each cell from a uniform distribution (Steneck and Lang 2003, McField, Bood et al. 2008). We modelled the increase in coastal development over the last 50 years as a progressive increase in the number of reef cells affected by sedimentation and nutrification. As sediments and nutrients can be transported large distances from land sources (Chérubin, Kuchinke et al. 2008, Paris and Chérubin 2008, Carilli, Prouty et al. 2009), we assumed that sedimentation and nutrification affected reefs close to the coast as well as offshore atolls. We assumed no human impact due to coastal development in 1966 and a linear increase in impact until 2015. We calibrated relative levels of sedimentation and nutrification among regions based on Burke & Sugg (2006). The percentage of reef cells affected by nutrification in 2015 was therefore: Northern Quintana Roo (40 %), Southern Quintana Roo (2 %), Chinchorro Bank (0 %), Belize and Guatemala (6 %), and Honduras (32 %). The percentage of reef cells affected by sedimentation in 2015 was therefore: Northern Quintana Roo (0 %), Southern Quintana Roo (0 %), Chinchorro Bank (0 %), Belize and Guatemala (30 %), and Honduras (3 %).

Quantitative data on historical levels of fishing in the MAR are lacking. There has been a steady increase in fishing pressure in the MAR since 1980, and large piscivorous fish have been preferentially targeted (Arrivillaga and Garcia 2013). We modelled a linear increase over 50 years from the smallest recent estimate of fishing pressure to the highest estimate of fishing pressure in each subregion (Melbourne-Thomas, Johnson et al. 2011). The fishing rate in 1966 was 300 kg km^-2^ yr^-1^ in all subregions. The fishing rate in 2015 was 7650 kg km^-2^ yr^-1^ in the Belize and Guatemala subregion, and 4710 kg km^-2^ yr^-1^ in all other subregions. We did not include differences in fishing pressure between protected and non-protected areas given the absence of historical data.

We ran the historical reconstruction based on good condition site data, and with both low and high estimates of coral growth rates. We used 10 Monte Carlo runs and burnt-in the model for 5 years and assessed trajectories over 50 years (1966-2015).

**Table S8.** Chronology of modelled threats for the Meso-American Reef (MAR) for the period 1966-2015, adapted from Melbourne-Thomas et al. (2011). Subregion I: Northern Quintana Roo; II: Southern Quintana Roo; III: Chinchorro Bank; IV: Belize and Guatemala; V: Honduras. Categories refer to hurricane strength on the Saffir-Simpson scale as assessed by NOAA (<http://www.nhc.noaa.gov/>). Bleaching events occurring in 1998 and 2005 did not cause significant coral mortality (McField, Bood et al. 2008).

| Year(s) | Subregions | Forcing |
| --- | --- | --- |
| 1966-2015 | Entire MAR | Increasing fishing pressure and sediment/nutrient inputs from coastal development. |
| 1967 | I | Hurricane Beulah (category 2). |
| 1969 | IV, V | Hurricane Francelia (category 3). |
| 1971 | IV, V | Hurricane Edith (category 1). |
| 1974 | II, III | Hurricane Carmen (category 4). |
| 1974 | IV, V | Hurricane Fifi (category 2). |
| 1978 | IV, V | Hurricane Greta (category 3). |
| 1983-1984 | Entire MAR | Urchin mortality event (Lessios 1988). |
| 1986-1990 | IV | Decline in coral cover due to white band disease (Aronson and Precht 2001). |
| 1988 | I | Hurricane Gilbert (category 5) |
| 1998 | I, II, III, IV, V | Coral bleaching event (Steneck and Lang 2003). |
| 1998 | IV, V | Hurricane Mitch (category 5) |
| 2000 | IV | Hurricane Keith (category 3) |
| 2001 | IV, V | Hurricane Iris (category 4) |
| 2002 | I | Hurricane Isidore (category 3). |
| 2005 | I | Hurricanes Wilma and Emily (category 4) |
| 2007 | II, III | Hurricane Dean (category 4). |
| 2010 | IV, V | Hurricane Richard (category 2). |
| 2012 | II, III | Hurricane Ernesto (category 2). |

### *Assessing model skill*

We used four skill assessment metrics to fully capture all differences between model reconstruction and empirical data (Stow, Jolliff et al. 2009, Olsen, Fay et al. 2016): Root Means Squared Error (RMSE), Absolute Average Error (AAE), Spearman correlation, and Modelling Efficiency. RMSE and AAE assess the accuracy of the model. RMSE and AAE both measure deviations of predicted values with observed values, with RMSE being more sensitive to data points with extreme deviations (Olsen, Fay et al. 2016). RMSE and AAE are not comparable among models using different units. Correlation metrics such as Spearman correlation focus on precision, as a model can show high correlation for results that have low accuracy (i.e. be consistently off-target). A correlation of 1 indicates perfect prediction, whereas a correlation of -1 indicates perfect inverse prediction.

Modelling Efficiency is the most stringent model skill metric used in this analysis. Modelling Efficiency takes into account both accuracy and precision and represents predicted performance compared to the mean of empirical observations. A value of 1 indicates perfect prediction, > 0 good prediction, and < 0 predictive ability lower than the mean of empirical observations. Modelling Efficiency is most sensitive to errors in scaling and inverse variations, rather than lack of correlation or trend mismatch (Olsen, Fay et al. 2016).

Correlation between model outputs (instantiated with high estimates of coral growth rates) and empirical data was low (0.41) for coral cover in the MAR as a whole, but some regions (Southern Quintana Roo and Honduras) showed higher correlation (> 0.76) (Table S9). Root Mean Squared Error and Absolute Average Error showed similar patterns among indicators and regions (Table S9). Modelling Efficiency was generally negative, indicating that reconstructed ecosystem dynamics do not provide a better fit compared to the mean of empirical observations (Table S9).

Inspection of the reconstruction and empirical data revealed that coral cover tends to be consistently overestimated or underestimated in some subregions (Figure S16). Herbivorous and piscivorous fish biomass were overestimated in Belize and Guatemala, and underestimated in Chinchorro Bank. Piscivorous fish biomass was consistently overestimated, which may be due to our assumption of a linear (rather than quadratic) increase in fishing over the time period.

In the whole MAR, the historical reconstruction instantiated with a lower estimate of coral growth rate (excluding *Acropora* species) showed a worse fit for coral cover under all skill assessment metrics (Table S9). Skill was improved for some subregions (Northern Quintana Roo, Southern Quintana Roo) but worsened for some subregions (Chinchorro Bank, Honduras) (Table S9). Skill generally improved for herbivorous and piscivorous fish biomass, but there were also worsening trends in some subregions (Belize & Guatemala). However, given the small number of validation data points for both herbivorous and piscivorous fish, model performance metrics may be more accurate and representative for the coral cover indicator. Coral cover predicted throughout the historical reconstruction was lower under low estimates of growth rate than under high estimates of growth rate (Figure S17). This may be because the model instantiated with lower growth rates cannot maintain historical coral cover (~ 40 %) even under small disturbances caused by natural levels of hurricanes. Under low estimates of growth rate, coral cover was estimated at 9.3 % in 2015, compared to 14.4 % under high growth rates, and ~ 16-18 % in the literature (2009-2014; Healthy Reefs Initiative 2015). Low estimates of coral growth rates therefore underestimate present coral cover. However, neither the high or low estimates of coral growth rates are completely appropriate for the historical reconstruction, as Acroporids became less abundant halfway through the model simulation (in the 1980s), so either parametrizations may over-estimate or under-estimate the influence of Acroporids in the MAR ecosystem. Further modifications to the CORSET model could be made to dynamically incorporate changes in growth rates caused by changes in species’ relative abundances. Because the model parametrized with lower coral growth rates presented worse model performance metrics for the coral cover indicator, and because Acroporids have not completely disappeared from the MAR, we used the higher growth rate parametrization to model reef futures, noting that this parametrization may over-estimate coral cover.

Validating ecosystem models is paramount to understanding system dynamics, identifying areas for model improvement, and increasing uptake of models in conservation decision-making (Stow, Jolliff et al. 2009, Addison, Rumpff et al. 2013). We find different patterns among performance metrics and subregions, highlighting the usefulness of multiple metrics for assessing model skill (Stow, Jolliff et al. 2009, Olsen, Fay et al. 2016). Importantly, a model with high correlation but poor modelling efficiency due to scale mismatch can be used to accurately assess relative severity of functional declines if the threshold for collapse is zero. Our skill metrics compare positively with quantitative assessments of other “whole-of-ecosystem” models, which remain scarce (Fulton, Smith et al. 2005, Olsen, Fay et al. 2016). Validating models by assessing both steady-state behaviour and historical reconstructions may not be possible for all ecosystems, as limited time series data tend to be used for fitting rather than validation (Scott, Serpetti et al. 2016). Model simulation experiments (Arnold and Dey 1986) can be undertaken to identify areas of high model sensitivity, and thus pinpoint what monitoring data are most needed to improve the reliability of the model and the risk assessment.

Our study also highlights important issues in assessing model skill when ecosystems have severely changed in structure and species composition. In our case, fast-growing *Acropora* species became less abundant halfway through our reconstruction scenario, and are expected to remain rare in the future, yet model skill was improved when incorporating *Acropora* species. Modelling reef futures under new species composition may rely on limited model validation if shifts in species composition have occurred relatively recently. Whether to calibrate models on past empirical data when present ecosystem structure is severely altered requires further examination. While many ecosystem models rely on assumptions of identical species composition through time (e.g. Ecopath; Watermeyer et al. 2008) or provide little opportunity to alter species’ relative abundances (e.g. CORSET), these assumptions may need to be revised in the presence of large-scale ecosystem shifts, novel ecosystems, and no-analog communities.

**Table S9.** Model performance metrics for the historical reconstruction (1966-2015) of the changes in biotic indicators of the Meso-American Reef (MAR). Models were parametrized on either high or low estimates of coral growth rates (including or excluding *Acropora* species, respectively). RMSE: Root Mean Squared Error; AAE: Absolute Average Error; Cor.: Spearman Rank Correlation; MEF: Modelling Efficiency. n: number of data points per indicator and subregion combination.

|  |  | Coral cover | | | |  | Herbivorous fish biomass | | | |  | Piscivorous fish biomass | | | |
| --- | --- | --- | --- | --- | --- | --- | --- | --- | --- | --- | --- | --- | --- | --- | --- |
|  | n | RMSE | AAE | Cor. | MEF | n | RMSE | AAE | Cor. | MEF | n | RMSE | AAE | Cor. | MEF |
| *High coral growth rate* | | | | | | | | | | | | | | | |
| MAR | 55 | 16.8 | 10.1 | 0.41 | 0.22 | 30 | 6.9 | 6.6 | 0.93 | -3.5 | 31 | 11.8 | 11.1 | 0.79 | -10.1 |
| Northern Quintana Roo | 14 | 13.3 | 10.3 | 0.03 | -1.1 | 6 | 14.2 | 13.1 | 0.14 | -0.01 | 6 | 10.7 | 7.1 | 0.14 | -0.41 |
| Southern Quintana Roo | 7 | 5.1 | 4.5 | 0.77 | 0.18 | 6 | 8.4 | 6.7 | -0.1 | 0.13 | 7 | 4.2 | 3.5 | 0.68 | 0.44 |
| Chinchorro Bank | 4 | 8.4 | 5.7 | 0.6 | -0.19 | 3 | 21.0 | 19.8 | -1 | -7.8 | 3 | 10.5 | 9.9 | 1 | -8.4 |
| Belize & Guatemala | 25 | 20.9 | 13.7 | 0.29 | 0.06 | 10 | 17.1 | 16.9 | 0.93 | -25.4 | 10 | 22.2 | 21.1 | 0.64 | -111 |
| Honduras | 5 | 7.5 | 6.0 | 0.8 | -1.4 | 5 | 19.2 | 16.5 | -0.5 | -3.6 | 5 | 6.1 | 4.0 | 0.7 | 0.19 |
| *Low coral growth rate* | | | | | | | | | | | | | | | |
| MAR | 55 | 18.3 | 10.9 | 0.49 | 0.07 | 30 | 5.0 | 4.7 | 0.93 | -1.4 | 31 | 8.5 | 6.9 | 0.79 | -4.7 |
| Northern Quintana Roo | 14 | 10.9 | 8.3 | 0.03 | -0.44 | 6 | 14.9 | 14.0 | 0.09 | -0.09 | 6 | 12.1 | 8.1 | 0.14 | -0.80 |
| Southern Quintana Roo | 7 | 3.1 | 3.0 | 0.77 | 0.7 | 6 | 8.8 | 7.7 | 0.03 | 0.05 | 7 | 4.6 | 3.4 | 0.68 | 0.32 |
| Chinchorro Bank | 4 | 11.2 | 9.0 | 0.6 | -1.1 | 3 | 21.1 | 19.9 | -0.5 | -7.8 | 3 | 10.5 | 9.9 | 1 | -8.5 |
| Belize & Guatemala | 25 | 21 | 13.7 | 0.29 | 0.06 | 10 | 14.8 | 14.7 | 0.96 | -18.7 | 10 | 16.9 | 14.4 | 0.64 | -64.0 |
| Honduras | 5 | 11.3 | 10.2 | 0.1 | -4.5 | 5 | 23.2 | 21.3 | -0.5 | -5.8 | 5 | 7.6 | 5.9 | 0.7 | -0.29 |


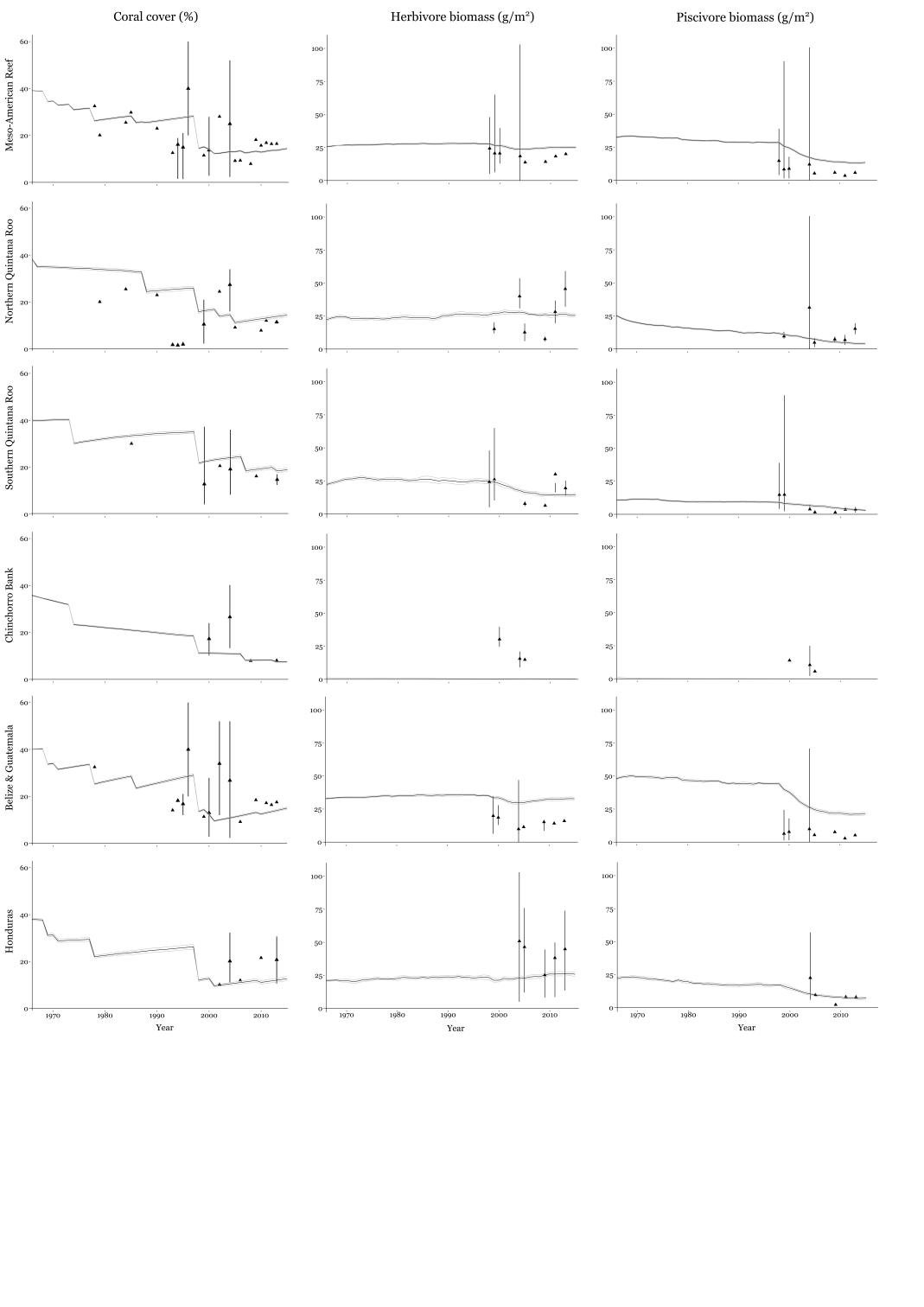


**Figure S16.** Comparison of model trajectories for coral cover, and biomass of herbivorous and piscivorous fish in the Meso-American Reef and its subregions, based on high estimates of coral growth rates (including *Acropora* species). Solid lines indicate mean trajectories from ten model runs, and grey lines indicate 95% confidence intervals across the runs. Vertical bars and triangles indicate minimum-maximum ranges and mean values from empirical observations, respectively. Piscivorous fish biomass is the sum of small and large piscivororous fish biomasses. Herbivorous and piscivorous fish biomass for Chinchorro Bank were low (maximum values of 1.2 g/m^2^) at the beginning of the reconstruction.


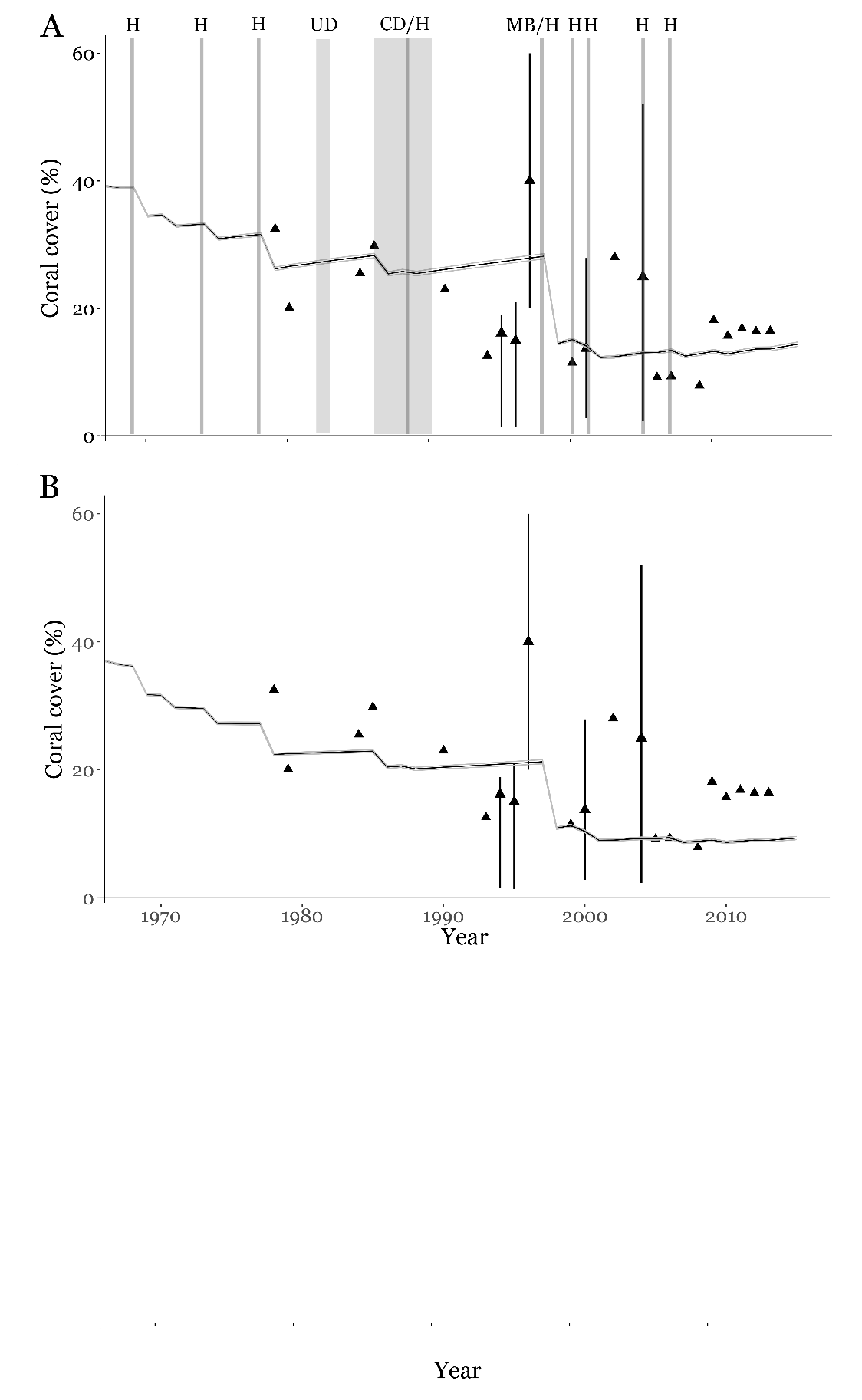


**Figure S17.** Comparison of model trajectories in the Meso-American Reef with empirical data on coral cover and threat impacts, for models parametrized on (A) high estimates of coral growth rate and (B) low estimates of coral growth rates (excluding *Acropora* species). Dark grey lines (H) in panel (A) indicate years with hurricanes categories 3 and above or mass bleaching events (MB). Light grey blocks indicate periods of urchin disease (UD) and coral disease (CD). Solid lines indicate mean trajectories from ten model runs, and grey lines indicate 95% confidence intervals among runs. Black vertical bars and triangles indicate minimum-maximum ranges and mean values from empirical observations, respectively, available for the period 1995-2005.

### *Applying subcriterion D1*

We used three biotic indicators to calculate relative severity of decline for the MAR and its subregions based on subcriterion D1: coral cover, herbivorous fish biomass, and piscivorous fish biomass. We used the collapse thresholds as determined under criterion D with empirical data. Based on the relative severity of decline in coral cover, the ecosystem is listed as Endangered under subcriterion D1 (Table S10). The mean herbivorous fish biomass at the start of the historical reconstruction (1966) was 27.7 g/m^2^, based on the model parametrized with high coral growth rates. The ecosystem is considered to be of Least Concern for the indicator based on herbivorous fish biomass. The mean piscivorous fish biomass at the start of the historical reconstruction (1966) was 33.5 g/m^2^. The ecosystem is considered to be of Endangered for the indicator based on piscivorous fish biomass.

We repeated the analysis with the model parametrized with low coral growth rates: risk assessment outcomes for herbivorous fish biomass remain unchanged, whilst risk is deemed higher based on the coral cover and piscivorous fish biomass indicators. Notably, the estimate of relative severity and risk assessment outcome for the coral cover indicator no longer match the declines estimated with empirical data (62-64 % decline; Endangered). The parametrization based on low coral growth rates therefore over-estimates risks and provides a worse fit to empirical data (Table S9).

**Table S10.** Relative severities of decline based on the historical reconstruction, for: a) coral cover; b) herbivorous fish biomass; and c) piscivorous fish biomass.

|  | **Relative severity (%)** | **Category** | **Relative severity (%)** | **Category** |
| --- | --- | --- | --- | --- |
| *Coral cover* | 0 % threshold |  | 1 % threshold |  |
| High coral growth rate | 63.2 | EN | 64.9 | EN |
| Low coral growth rate | 74.8 | CR | 76.8 | CR |
| *Herbivorous fish biomass* | 0 g/m^2^ threshold |  | 5 g/m^2^ threshold |  |
| High coral growth rate | 2.2 | LC | 2.8 | LC |
| Low coral growth rate | 7.4 | LC | 9.2 | LC |
| *Piscivorous fish biomass* | 0 g/m^2^ threshold |  | 2 g/m^2^ threshold |  |
| High coral growth rate | 58.6 | EN | 62.4 | EN |
| Low coral growth rate | 77.2 | CR | 82.3 | CR |

## Future estimates of risk

### *Setting forcings and initial values*

Scenarios are “plausible stories about how the future might unfold” (Biggs, Raudsepp-Hearne et al. 2007). We combine high-impact and high-uncertainty factors based on scenario sweeps in the MAR (Melbourne-Thomas, Johnson et al. 2011) and gaps in the literature. We investigate variation in levels of fishing, pollution (nutrification and sedimentation), bleaching, and ocean acidification. Given the lack of information on the most likely future threat levels, we determine low and high levels for each of these threats. We realise that these two treatments represent simplified versions of plausible futures. Whilst more detailed representations of some threats (e.g. mass bleaching) might be available, information on local stressors is extremely scarce so we could only derive low and high scenarios. In addition, assessment under the Vulnerable category for criterion E requires predictions over the next 100 years, which creates considerable uncertainty in model predictions.

*Fishing.* Determining future levels of fishing in the MAR is difficult given the paucity of data on current fishing levels at a regional scale, and the difficulties in predicting fine-scale socio-ecological relationships that determine fishing levels (Melbourne-Thomas, Johnson et al. 2011). For example, livelihood shifting is likely to have occurred in Quintana Roo in the last 30 years in response to the tourism boom. Whether this process will continue in the future cannot be determined. We did not model the effect of no-take zones as these are very small in size (especially compared to the scale of the model) e.g. 3 % of the reserve area in Chinchorro Bank and effectively 0 % in Sian Ka´an (Fulton, Bourillón et al. 2014). These may be important for the recruitment of species such as the Endangered Nassau grouper (*Epinephelus striatus*) as well as other fish and coral species. Future work could take into account simulations of the establishment of no-take zones. The *low scenario* for fishing randomly sampled fishing levels between minimum current levels and the median current reported value for the region (0.08-2.54 g/m^2^/y) (Halls, Burn et al. 2002). The *high scenario* for fishing randomly sampled fishing levels from the median current level to the maximum current level (2.54-5.0 g/m^2^/y) (Koslow, Aiken et al. 1994).

*Pollution (nutrifrication and sedimentation).* Burke & Sugg (2006) modelled the delivery of sediment, nitrogen, and phosphorus in the MAR with three different land-use scenarios of increasing damage (sustainability first; policy first; markets first) over the period 2003/2004-2025 (about 20 years). Changes in delivery for these three scenarios were respectively: sediments (-5 %; +5 %; +13 %); nitrogen (-4 %; +3 %; +8 %); and phosphorus (-5 %; +5 %; +11 %). We generalised these results to a 10 % increase in both sedimentation and nitrification over 20 years. Under the *high scenario*, we simulate a linear increase from current levels of sedimentation and nutrification to a 50 % increase after 100 years. Under the *low scenario*, we simulate a linear decrease from current levels of sedimentation and nutrification to a 25 % decrease after 100 years*.*

*Hurricanes.* A consensus of studies and model types point to a constant hurricane frequency in the future, with an increase in the frequency of hurricanes categories 4 and 5 (Knutson, McBride et al. 2010, Emanuel 2013, Knutson, Sirutis et al. 2013). Under the *high scenario*, we model future hurricane scenarios at an overall frequency similar to the historical baseline, but with severity increasing at a rate proportional to the number of simulation years. Hence at the end of the simulation (2116), the overall frequency of categories 4 and 5 hurricanes is 20 % compared to a 6 % historical baseline. Under the *low scenario*, hurricane frequency and intensity are kept at current levels.

*Bleaching.* We followed Melbourne-Thomas et al. (2011) and modelled a proportional loss of coral cover of 0.2-60% in each cell (sampled from a uniform distribution), where 60% is the maximum mortality reported due to mass bleaching in the region in 1998 (Steneck and Lang 2003, McField, Bood et al. 2008). We selected a relatively low estimate for minimum mortality, as even periods with more than 8 Degree Heating Weeks or 2 Degree Heating Months often lead to little or no coral mortality (Eakin, Morgan et al. 2010). Indeed, when temperatures exceed 8 Degree Heating Weeks in the Caribbean, mortality can be as high as 80 %, but no mortality occurs in about one third of cases (Eakin, Morgan et al. 2010). We defined two scenarios of bleaching frequency based on the predictions analysed in criterion C and the literature. Average bleaching frequency in the MAR from 2016-2094 is predicted to be 0.48 (1 in 2 years) under no coral adaptation and 0.05 (1 in 20 years) with coral adaptation by 1°C (criterion C). A mass bleaching frequency of 1 in 2 years always resulted in ecosystem collapse regardless of other threat levels, offering little information for risk assessment. Instead, we used a *high scenario* of 1 in 5 years bleaching based on (Donner, Skirving et al. 2005, Melbourne-Thomas, Johnson et al. 2011). We defined the *low scenario* of bleaching of 1 in 20 years.

*Acidification.* A meta-analysis indicated a ~15 % decrease in in coral calcification per unit decrease in aragonite saturation (Chan and Connolly 2013). Friedrich et al. (2012) predicted a decrease of aragonite saturation in the Caribbean of 1.4 units in 2099. Under the *high scenario*, we model acidification as a 21 % decrease in coral growth rate (0.032-0.16 yr^-1^ on epilithic algal communities). Under the *low scenario*, we maintain coral growth rates at current levels (0.04-0.2 yr^-1^ on epilithic algal communities).

*Disease.* Urchin and coral disease have had important effects on the MAR biota (see historical reconstruction and Weil and Rogers (2011)). The incidence and impact of diseases in corals is driven by interactions among hosts, pathogens, and environment (including thermal stress, water quality, and effects of hurricanes) (Weil and Rogers 2011). We attempted to include future scenarios on coral disease incidence and impacts in our analysis, but were unable to do so due to data limitations. Multiple diseases affect Caribbean corals (e.g. white band disease, black band disease, dark spots, and yellow band disease), but the relationship between thermal stress and disease incidence varies among diseases, with some diseases occurring less often under thermal stress (Randall, Jordan-Garza et al. 2014). Current incidence of coral diseases is still largely unknown (Weil and Rogers 2011), limiting our ability to predict the incidence of coral diseases at decadal scales, especially for small geographical areas. We only found one global study attempting to predict the risk of disease outbreaks in corals, by predicting host susceptibility, pathogen abundance, and pathogen virulence (Maynard, van Hooidonk et al. 2015) based on predicted sea surface temperature, but without considering the effects of water quality. In Maynard et al.’s study (2015), host susceptibility was derived from sublethal bleaching stress limits (analogous to our mass bleaching assessment under sucriterion D2a), pathogen abundance was based on one study of white syndromes in the Great Barrier Reef, and pathogen virulence was calculated for only one pathogen (*Vibrio coralliilyticus*) from the Indo-Pacific region. Because each metric was calculated as a binary risk value independent of biological impacts, we could not relate those risk metrics to biological parameters in our model, such as coral mortality or coral recruitment. We concluded that the results from Maynard et al.’s study (2015): i) may not be applicable to the Western Caribbean; ii) showed considerable uncertainty; iii) could not be related to biological parameters in our model; and iv) host susceptibility was already captured in our scenarios of thermal stress. Studies presenting long-term, climate model-based projections of conditions that predict disease in corals are particularly scarce and prone to uncertainty. Further research is necessary for disease risk predictions to be included in ecosystem models, including research on characterizing the relationship between coral hosts, pathogens, and environmental quality, as well as predicting future conditions that may lead to disease occurrence (such as pollution, see subcriterion C2a).

*Initial values.* The model was initiated based on empirical data collected in the MAR in 2013 for assessment under criterion D. Coral cover in 2013 was approximately 16 % in Northern Quintana Roo, Southern Quintana Roo, Chinchorro Bank, and Honduras, and up to 20 % in Belize and Guatemala. We generalized the coral cover to 16 % across all subregions. Herbivorous fish biomass was 30 g/m^2^ in all subregions, piscivorous fish biomass was 8 g/m^2^ in all subregions, and urchin biomass was 1 g/m^2^. We assumed that macroturf cover was 50 % in all subregions and algal cover was 10 % in all subregions.

### *Assessing subcriterion A2a*

To assess future changes in distribution (subcriterion A2a), we used mapped reef locations at 1 km^2^ grain size (Melbourne-Thomas, Johnson et al. 2011) derived from the Millennium Coral Reef Mapping Project’s geomorphological classification (from 30 m Landsat imagery; IMARS 2004). We define occupied cells in the MAR as those with more than 0 %, 1 %, or 5 % live coral cover. We assumed that the future distribution of the MAR cannot extend beyond currently occupied grid cells. The initial distribution size in 2016 of the MAR is 1,359 km^2^. We assumed that the distribution of the MAR could not extend beyond mapped grid cells, due to model constraints. However, there is evidence that corals could extend to more polar latitudes given climate warming (Descombes, Wisz et al. 2015). The extent to which this could be the case in the MAR is unknown. However, given that the ecosystem is listed as Least Concern under subcriterion A2a (see below), possible range extensions of the MAR would not change the Red List assessment under this subcriterion.

We predicted the future distribution of the MAR under the 11 scenarios (Table S11). The decline in geographic distribution was always 0 % based on the 0 % cover collapse threshold, leading to an assessment as Least Concern. The decline in geographic distribution ranged between 4.19 % and 26.05 % across scenarios based on the 1 % collapse threshold, leading to an assessment as Least Concern (Least Concern – Near Threatened). We selected Least Concern as the most plausible category as all likely scenarios (scenarios 5-11) were below the threshold for the Vulnerable category, and only 2 scenarios were within ~ 5 % of the Vulnerable category (thereby qualifying for Near Threatened). The decline in geographic distribution ranged between 7.36 % and 76.31 % based on the 5 % collapse threshold, leading to an assessment as Endangered (Least Concern – Endangered). We selected Endangered as the most likely category as 5 of the most likely scenarios (scenarios 5-11) exceeded thresholds for Endangered. We therefore find that collapse thresholds make large differences to assessment results for criterion A2a.

**Table S11.** Predicted declines in geographic distribution (%) based on different collapse thresholds for coral cover in 2065. The 11 scenarios are defined in the main text.

| Scenario | 0 % coral cover | 1 % coral cover | 5 % coral cover |
| --- | --- | --- | --- |
| Scenario 1 | 0 | 4.27 | 7.36 |
| Scenario 2 | 0 | 4.27 | 7.36 |
| Scenario 3 | 0 | 4.34 | 7.36 |
| Scenario 4 | 0 | 4.27 | 7.36 |
| Scenario 5 | 0 | 23.33 | 75.57 |
| Scenario 6 | 0 | 23.18 | 75.57 |
| Scenario 7 | 0 | 26.05 | 76.31 |
| Scenario 8 | 0 | 25.83 | 76.16 |
| Scenario 9 | 0 | 7.58 | 51.8 |
| Scenario 10 | 0 | 5.96 | 17.44 |
| Scenario 11 | 0 | 4.19 | 9.12 |

### *Assessing subcriterion D2a*

We assessed subcriterion D2a by comparing indicator levels in each 4 km^2^ cell reconstructed by the model in 2016 and 2065. For each year and scenario, we calculated the mean value of the indicator in each cell across the 500 MCMC runs. To obtain relative severity plots for each indicator, we ordered cells in decreasing order of relative severity, and averaged the relative severity over different extents of the ecosystem in 0.07 % increments (due to mapping resolution). The outcome for each scenario was based on the maximum category reached by any combination of relative severity and extent of the ecosystem. The plausible bounds of final assessment were determined by comparing outcomes and likelihoods among scenarios. Increases in at least one of mass bleaching, hurricanes and/or ocean acidification are likely in the next 50 years (Donner 2009, Knutson, McBride et al. 2010, Friedrich, Timmermann et al. 2012), so scenarios 5-11 were used to derive the most likely category and plausible bounds (Appendix S1). The mode of the categories returned by scenarios 5-11was used to derive the most likely category.

Over ≥ 80 % of the ecosystem, relative severity ranged between 24.9 % and 93.1 % (Figure 3). Five out of seven likely scenarios (scenarios 5-11) led to an assessment as Critically Endangered, 1 as Vulnerable, and 1 as Near Threatened (within ~ 5 % of the threshold for the Vulnerable category). All 5 scenarios leading to assessments as Critically Endangered were based on high levels of coral bleaching. We therefore assessed the ecosystem as Critically Endangered (Near Threatened – Critically Endangered) for subcriterion D2a based on coral cover.

We conducted sensitivity analyses for assessments under subcriterion D2a based on different collapse thresholds (Figure S18). Assessments for coral cover ranged from Near Threatened to Critically Endangered for the 0 % collapse threshold, and Vulnerable to Critically Endangered for the 5 % collapse threshold.


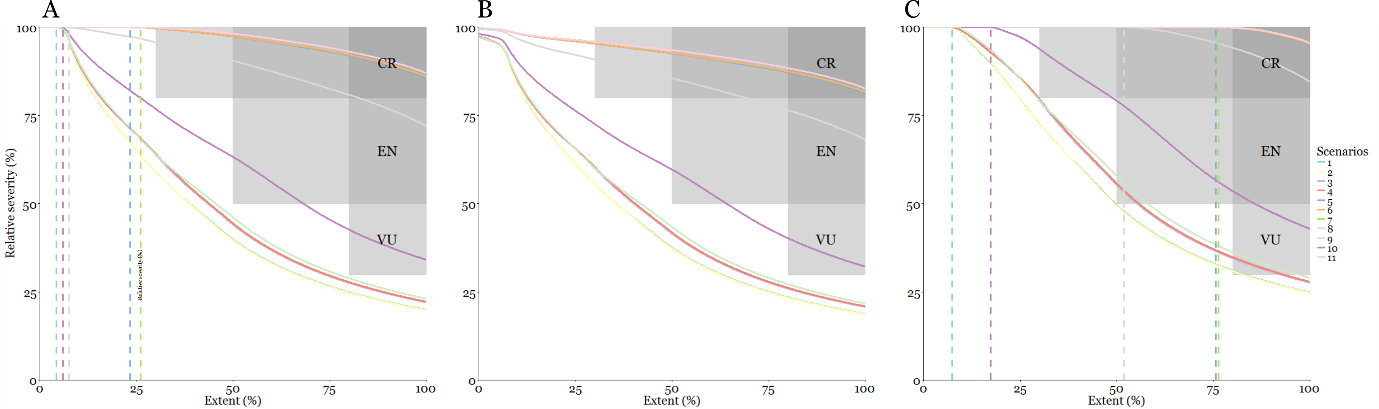


**Figure S18.** Estimated relative severity of decline in coral cover in the Meso-American Reef over the next 50 years (2016-65), projected with the ecosystem model under eleven scenarios of threat. A) Coral cover, with collapse threshold of 1 % coral cover; B) coral cover, with collapse threshold of 0 % coral cover; C) coral cover, with 5 % collapse threshold coral cover. The full lines indicate the relative severity of decline averaged over different extents of the ecosystem (subcriterion D2a), for cells analyzed in decreasing order of relative severity. The hatched vertical lines in panels A and C indicate the decline in spatial extent of the ecosystem under the different scenarios i.e. the percent of cells in the ecosystem with 100% relative severity (subcriterion A2a). The shaded boxes indicate the thresholds for the IUCN Red List of Ecosystems categories (dark grey: Critically Endangered; grey: Endangered; light grey: Vulnerable).

The relative severity of decline in herbivorous fish biomass over ≥ 80 % of the ecosystem ranged between 49.0 % and 82.7 % among scenarios (Figure 3). We determined the most likely category as Endangered because 5 of the 7 likely scenarios (scenarios 5-11) returned this category, with 2 scenarios returning Critically Endangered. We assessed the ecosystem as Endangered (Endangered – Critically Endangered) for subcriterion D2a based on herbivorous fish biomass. Using the lower and upper collapse thresholds for herbivorous fish biomass led to changes in estimates of relative severity < 0.1 %, therefore assessment results were left unchanged by uncertainty in collapse thresholds.

The relative severity of decline in piscivorous fish biomass over ≥ 80 % of the ecosystem ranged between 34.3 % and 81.5 % among scenarios (Figure 3). Out of the likely scenarios (scenarios 5-11), 4 scenarios returned the Critically Endangered category, 1 the Endangered category, and 2 the Vulnerable category. We assess the ecosystem as Critically Endangered (Vulnerable – Critically Endangered) for subcriterion D2a based on piscivorous fish biomass. Using the lower and upper collapse thresholds for piscivorous fish biomass led to changes in estimates of relative severity < 0.1 %, therefore assessment results were left unchanged by uncertainty in collapse thresholds.

### *Assessing criterion E*

Our implementation of 11 scenarios of threats indicated a wide range of collapse probabilities across scenarios and indicators, with non-zero probabilities of collapse for scenarios 5, 6, 7, 8 and 9 when using the 1 % coral cover thresholds for ecosystem collapse (Figure S19).

The probability of ecosystem collapse for all scenarios, time frames and indicators was always zero when the lower thresholds for ecosystem collapse were used (Table S12). In the next 50 years with the higher collapse thresholds, the probability of collapse based on coral cover ranged between 0.014 and 0.844 (Least Concern – Critically Endangered) over the likely scenarios. The probability of ecosystem collapse based on herbivorous fish biomass remained low (Least Concern). The probability of collapse based on piscivorous fish biomass ranged between 0 and 0.572, leading to assessments from Least Concern to Critically Endangered. In the next 100 years with the higher collapse thresholds, assessments for coral cover and piscivorous fish biomass ranged from Least Concern to Vulnerable whilst assessments for herbivorous fish biomass were Least Concern. We conclude that: i) collapse thresholds affect estimated probabilities of collapse under criterion E; and ii) the coral cover and piscivorous fish biomass indicators provide similar information on the probability of ecosystem collapse.


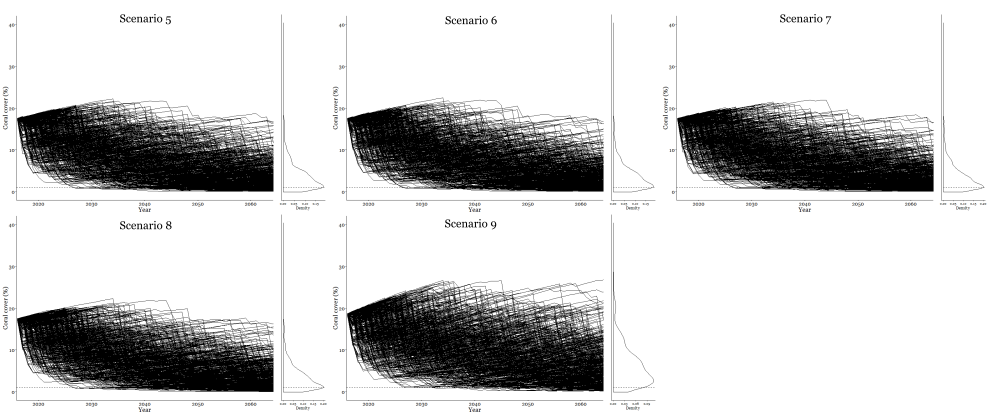


**Figure S19.** Trajectories of coral cover (%) in the next 50 years for five scenarios with non-zero probabilities of collapse, based on the 1 % coral cover collapse threshold. For each scenario, the left plot shows trajectories for 500 Monte Carlo runs, and the right plot indicates the densities of final coral covers in the year 2065.

**Table S12.** Sensitivity analysis of the probability of ecosystem collapse under criterion E for the next 50 and 100 years.

|  | **50 years** | | **100 years** | |
| --- | --- | --- | --- | --- |
| **Coral cover** | 0 % collapse threshold | 5 % collapse threshold | 0 % collapse threshold | 5 % collapse threshold |
| Scenario 1 | 0 | 0.008 | 0 | 0.03 |
| Scenario 2 | 0 | 0.008 | 0 | 0.036 |
| Scenario 3 | 0 | 0.01 | 0 | 0.046 |
| Scenario 4 | 0 | 0.008 | 0 | 0.046 |
| Scenario 5 | 0 | 0.834 | 0 | 0.99 |
| Scenario 6 | 0 | 0.836 | 0 | 0.988 |
| Scenario 7 | 0 | 0.844 | 0 | 0.99 |
| Scenario 8 | 0 | 0.836 | 0 | 0.992 |
| Scenario 9 | 0 | 0.61 | 0 | 0.922 |
| Scenario 10 | 0 | 0.042 | 0 | 0.144 |
| Scenario 11 | 0 | 0.014 | 0 | 0.046 |
| **Herbivorous fish biomass** | 0 g/m^2^ collapse threshold | 9.6 g/m^2^ collapse threshold | 0 g/m^2^ collapse threshold | 9.6 g/m^2^ collapse threshold |
| Scenario 1 | 0 | 0 | 0 | 0 |
| Scenario 2 | 0 | 0 | 0 | 0 |
| Scenario 3 | 0 | 0 | 0 | 0 |
| Scenario 4 | 0 | 0 | 0 | 0 |
| Scenario 5 | 0 | 0 | 0 | 0.002 |
| Scenario 6 | 0 | 0 | 0 | 0.002 |
| Scenario 7 | 0 | 0 | 0 | 0.048 |
| Scenario 8 | 0 | 0.006 | 0 | 0.052 |
| Scenario 9 | 0 | 0 | 0 | 0.006 |
| Scenario 10 | 0 | 0 | 0 | 0 |
| Scenario 11 | 0 | 0 | 0 | 0 |
| **Piscivorous fish biomass** | 0 g/m^2^ collapse threshold | 4.2 g/m^2^ collapse threshold | 0 g/m^2^ collapse threshold | 4.2 g/m^2^ collapse threshold |
| Scenario 1 | 0 | 0 | 0 | 0 |
| Scenario 2 | 0 | 0 | 0 | 0 |
| Scenario 3 | 0 | 0.002 | 0 | 0.002 |
| Scenario 4 | 0 | 0 | 0 | 0.004 |
| Scenario 5 | 0 | 0.482 | 0 | 0.88 |
| Scenario 6 | 0 | 0.488 | 0 | 0.878 |
| Scenario 7 | 0 | 0.572 | 0 | 0.916 |
| Scenario 8 | 0 | 0.554 | 0 | 0.91 |
| Scenario 9 | 0 | 0.284 | 0 | 0.638 |
| Scenario 10 | 0 | 0.002 | 0 | 0.008 |
| Scenario 11 | 0 | 0 | 0 | 0.002 |

# **References**

Adam, T. C., D. E. Burkepile, B. I. Ruttenberg and M. J. Paddack (2015). "Herbivory and the resilience of Caribbean coral reefs: knowledge gaps and implications for management." Marine Ecology Progress Series **520**: 1-20.

Addison, P. F. E., L. Rumpff, S. S. Bau, J. M. Carey, Y. E. Chee, F. C. Jarrad, M. F. McBride and M. A. Burgman (2013). "Practical solutions for making models indispensable in conservation decision-making." Diversity and Distributions **19**(5-6): 490-502.

Andersson, A. J. and D. Gledhill (2013). "Ocean acidification and coral reefs: effects on breakdown, dissolution, and net ecosystem calcification." Annual Review of Marine Science **5**: 321-348.

Arnold, C. P. J. and C. H. Dey (1986). "Observing-systems simulation experiments: Past, present, and future." Bulletin of the American Meteorological Society **67**(6): 687-695.

Aronson, R. B. and W. F. Precht (2001). White-band disease and the changing face of Caribbean coral reefs. The Ecology and Etiology of Newly Emerging Marine Diseases, Springer**:** 25-38.

Arrivillaga, A. and M. A. Garcia (2013). "Status of coral reefs of the Mesoamerican Barrier Reef systems project region, and reefs of El Salvador, Nicaragua and the Pacific coasts of Mesoamerica."

Bauer, J. C. (1980). "Observations on Geographical Variations in Population Density of the Echinoid Diadema Antillarum within the Western North Atlantic." Bulletin of Marine Science **30**(2): 509-515.

Biggs, R., C. Raudsepp-Hearne, C. Atkinson-Palombo, E. Bohensky, E. Boyd, G. Cundill, H. Fox, S. Ingram, K. Kok and S. Spehar (2007). "Linking futures across scales: a dialog on multiscale scenarios." Ecology and Society **12**(1): 17.

Bland, L. M., D. A. Keith, R. M. Miller, N. J. Murray and J. P. Rodríguez (2016). Guidelines for the application of IUCN Red List of Ecosystems Categories and Criteria, Version 1.0. Gland, Switzerland, IUCN.

Boldt, J. L., R. Martone, J. Samhouri, R. I. Perry, S. Itoh, I. K. Chung, M. Takahashi and N. Yoshle (2014). "Developing Ecosystem Indicators for Responses to Multiple Stressors." Oceanography **27**(4): 116-133.

Box, S. J. and P. J. Mumby (2007). "Effect of macroalgal competition on growth and survival of juvenile Caribbean corals." Marine Ecology Progress Series **342**: 139-149.

Brown-Saracino, J., P. Peckol, H. A. Curran and M. L. Robbart (2007). "Spatial variation in sea urchins, fish predators, and bioerosion rates on coral reefs of Belize." Coral Reefs **26**(1): 71-78.

Burke, L. and Z. Sugg (2006). "Hydrologic Modeling of Watersheds Discharging Adjacent to the Mesoamerican Reef Analysis Summary-December 1, 2006."

Burnham, K. P. and D. R. Anderson (2002). Model selection and multimodel inference: a practical information-theoretic approach, Springer Science & Business Media.

Bythell, J., E. Gladfelter and M. Bythell (1993). "Chronic and catastrophic natural mortality of three common Caribbean reef corals." Coral Reefs **12**(3-4): 143-152.

Cao, L. and K. Caldeira (2008). "Atmospheric CO2 stabilization and ocean acidification." Geophysical Research Letters **35**(19).

Carilli, J. E., N. G. Prouty, K. A. Hughen and R. D. Norris (2009). "Century-scale records of land-based activities recorded in Mesoamerican coral cores." Marine Pollution Bulletin **58**(12): 1835-1842.

Carricart-Ganivet, J. P., N. Cabanillas-Teran, I. Cruz-Ortega and P. Blanchon (2012). "Sensitivity of calcification to thermal stress varies among genera of massive reef-building corals." PLoS One **7**(3): e32859.

Casey, K. S., T. B. Brandon, P. Cornillon and R. Evans (2010). The past, present, and future of the AVHRR Pathfinder SST program. Oceanography from space, Springer**:** 273-287.

Chan, N. and S. R. Connolly (2013). "Sensitivity of coral calcification to ocean acidification: a meta‐analysis." Global change biology **19**(1): 282-290.

Cheal, A., G. Coleman, S. Delean, I. Miller, K. Osborne and H. Sweatman (2002). "Responses of coral and fish assemblages to a severe but short-lived tropical cyclone on the Great Barrier Reef, Australia." Coral Reefs **21**(2): 131-142.

Chérubin, L., C. Kuchinke and C. Paris (2008). "Ocean circulation and terrestrial runoff dynamics in the Mesoamerican region from spectral optimization of SeaWiFS data and a high resolution simulation." Coral Reefs **27**(3): 503-519.

Chornesky, E. A. and E. C. Peters (1987). "Sexual reproduction and colony growth in the scleractinian coral Porites astreoides." The Biological Bulletin **172**(2): 161-177.

Cowen, R., C. Paris and A. Srinivasan (2006). "Scaling of connectivity in marine populations." Science **311**(5760): 522-527.

Cramer, K. L., J. B. Jackson, C. V. Angioletti, J. Leonard‐Pingel and T. P. Guilderson (2012). "Anthropogenic mortality on coral reefs in Caribbean Panama predates coral disease and bleaching." Ecology letters **15**(6): 561-567.

De’ath, G., K. E. Fabricius, H. Sweatman and M. Puotinen (2012). "The 27–year decline of coral cover on the Great Barrier Reef and its causes." Proceedings of the National Academy of Sciences **109**(44): 17995-17999.

Descombes, P., M. S. Wisz, F. Leprieur, V. Parravicini, C. Heine, S. M. Olsen, D. Swingedouw, M. Kulbicki, D. Mouillot and L. Pellissier (2015). "Forecasted coral reef decline in marine biodiversity hotspots under climate change." Global Change Biology **21**(7): 2479-2487.

Doney, S. C., V. J. Fabry, R. A. Feely and J. A. Kleypas (2009). "Ocean acidification: the other CO2 problem." Marine Science **1**.

Donner, S. D. (2009). "Coping with Commitment: Projected Thermal Stress on Coral Reefs under Different Future Scenarios." PLoS ONE **4**(6): e5712.

Donner, S. D., T. R. Knutson and M. Oppenheimer (2007). "Model-based assessment of the role of human-induced climate change in the 2005 Caribbean coral bleaching event." Proceedings of the National Academy of Sciences **104**(13): 5483-5488.

Donner, S. D., W. J. Skirving, C. M. Little, M. Oppenheimer and O. Hoegh‐Guldberg (2005). "Global assessment of coral bleaching and required rates of adaptation under climate change." Global Change Biology **11**(12): 2251-2265.

Eakin, C. M., J. A. Morgan, S. F. Heron, T. B. Smith, G. Liu, L. Alvarez-Filip, B. Baca, E. Bartels, C. Bastidas and C. Bouchon (2010). "Caribbean corals in crisis: record thermal stress, bleaching, and mortality in 2005." PloS one **5**(11): e13969.

Edmunds, P. J. (2007). "Evidence for a decadal-scale decline in the growth rates of juvenile scleractinian corals." Marine Ecology Progress Series **341**: 1-13.

Emanuel, K. A. (2013). "Downscaling CMIP5 climate models shows increased tropical cyclone activity over the 21st century." Proceedings of the National Academy of Sciences **110**(30): 12219-12224.

Fabricius, K. (2011). Nutrient Pollution/Eutrophication. Encyclopedia of modern coral reefs, Springer**:** 722-731.

Ferrari, R., M. Gonzalez-Rivero, J. C. Ortiz and P. J. Mumby (2012). "Interaction of herbivory and seasonality on the dynamics of Caribbean macroalgae." Coral Reefs **31**(3): 683-692.

Foden, W. B., S. H. M. Butchart, S. N. Stuart, J.-C. Vié, H. R. Akçakaya, A. Angulo, L. M. DeVantier, A. Gutsche, E. Turak, L. Cao, S. D. Donner, V. Katariya, R. Bernard, R. A. Holland, A. F. Hughes, S. E. O’Hanlon, S. T. Garnett, Ç. H. Şekercioğlu and G. M. Mace (2013). "Identifying the World's Most Climate Change Vulnerable Species: A Systematic Trait-Based Assessment of all Birds, Amphibians and Corals." PLoS ONE **8**(6): e65427.

Friedrich, T., A. Timmermann, A. Abe-Ouchi, N. Bates, M. Chikamoto, M. Church, J. Dore, D. Gledhill, M. Gonzalez-Davila and M. Heinemann (2012). "Detecting regional anthropogenic trends in ocean acidification against natural variability." Nature Climate Change **2**(3): 167-171.

Fulton, E. A., A. D. M. Smith and A. E. Punt (2005). "Which ecological indicators can robustly detect effects of fishing?" ICES Journal of Marine Science: Journal du Conseil **62**(3): 540-551.

Fulton, S., L. Bourillón, C. Ribot, J. Caamal, C. García, E. Flores, W. Heyman and M. Olivares (2014). "Fishermen investing in a network of fish refuges (No-Take Zones) in Quintana Roo, Mexico." ENHANCING STEWARDSHIP IN SMALL-‐SCALE FISHERIES: PRACTICES AND PERSPECTIVES: 138.

Fung, T., R. M. Seymour and C. R. Johnson (2011). "Alternative stable states and phase shifts in coral reefs under anthropogenic stress." Ecology **92**(4): 967-982.

Garcia-Salgado, M., G. Nava-Martinez, N. Bood, M. Mcfield, A. Molina-Ramirez, B. Yañez-Rivera, N. Jacobs, B. Shank, M. Vasquez and I. Majil (2008). "Status of coral reefs in the Mesoamerican region." Status of coral reefs of the world: 253-264.

Gardner, T. A., I. M. Cote, J. A. Gill, A. Grant and A. R. Watkinson (2005). "Hurricanes and Caribbean coral reefs: impacts, recovery patterns, and role in long-term decline." Ecology **86**(1): 174-184.

Gledhill, D. K., R. Wanninkhof, F. J. Millero and M. Eakin (2008). "Ocean acidification of the greater Caribbean region 1996–2006." Journal of Geophysical Research: Oceans (1978–2012) **113**(C10).

Gordon, C., C. Cooper, C. A. Senior, H. Banks, J. M. Gregory, T. C. Johns, J. F. Mitchell and R. A. Wood (2000). "The simulation of SST, sea ice extents and ocean heat transports in a version of the Hadley Centre coupled model without flux adjustments." Climate dynamics **16**(2-3): 147-168.

Goreau, T. F. (1964). "Mass expulsion of zooxanthellae from Jamaican reef communities after Hurricane Flora." Science **145**(3630): 383-386.

Guinotte, J., R. Buddemeier and J. Kleypas (2003). "Future coral reef habitat marginality: temporal and spatial effects of climate change in the Pacific basin." Coral Reefs **22**(4): 551-558.

Halls, A., R. Burn and S. Abeyasekera (2002). Interdisciplinary analysis for adaptive co-management, Final Technical Report to DFID. MRAG Ltd, London.

Healthy Reefs Initiative (2015). MesoAmerican Reef; A Report Card of Ecosystem Health**:** 31 pp.

Hoegh-Guldberg, O., P. J. Mumby, A. J. Hooten, R. S. Steneck, P. Greenfield, E. Gomez, C. D. Harvell, P. F. Sale, A. J. Edwards, K. Caldeira, N. Knowlton, C. M. Eakin, R. Iglesias-Prieto, N. Muthiga, R. H. Bradbury, A. Dubi and M. E. Hatziolos (2007). "Coral Reefs Under Rapid Climate Change and Ocean Acidification." Science **318**(5857): 1737-1742.

Hughes, T. P., A. H. Baird, D. R. Bellwood, M. Card, S. R. Connolly, C. Folke, R. Grosberg, O. Hoegh-Guldberg, J. Jackson and J. Kleypas (2003). "Climate change, human impacts, and the resilience of coral reefs." science **301**(5635): 929-933.

Huston, M. (1985). "Variation in coral growth rates with depth at Discovery Bay, Jamaica." Coral Reefs **4**(1): 19-25.

IMARS (2004). Millenium Coral Reef Mapping Project. I. f. M. R. S. University of South Florida. St Petersburg, FL, USA.

IUCN (2016). An Introduction to the IUCN Red List of Ecosystems: The Categories and Criteria for

Assessing Risks to Ecosystems. Gland, Switzerland, IUCN**:** vi + 14pp.

Jackson, J., M. Donovan, K. Cramer and V. Lam (2014). Status and trends of Caribbean coral reefs: 1970-2012, Global Coral Reef Monitoring Network.

Karr, K. A., R. Fujita, B. S. Halpern, C. V. Kappel, L. Crowder, K. A. Selkoe, P. M. Alcolado and D. Rader (2014). "Thresholds in Caribbean coral reefs: Implications for ecosystem-based fishery management." Journal of Applied Ecology: n/a-n/a.

Keith, D. a., J. P. Rodríguez, K. M. Rodríguez-Clark, E. Nicholson, K. Aapala, A. Alonso, M. Asmussen, S. Bachman, A. Basset, E. G. Barrow, J. S. Benson, M. J. Bishop, R. Bonifacio, T. M. Brooks, M. a. Burgman, P. Comer, F. a. Comín, F. Essl, D. Faber-Langendoen, P. G. Fairweather, R. J. Holdaway, M. Jennings, R. T. Kingsford, R. E. Lester, R. Mac Nally, M. a. McCarthy, J. Moat, M. a. Oliveira-Miranda, P. Pisanu, B. Poulin, T. J. Regan, U. Riecken, M. D. Spalding and S. Zambrano-Martínez (2013). "Scientific foundations for an IUCN Red List of ecosystems." PloS one **8**(5): e62111-e62111.

Kennedy, Emma V., Chris T. Perry, Paul R. Halloran, R. Iglesias-Prieto, Christine H. L. Schönberg, M. Wisshak, Armin U. Form, Juan P. Carricart-Ganivet, M. Fine, C. M. Eakin and Peter J. Mumby (2013). "Avoiding Coral Reef Functional Collapse Requires Local and Global Action." Current Biology **23**(10): 912-918.

Knapp, K. R., S. Applequist, H. J. Diamond, J. P. Kossin, M. Kruk and C. Schreck (2010). NCDC International Best Track Archive for Climate Stewardship (IBTrACS) Project, Version 3. N. N. C. D. Center.

Knutson, T. R., J. L. McBride, J. Chan, K. Emanuel, G. Holland, C. Landsea, I. Held, J. P. Kossin, A. Srivastava and M. Sugi (2010). "Tropical cyclones and climate change." Nature Geoscience **3**(3): 157-163.

Knutson, T. R., J. J. Sirutis, G. A. Vecchi, S. Garner, M. Zhao, H.-S. Kim, M. Bender, R. E. Tuleya, I. M. Held and G. Villarini (2013). "Dynamical Downscaling Projections of Twenty-First-Century Atlantic Hurricane Activity: CMIP3 and CMIP5 Model-Based Scenarios." Journal of Climate **26**(17): 6591-6617.

Koslow, J., K. Aiken, S. Auil and A. Clementson (1994). "Catch and effort analysis of the reef fisheries of Jamaica and Belize." Fishery Bulletin **92**(4): 737-747.

Kuffner, I. B., L. J. Walters, M. A. Becerro, V. J. Paul, R. Ritson-Williams and K. S. Beach (2006). "Inhibition of coral recruitment by macroalgae and cyanobacteria." Marine Ecology Progress Series **323**: 107-117.

Landsea, C. W., G. A. Vecchi, L. Bengtsson and T. R. Knutson (2010). "Impact of Duration Thresholds on Atlantic Tropical Cyclone Counts*." Journal of Climate **23**(10): 2508-2519.

Langdon, C. and M. Atkinson (2005). "Effect of elevated pCO2 on photosynthesis and calcification of corals and interactions with seasonal change in temperature/irradiance and nutrient enrichment." Journal of Geophysical Research: Oceans (1978–2012) **110**(C9).

Lessios, H. (1988). "Mass mortality of Diadema antillarum in the Caribbean: what have we learned?" Annual Review of Ecology and Systematics: 371-393.

Liu, G., A. E. Strong, W. Skirving and L. F. Arzayus (2006). Overview of NOAA coral reef watch program’s near-real time satellite global coral bleaching monitoring activities. Proceedings of the 10th international coral reef symposium: Okinawa.

Lough, J. M. (2011). Climate change and coral reefs. Encyclopedia of Modern Coral Reefs, Springer**:** 198-210.

Mann, M. E., J. D. Woodruff, J. P. Donnelly and Z. Zhang (2009). "Atlantic hurricanes and climate over the past 1,500 years." Nature **460**(7257): 880-883.

Maynard, J., R. van Hooidonk, C. M. Eakin, M. Puotinen, M. Garren, G. Williams, S. F. Heron, J. Lamb, E. Weil, B. Willis and C. D. Harvell (2015). "Projections of climate conditions that increase coral disease susceptibility and pathogen abundance and virulence." Nature Clim. Change **5**(7): 688-694.

McClenachan, L., J. B. Jackson and M. J. Newman (2006). "Conservation implications of historic sea turtle nesting beach loss." Frontiers in Ecology and the Environment **4**(6): 290-296.

McField, M. (2000). Influence of disturbance on coral reef community structure in Belize. Proceedings of the Ninth International Coral Reef Symposium.

McField, M., N. Bood, A. Fonseca, A. Arrivillaga, A. Franquesa Rinos and R. M. Loreto Viruel (2008). Status of the Mesoamerican Reef after the 2005 coral bleaching event. Status of Caribbean coral reefs after bleaching and hurricanes in 2005. Townsville, Queensland, Australia, Global Reef Monitoring Network and Rainforest Research Centre.

McField, M. and P. Kramer (2007). "Healthy reefs for healthy people: A guide to indicators of reef health and social well-being in the Mesoamerican Reef Region." Miami: Healthy Reefs Initiative.

McField, M. and P. Kramer (2007). "Healthy reefs for healthy people: A guide to indicators of reef health and social well-being in the Mesoamerican Reef Region." With contributions by M. Gorrez and M. McPherson.

McWilliams, J. P., I. M. Côté, J. A. Gill, W. J. Sutherland and A. R. Watkinson (2005). "ACCELERATING IMPACTS OF TEMPERATURE-INDUCED CORAL BLEACHING IN THE CARIBBEAN." Ecology **86**(8): 2055-2060.

Melbourne-Thomas, J., C. R. Johnson and E. Fulton (2011). "Characterizing sensitivity and uncertainty in a multiscale model of a complex coral reef system." Ecological Modelling **222**(18): 3320-3334.

Melbourne-Thomas, J., C. R. Johnson and E. A. Fulton (2011). "Regional-scale scenario analysis for the Meso-American Reef system: Modelling coral reef futures under multiple stressors." Ecological Modelling **222**(10): 1756-1770.

Melbourne-Thomas, J., C. R. Johnson, T. Fung, R. M. Seymour, L. M. Chérubin, J. E. Arias-González and E. A. Fulton (2011). "Regional-scale scenario modeling for coral reefs: a decision support tool to inform management of a complex system." Ecological Applications **21**(4): 1380-1398.

Melbourne-Thomas, J., C. R. Johnson, P. Perez, J. Eustache, E. A. Fulton and D. Cleland (2011). "Coupling biophysical and socioeconomic models for coral reef systems in Quintana Roo, Mexican Caribbean." Ecology and Society **16**(3): 23.

Mumby, P. J. (2006). "The impact of exploiting grazers (Scaridae) on the dynamics of Caribbean coral reefs." Ecological applications **16**(2): 747-769.

Niemeijer, D. and R. S. de Groot (2008). "A conceptual framework for selecting environmental indicator sets." Ecological indicators **8**(1): 14-25.

Nyberg, J., B. A. Malmgren, A. Winter, M. R. Jury, K. H. Kilbourne and T. M. Quinn (2007). "Low Atlantic hurricane activity in the 1970s and 1980s compared to the past 270 years." Nature **447**(7145): 698-701.

Olsen, E., G. Fay, S. Gaichas, R. Gamble, S. Lucey and J. S. Link (2016). "Ecosystem Model Skill Assessment. Yes We Can!" PLoS ONE **11**(1): e0146467.

Pandolfi, J. M., R. H. Bradbury, E. Sala, T. P. Hughes, K. A. Bjorndal, R. G. Cooke, D. McArdle, L. McClenachan, M. J. Newman and G. Paredes (2003). "Global trajectories of the long-term decline of coral reef ecosystems." Science **301**(5635): 955-958.

Pandolfi, J. M., S. R. Connolly, D. J. Marshall and A. L. Cohen (2011). "Projecting Coral Reef Futures Under Global Warming and Ocean Acidification." Science **333**(6041): 418-422.

Pandolfi, J. M. and J. B. Jackson (2001). "Community structure of Pleistocene coral reefs of Curaçao, Netherlands Antilles." Ecological monographs **71**(1): 49-67.

Paris, C. and L. Chérubin (2008). "River-reef connectivity in the Meso-American Region." Coral Reefs **27**(4): 773-781.

Paris, C., L. Cherubin and R. Cowen (2007). "Surfing, diving or spinning: effects on population connectivity." Marine Ecology Progress Series **347**: 285-300.

Randall, C. J., A. G. Jordan-Garza, E. M. Muller and R. van Woesik (2014). "Relationships between the history of thermal stress and the relative risk of diseases of Caribbean corals." Ecology **95**(7): 1981-1994.

Rasher, D. B. and M. E. Hay (2010). "Chemically rich seaweeds poison corals when not controlled by herbivores." Proceedings of the National Academy of Sciences **107**(21): 9683-9688.

Rayner, N. A., D. E. Parker, E. B. Horton, C. K. Folland, L. V. Alexander, D. P. Rowell, E. C. Kent and A. Kaplan (2003). "Global analyses of sea surface temperature, sea ice, and night marine air temperature since the late nineteenth century." Journal of Geophysical Research: Atmospheres **108**(D14): n/a-n/a.

Risk, M. J. and E. Edinger (2011). Impacts of sediment on coral reefs. Encyclopedia of Modern Coral Reefs, Springer**:** 575-586.

Rogers, C. S. (1990). "Responses of coral reefs and reef organisms to sedimentation." Marine ecology progress series. Oldendorf **62**(1): 185-202.

Scott, E., N. Serpetti, J. Steenbeek and J. J. Heymans (2016). "A Stepwise Fitting Procedure for automated fitting of Ecopath with Ecosim models." SoftwareX.

Sheppard, C. and R. Rioja-Nieto (2005). "Sea surface temperature 1871–2099 in 38 cells in the Caribbean region." Marine environmental research **60**(3): 389-396.

Sheppard, C. R. (2003). "Predicted recurrences of mass coral mortality in the Indian Ocean." Nature **425**(6955): 294-297.

Shinn, E. (1966). "Coral growth-rate, an environmental indicator." Journal of Paleontology: 233-240.

Spalding, M. D., H. E. Fox, G. R. Allen, N. Davidson, Z. A. FerdaÑA, M. A. X. Finlayson, B. S. Halpern, M. A. Jorge, A. L. Lombana, S. A. Lourie, K. D. Martin, E. McManus, J. Molnar, C. A. Recchia and J. Robertson (2007). "Marine Ecoregions of the World: A Bioregionalization of Coastal and Shelf Areas." BioScience **57**(7): 573-583.

Steneck, R. S. and J. C. Lang (2003). "Rapid assessment of Mexico's Yucatan reef in 1997 and 1999: Pre- and post-1998 mass bleaching and Hurricane Mitch(stony corals, algae and fishes)." Atoll Research Bulletin **496**: 294-317.

Stow, C. A., J. Jolliff, D. J. McGillicuddy, S. C. Doney, J. I. Allen, M. A. Friedrichs, K. A. Rose and P. Wallhead (2009). "Skill assessment for coupled biological/physical models of marine systems." Journal of Marine Systems **76**(1): 4-15.

Suter, G. W. (2006). Ecological risk assessment, CRC press.

Van Oppen, M. J. and J. M. Lough (2008). Coral bleaching: patterns, processes, causes and consequences, Springer Science & Business Media.

Vecchi, G. A. and T. R. Knutson (2008). "On Estimates of Historical North Atlantic Tropical Cyclone Activity*." Journal of Climate **21**(14): 3580-3600.

Watermeyer, K., L. Shannon and C. Griffiths (2008). "Changes in the trophic structure of the southern Benguela before and after the onset of industrial fishing." African Journal of Marine Science **30**(2): 351-382.

Weijerman, M., E. A. Fulton, I. C. Kaplan, R. Gorton, R. Leemans, W. M. Mooij and R. E. Brainard (2015). "An integrated coral reef ecosystem model to support resource management under a changing climate." PloS one **10**(12): e0144165.

Weil, E. and C. S. Rogers (2011). Coral reef diseases in the Atlantic-Caribbean. Coral reefs: an ecosystem in transition, Springer**:** 465-491.
